# Fundamental Identifiability Limits in Molecular Epidemiology

**DOI:** 10.1093/molbev/msab149

**Published:** 2021-05-19

**Authors:** Stilianos Louca, Angela McLaughlin, Ailene MacPherson, Jeffrey B Joy, Matthew W Pennell

**Affiliations:** 1 Department of Biology, University of Oregon, Eugene, OR, USA; 2 Institute of Ecology and Evolution, University of Oregon, Eugene, OR, USA; 3 British Columbia Centre for Excellence in HIV/AIDS, Vancouver, BC, Canada; 4 Bioinformatics, University of British Columbia, Vancouver, BC, Canada; 5 Biodiversity Research Centre, University of British Columbia, Vancouver, BC, Canada; 6 Department of Zoology, University of British Columbia, Vancouver, BC, Canada; 7 Department of Ecology and Evolutionary Biology, University of Toronto, Toronto, ON, Canada; 8 Department of Medicine, University of British Columbia, Vancouver, BC, Canada

**Keywords:** epidemiology, phylogenetics, statistical inference, birth-death-sampling model

## Abstract

Viral phylogenies provide crucial information on the spread of infectious diseases, and many studies fit mathematical models to phylogenetic data to estimate epidemiological parameters such as the effective reproduction ratio (*R_e_*) over time. Such phylodynamic inferences often complement or even substitute for conventional surveillance data, particularly when sampling is poor or delayed. It remains generally unknown, however, how robust phylodynamic epidemiological inferences are, especially when there is uncertainty regarding pathogen prevalence and sampling intensity. Here, we use recently developed mathematical techniques to fully characterize the information that can possibly be extracted from serially collected viral phylogenetic data, in the context of the commonly used birth-death-sampling model. We show that for any candidate epidemiological scenario, there exists a myriad of alternative, markedly different, and yet plausible “congruent” scenarios that cannot be distinguished using phylogenetic data alone, no matter how large the data set. In the absence of strong constraints or rate priors across the entire study period, neither maximum-likelihood fitting nor Bayesian inference can reliably reconstruct the true epidemiological dynamics from phylogenetic data alone; rather, estimators can only converge to the “congruence class” of the true dynamics. We propose concrete and feasible strategies for making more robust epidemiological inferences from viral phylogenetic data.

## Introduction

For rapidly evolving pathogens, such as RNA viruses, genetic diversity accumulates on the same timescale as transmission (Drummond et al. 2003). Thus, pathogen genealogies reconstructed from patient samples can provide valuable information on the transmission dynamics of diseases (Pybus and Rambaut 2009; Volz et al. 2013). As sequencing technology and computational methods continue to improve, such phylogenetic approaches (Grenfell et al. 2004; Bouckaert et al. 2019) are increasingly being used to help inform public health policy during ongoing epidemics, such as during the 2013–2016 Ebolavirus outbreak (Holmes et al. 2016), the 2015–2016 expansion of Zika virus (Faria et al. 2016), and the SARS-CoV-2 pandemic that began in 2019 (Worobey et al. 2020). One of the most popular mathematical frameworks used for such phylodynamic inferences is the birth-death (BD) model (Stadler et al. 2012, 2013; Kühnert et al. 2014), variants of which are also used to reconstruct macroevolutionary dynamics (Morlon 2014). BD models are typically either fitted to a given time-calibrated phylogeny (henceforth timetree) or jointly estimated with the timetree from molecular sequences, to obtain estimates of the birth or speciation rate (λ, corresponding to transmission between hosts in epidemiology or speciation in macroevolution), the death or extinction rate (μ, host death or recovery in epidemiology; extinction in macroevolution), and the sampling rate (ψ, number of pathogen lineages sampled per time and per extant lineage) through time. From these rates, one can calculate critical epidemiological parameters such as the effective reproduction ratio $R_{e}=\lambda /(\mu +\psi )$ (Stadler et al. 2012, 2013).

Despite the increasing importance of phylodynamic estimates to public health policy, it is generally unknown precisely what information we can hope to extract from phylogenetic data and how robust these estimates are expected to be, particularly when all rates exhibit temporal variation and are unknown a priori. In macroevolutionary BD models, where only extant lineages are sampled, much work has been done to understand identifiability limits (Kendall 1948; Nee, Holmes, et al. 1994; Nee, May, et al. 1994; Stadler 2009; Stadler and Steel 2019; Louca and Pennell 2020). That work has shown that if the birth rate λ and death rate μ vary through time, there are a vast number of alternative plausible combinations of λ and μ that could explain any extant timetree (i.e., comprising only extant species) equally well. Such “congruent” scenarios cannot be distinguished from one another statistically, even in the presence of an infinitely large and completely sampled timetree—in other words, it is impossible to design asymptotically consistent estimation methods for λ and μ based on extant timetrees alone and without strong prior assumptions (Louca and Pennell 2020).

It is largely unknown to what extent and in what form such congruency issues exist in epidemiological BD models, that is, with continuous sampling through time. Although some relatively minor parameter correlations have been known for special cases (Stadler et al. 2013; Gavryushkina et al. 2014), an understanding of general identifiability limits is lacking, and the macroevolutionary case has taught us that parameter correlations known for specific cases might severely underestimate the full extent of the problem. This question is nontrivial: although epidemiological BD models appear similar to macroevolutionary BD models, they are more complex because pathogen sequences are typically not sampled at a single final time point. Samples obtained serially through time provide additional information on an epidemic; however, new uncertainty is introduced when the sampling rate (ψ) is unknown and, especially, when it varies over time.

Here, we provide a definite answer to the above questions and demonstrate that, similar to the macroevolutionary case, there are fundamental limits to how much information can be gleaned from timetrees sampled through time in the absence of strong additional constraints. Specifically, we prove mathematically that for any one hypothesized birth-death-sampling (BDS) scenario—that is, with specific time-varying λ, μ and ψ, there exist an infinite number of alternative, markedly different, and yet plausible BDS scenarios that are statistically indistinguishable from the hypothesized scenario, even with infinitely large phylogenetic data sets. Using simulations and real sequence data from an HIV outbreak in Northern Alberta, Canada, we demonstrate that this identifiability issue means that many epidemiological inferences from phylogenetic data alone may not be as well-supported as previously thought. Fortunately, and in contrast to the macroevolutionary case, we are able to identify concrete and feasible approaches toward resolving these issues in practice.

## Identifiability of General BDS Models

Our starting point is the general BDS model with arbitrary time-dependent birth rate *λ*, death rate *μ*, and sampling rate *ψ*, where we make the common assumptions that sampled lineages (tips in the phylogeny) are immediately removed from the pool of extant lineages and that branching events correspond to transmission events (MacPherson et al. 2021). We use the term “BDS scenario” (or “epidemiological scenario”) to refer to a specific choice of profiles over time for the parameters *λ*, *μ*, and *ψ*. Using mathematical techniques similar to those developed for macroevolutionary models (Louca and Pennell 2020), we find that the likelihood of any timetree under a given epidemiological scenario is entirely determined by only two model parameters, called the pulled birth rate (denoted $\tilde{\lambda }$) and pulled sampling rate (denoted $\tilde{\psi }$). Here, $\tilde{\lambda }$ is equal to the birth rate, *λ*, multiplied by the probability that a lineage is included in the phylogeny, whereas $\tilde{\psi }$ is equal to the sampling rate, *ψ*, divided by the probability that a lineage is included in the phylogeny (overview of symbols in table 1). The $\tilde{\lambda }$ and $\tilde{\psi }$ are thus the expected occurrence rate of internal nodes and tips, respectively, over time when divided over the current number of lineages in the tree and in the limit of infinitely large trees (proof in supplementary S.1.3, Supplementary Material online). Note that $\tilde{\lambda }$ and $\tilde{\psi }$ are purely theoretical properties of the BDS scenario that can be calculated from *λ*, *μ*, and *ψ*, and do not depend on any particular data set. We henceforth call any two BDS scenarios congruent if they have the same pulled birth rate $\tilde{\lambda }$ and the same pulled sampling rate $\tilde{\psi }$. By extension, the congruence class of any BDS scenario henceforth refers to the set of all congruent BDS scenarios. Any two congruent BDS scenarios generate timetrees with the same probability distribution (supplementary S.1.3 and S.1.4, Supplementary Material online) and will yield identical likelihoods for any given timetree. This means that there is no way to distinguish between two congruent scenarios solely based on the properties of sampled timetrees, no matter how large. This result is analogous to that of macroevolutionary BD models (i.e., when all the tips are contemporaneous), where the probability distribution of generated timetrees is entirely determined by the pulled birth rate, and any two scenarios with identical pulled birth rates are statistically indistinguishable. This result is also analogous to identifiability issues discovered for population demographic models, where markedly different population demographies can generate the same sample frequency spectrum for arbitrarily large sample sizes (Myers et al. 2008; Bhaskar and Song 2014).

**Table 1:** Overview of Main BDS Parameters Discussed.

Symbol	Description	Definition	Congr.-Invariant
*λ*	Birth (speciation) rate	—	No
*μ*	Death (extinction) rate	—	No
*ψ*	Sampling rate	—	No
$R_{e}$	Effective reproduction ratio	λ/(μ+ψ)	No
*δ*	Removal (become uninfectious) rate	μ+ψ	No
*S*	Sampling proportion	ψ/(μ+ψ)	No
*E*	Probability of a lineage missing from the phylogeny	Equation (3) in supplementary S.1.1, SupplementaryMaterial online	No
$\tilde{\lambda }$	Pulled birth rate	(1−E)·λ	Yes
$\tilde{\psi }$	Pulled sampling rate	ψ/(1−E)	Yes
$\tilde{r}$	Pulled diversification rate	λ−μ−ψ+(1/λ)dλ/dτ	Yes
$\tilde{\beta }$	Deterministic branching density	Equation (16) in supplementary S.1.2, SupplementaryMaterial online	Yes
$\tilde{\sigma }$	Deterministic sampling density	Equation (17) in supplementary S.1.2, Supplementary Materialonline	Yes
$\tilde{M}$	Normalized deterministic LTT	Equation (15) in supplementary S.1.2, SupplementaryMaterial online	Yes

Note.—A parameter is called congruence-invariant if it is identical across congruent BDS scenarios (and thus asymptotically identifiable). Noncongruence-invariant parameters cannot possibly be estimated from phylogenies alone (no matter how large) in the absence of strong additional constraints. Note that each parameter may be time-dependent, and that *τ* denotes age (time before present). Definitions are provided for nonstandard BDS parameters. Birth and death rates refer to pathogen lineages, not the hosts.

Several questions follow from this: For any given BDS scenario, 1) how can one easily determine if another given scenario is congruent?; 2) how many congruent scenarios are there?; and 3) how different can the epidemiological implications of these congruent scenarios be? To answer these questions, it is useful to consider a number of alternative model parameters, the first of which is called the pulled diversification rate and defined as:

(1) $$\tilde{r}=\lambda -\mu -\psi +\frac{1}{\lambda }\frac{d\lambda }{d\tau },$$

where *τ* denotes age or time before present (table 1). The pulled diversification rate is equal to the net diversification rate r=λ−μ−ψ (or net exponential growth rate in the case of an infectious disease) when the birth rate *λ* is constant over time, but differs from *r* otherwise. As we prove in supplementary S.1.3, Supplementary Material online, two BDS scenarios are congruent if and only if they have the same pulled diversification rate $\tilde{r}$ and the same pulled birth rate $\tilde{\lambda }$. Hence, to check if two BDS scenarios are congruent one can simply compare their $\tilde{r}$ and $\tilde{\lambda }$. Equivalently, two BDS scenarios are congruent if and only if they exhibit the same deterministic branching density $\tilde{\beta }$ and the same deterministic sampling density $\tilde{\sigma }$, which can be interpreted as rescaled probability densities over time of any randomly chosen observed branching event or sampling event, respectively, in the limit of infinitely large trees (precise definitions in supplementary S.1.2, Supplementary Material online). Hence, the shapes of $\tilde{\beta }$ and $\tilde{\sigma }$ contain easily interpretable information about the temporal distribution of branching and sampling events in the tree. Similarly to $\tilde{\lambda }$ and $\tilde{\psi }$, or $\tilde{\lambda }$ and $\tilde{r}$, so $\tilde{\beta }$ and $\tilde{\sigma }$ constitute an alternative parameterization of congruence classes, however, we stress that all of these parameters do not contain sufficient information for recovering the original model parameters *λ*, *μ*, and *ψ*.

**Fig. 1.**
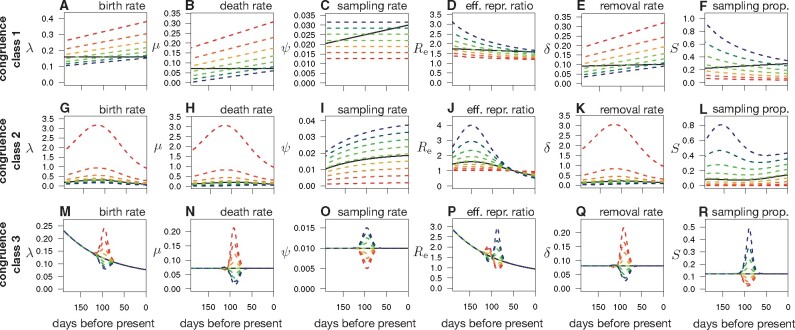
Examples of congruent epidemiological scenarios. (*A–F*) Birth (or speciation) rate (*A*), death (or extinction) rate (*B*), sampling rate (*C*), effective reproduction ratio $R_{e}=\lambda /(\mu +\psi )$ (*D*), removal rate δ=μ+ψ (*E*), and sampling proportion S=ψ/(μ+ψ) (*F*) of a specific epidemiological scenario (thick black curves), compared with various alternative congruent (i.e., statistically indistinguishable) scenarios (dashed curves). Similarly, colored curves across subfigures* A–F* correspond to a specific diversification scenario. No viral phylogeny, no matter how large, could possibly distinguish between these (and in fact a myriad of other) scenarios. (*G–L*) Similar to *A–F*, but showing scenarios congruent to a different reference scenario. (*M–R*) Similar to *A–F*, but showing scenarios congruent to a different reference scenario.

Having introduced the above new parameters, it becomes easy to answer questions (2) and (3). For any given scenario (λ,μ,ψ) and any given alternative death rate $\mu^{*}$, one can find a corresponding $\lambda^{*}$ and $\psi^{*}$ such that the new scenario ($\lambda^{*},\mu^{*},\psi^{*})$ has the same pulled diversification rate and the same pulled birth rate, that is, such that the new scenario is congruent to the first one (supplementary S.1.5.2, Supplementary Material online). Indeed, one just needs to solve the differential equation:

(2) $$\frac{d\lambda^{*}}{d\tau }=\lambda^{*}\cdot \left(\tilde{r}+\mu^{*}-\lambda^{*}\right)+\lambda \psi$$

with any initial condition, and choose $\psi^{*}=\lambda \psi /\lambda^{*}$. Many other analogous ways exist for creating congruent scenarios: For example, one can first specify an arbitrary sampling rate $\psi^{*}$ and then adjust $\lambda^{*}$ and $\mu^{*}$ accordingly, or first specify an arbitrary birth rate $\lambda^{*}$ and then adjust $\psi^{*}$ and $\mu^{*}$ accordingly, or first specify an arbitrary effective reproduction ratio $R_{e}^{*}$ and adjust $\lambda^{*},\;\mu^{*}$, and $\psi^{*}$ (details in supplementary S.1.5, Supplementary Material online). We note that some scenarios in a congruence class may have negative birth, death, or sampling rates and are therefore biologically irrelevant (Louca and Pennell 2021). Since $\mu^{*}$ (or $\psi^{*}$ or $\lambda^{*}$ or $R_{e}^{*}$) can be chosen nearly arbitrarily and can depend on an arbitrary number of free parameters, the space of congruent BDS scenarios is infinitely large and infinite-dimensional. Many congruent scenarios can appear similarly plausible and similarly complex, and yet exhibit markedly different features, including very different values and opposite trends in *λ*, *μ*, *ψ*, or $R_{e}$ (examples in fig. 1).

**Fig. 2.**
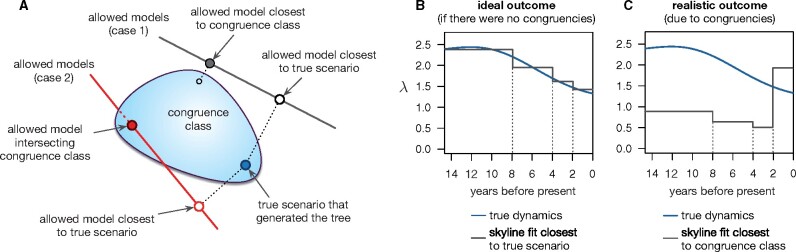
Conceptual illustration of the effects of model congruencies on epidemiological reconstruction. (*A*) The large light-blue “balloon” represents the congruence class of a single true epidemiological scenario (dark-blue circle), in the space of all biologically plausible epidemiological scenarios. Each straight continuous line represents a limited set of models or functional forms (e.g., skyline) fitted to data generated by the true scenario, for example via maximum likelihood, in an effort of approximately reconstructing the true scenario. The specific member (i.e., with a specific parameterization) chosen among each model set will be the one closest to the congruence class (filled gray circle) or may even intersect the congruence class (filled red circle), but will not necessarily be the one closest to the true scenario (open circles). This issue persists even for infinitely large data sets. (*B* and *C*) Hypothetical illustration of a skyline model (grey curve) fitted to data generated by a hypothetical scenario (blue curve, here only showing *λ*). Although no practical model perfectly matches reality, in the absence of model congruencies one would nevertheless ideally expect to obtain a fit approximately resembling reality, roughly as shown in *B*. Instead, due to model congruencies, one can easily obtain a fit that very poorly resembles the true scenario, as in *C*, since the fit closest to the congruence class is not necessarily the fit closest to the true scenario. See figure 4 for a real example.

This ambiguity limits the identifiability of epidemiological scenarios when based solely on phylogenetic data, even for infinitely large data sets. Indeed, whatever the true epidemiological history was, there will always exist an infinite number of congruent epidemiological histories. When fitting specific functional forms for *λ*, *μ*, and *ψ*, such as piecewise constant profiles known as skyline models (Stadler et al. 2013), estimators will generally converge to the scenario closest to the congruence class of the true epidemiological history, but not necessarily to the scenario closest to the true epidemiological history itself (fig. 2). Depending on the particular functional forms chosen for *λ*, *μ*, and *ψ*, this can yield markedly different rate profiles over time that may not even approximately resemble the true epidemiological history, including wrong trends and major spurious features (e.g., fig. 2*C*). This can occur even if the fitted functional forms are flexible enough to reasonably approximate the true epidemiological history (e.g., fig. 2*B*). In other words, even if one can closely identify the congruence class that best explains the data (e.g., in terms of the $\tilde{\lambda }$ and $\tilde{\psi }$, or in terms of the $\tilde{\beta }$ and $\tilde{\sigma }$), without additional constraints or information one cannot determine which member of the congruence class generated the data. We emphasize that it is not necessary for multiple congruent scenarios to exist within the class of fitted functional forms in order to run into these issues. Further, since the scenarios in a congruent class are indistinguishable for any data set of any size, with some generally appearing simpler and others more complex than the true epidemiological history, model selection techniques based on parsimony that do not incorporate additional information (e.g., from independent studies) cannot alone resolve this issue (detailed discussion in supplementary S.2, Supplementary Material online). In a Bayesian context, the existence of vastly different congruent scenarios means that the uncertainty in the estimated *λ*, *μ*, and *ψ* is even more sensitive to the choice of priors than would be apparent from comparing the posterior to the prior distributions of the parameters of some fitted model class. These issues are also expected to affect hypothesis testing approaches that rely on model fitting, for example, fitting a linear profile for *λ* and examining whether the fitted slope is statistically significant to see if *λ* has been changing in a specific direction. Following the above arguments, the fitted slope need not at all reflect the true trend in *λ* (e.g., it could point in the opposite direction), and for a sufficiently large data set this wrong slope will inevitably be statistically significant. The above issues also apply to other equivalent model parameterizations used in epidemiology, for example based on $R_{e}$, the sampling proportion (S=ψ/(μ+ψ)) and the removal rate (δ=μ+ψ, also known as “become uninfectious rate”) (Stadler et al. 2013). Note that, in contrast, $\tilde{\lambda },\;\tilde{\psi },\;\tilde{r},\;\tilde{\beta }$, and $\tilde{\sigma }$ are asymptotically estimateable, that is, given sufficiently large trees (supplementary S.1.2, Supplementary Material online).

It was previously demonstrated that for constant-rate scenarios (i.e., where the parameters *λ*, *μ*, and *ψ* do not vary with time), it is impossible to simultaneously estimate *λ*, *μ*, and *ψ* from timetrees alone, because alternative combinations of constant *λ*, *μ*, and *ψ* yield the same likelihood function (Stadler et al. 2013; Gavryushkina et al. 2014). It is also known that when fitting BDS skyline models, at least one of the parameters must be fixed in at least one of the time intervals to eliminate correlations between parameter estimates (Stadler et al. 2013). Our findings are a generalization of these special cases and reveal that these underestimate the true extent of the issue. As soon as one considers that epidemiological parameters could vary over time in an unknown fashion (Grassly and Fraser 2008; Nishiura and Chowell 2009; Cowling et al. 2010), even if they can in principle be approximated by the considered model type (e.g., fig. 2*B*), much more information is needed to “collapse” the congruence class and accurately reconstruct the true epidemiological dynamics. In particular, fitting generic BDS skyline models to real molecular data without constraints (or only constraining one parameter in one time interval), as is common practice (Stadler et al. 2013; Kühnert et al. 2016; Resende et al. 2021), cannot be expected to yield an accurate reconstruction, no matter how large the data set.

The above results are analogous to the macroevolutionary case, where the pulled birth rate is asymptotically identifiable, but does not contain sufficient information for recovering the true birth and death rates (Louca and Pennell 2020). Further, as in the macroevolutionary case, so here two congruent BDS scenarios have identical deterministic lineages-through-time (dLTT) curves (i.e., the number of lineages one would expect in the tree over time based on a deterministic interpretation of the rates *λ*, *μ*, and *ψ*) when conditioned on the number of lineages at some given time point. In contrast to the macroevolutionary case, however, the reverse needs no longer be true, that is, two BDS scenarios with identical dLTTs need not necessarily be congruent. Moreover, although in the macroevolutionary case a congruence class could be uniquely described by a single time-dependent parameter (e.g., the dLTT, or the pulled birth rate over time), here a BDS congruence class is determined by two time-dependent parameters (e.g., the pulled birth rate and pulled diversification rate).

## Simulation Examples

To demonstrate the challenges for epidemiological inference stemming from the existence of model congruencies, we simulated various hypothetical but realistic epidemiological scenarios and then used two alternative well-established approaches for reconstructing the original dynamics from the generated data. In the first approach, we used the true timetree as an input and estimated the epidemiological dynamics via maximum-likelihood fitting. In the second approach, we used nucleotide sequences simulated along the timetree as input, and jointly estimated the timetree together with the epidemiological dynamics using Bayesian Markov chain Monte Carlo (MCMC). The latter approach resembles the common situation in molecular epidemiology where the phylogeny is not a priori known, thus introducing additional uncertainty in the reconstruction of the epidemic’s dynamics. To avoid introducing our own biases, for example in the choice of priors, the Bayesian analysis was performed in a blinded way, with some members of our team conducting the simulations and others conducting the Bayesian inference.

**Fig. 3.**
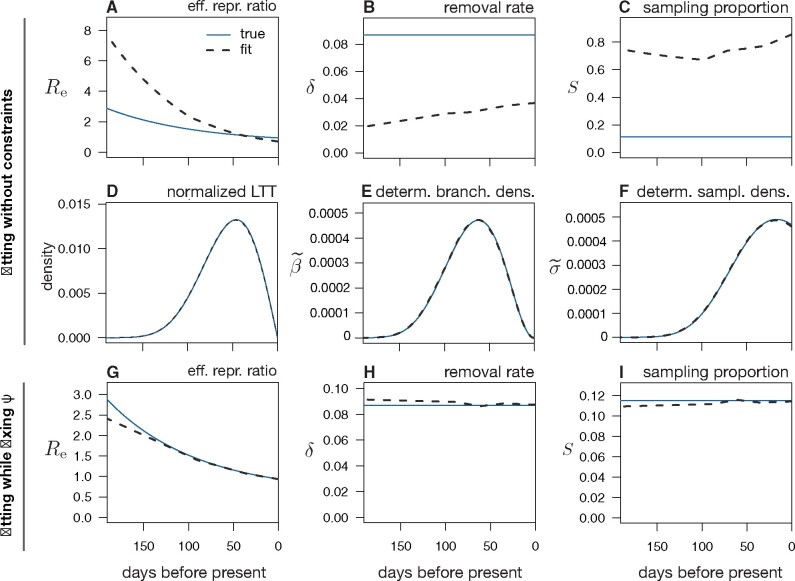
Limits to reconstructing an epidemic’s dynamics via maximum likelihood. (*A–C*) Maximum-likelihood estimates (grey dashed curves) of the effective reproduction ratio ($R_{e}$), removal rate (δ=μ+ψ), and sampling proportion (S=ψ/(μ+ψ)) over time, based on a timetree with 175,440 tips simulated under a hypothetical BDS scenario (blue continuous curves). Rates are in $day^{-1}$. Model adequacy was confirmed via parametric bootstrapping with multiple test statistics. Observe the poor agreement between the estimated and true profiles. (*D–F*) Maximum-likelihood estimates of the dLTT curve (normalized to have unit area under the curve), deterministic branching density ($\tilde{\beta }$), and deterministic sampling density ($\tilde{\sigma }$), corresponding to the same fitted model as in A–C, compared with their true profiles. The good agreement between the inferred and true profiles shows that the fitted model converged toward the true epidemiological scenario’s congruence class but not the true scenario itself. (*G–I*) Maximum-likelihood estimates of $R_{e}$, *δ*, and *S* inferred from the same data as in *A–F*, while fixing the sampling rate *ψ* to its true profile. For additional BDS parameters see supplementary figure S1, Supplementary Material online. For a statistical analysis of estimation accuracies across trees simulated from many random scenarios, see supplementary figures S17 and S19, Supplementary Material online.

For the maximum-likelihood inference, we simulated timetrees with >50,000 tips and fitted generic piecewise-linear profiles for *λ*, *μ*, and *ψ* to each timetree, while selecting the optimal number of inflection points using AIC. We used such massive timetrees to avoid errors stemming from small sample sizes, thus focusing on identifiability issues. The fitted models matched the LTTs of the timetrees and the deterministic LTTs of the true scenarios nearly perfectly (fig. 3*D* and supplementary fig. S1*G*, Supplementary Material online). The fitted models also adequately explained the timetrees based on three different statistical tests performed via parametric bootstrapping (Brown and Thomson 2018) (Kolmogorov–Smirnov tests on the distributions of node ages, tip ages, and edge lengths, *P *>* *0.05 in all cases). Despite the large sizes of the data sets and the adequacy of the models in explaining the data, the corresponding estimated *λ*, *μ*, *ψ*, $R_{e}$, *δ*, and *S* were nearly always very different from the true profiles used in the simulations, sometimes even exhibiting opposite trends (fig. 3*A–C* and supplementary figs. S1*A–F* and S2*A–F*, Supplementary Material online). Importantly, the fitted models nearly exactly reproduced the deterministic branching density $\tilde{\beta }$ and deterministic sampling density $\tilde{\sigma }$ of the true scenario ($R^{2}>0.99$ in all cases), which, as explained earlier, implies that the fitted models came very close to the true scenario’s congruence class (figs. 3*E* and *F*). This confirms our expectation that fitting yields an estimate of the true epidemiological history’s congruence class, but not necessarily of the true epidemiological history itself. These issues are expected to be even more pronounced with the smaller data sets typical in epidemiology, due to elevated stochasticity.

For the Bayesian inference, we considered two alternative epidemiological scenarios to simulate timetrees of sizes typical in molecular epidemiology (590 and 540 tips, spanning about 15 years, supplementary fig. S3, Supplementary Material online). The epidemiological scenarios and the nucleotide substitution models exhibited parameters typically reported for HIV-1 (Leitner et al. 1997; Posada and Crandall 2001; Duchêne et al. 2015). The profiles of *λ*, *μ*, *ψ*, $R_{e}$, *δ*, and *S* exhibited moderate variation over time that could be well-approximated using skyline models with three to four intervals. For each of the two scenarios, we then conducted a BDS skyline model inference in BEAST2 (Bouckaert et al. 2019) using the sampled sequences as input data. As indicated above, this inference was internally blinded, that is, the team members performing the inference had no knowledge of the true epidemiological scenarios used in the simulations. The sole information provided was 1) that model parameters were within the typical ranges known from HIV-1, 2) that four time intervals are sufficient for reasonably approximating the true dynamics with a skyline model (thus avoiding complications in the selection of the number of intervals), 3) the nucleotide substitution model and number of rate categories used, and 4) the value of the present-day sampling proportion, to account for known identifiability issues within skyline models (one parameter in one time interval must be fixed to eliminate correlations between parameters according to Stadler et al. [2013]). Each of the parameters $R_{e}$, *δ*, and *S* varied over time with rate shifts 2, 4, and 8 years before the present, and the sampling proportion in the present was fixed to its known value. The adequacy of the posterior models to explaining the true tree was fully confirmed via predictive posterior simulations (Brown and Thomson 2018) based on the same statistical tests as used for the maximum-likelihood fits. The molecular clock rate and the parameters of the nucleotide substitution model were also largely accurately estimated (supplementary figs. S10 and S12, Supplementary Material online).

**Fig. 4.**
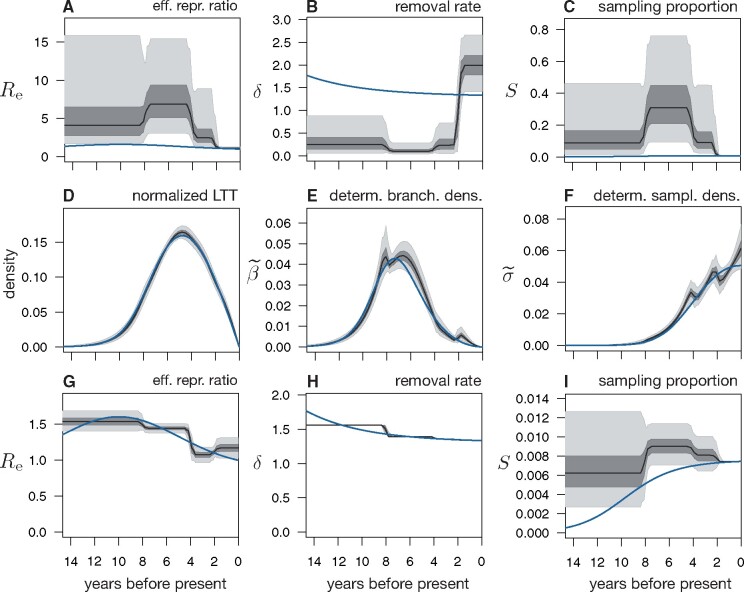
Limits to reconstructing an epidemic’s dynamics in a Bayesian framework. (*A–C*) Posterior distributions of the effective reproduction ratio ($R_{e}=\lambda /(\mu +\psi )$), removal rate (δ=μ+ψ), and sampling proportion (S=ψ/(μ+ψ)), as inferred from 590 sequences simulated under a hypothetical BDS scenario (blue curves) using BEAST2. Black curves show posterior median, dark and light shades represent equal-tailed 50%- and 95%-credible intervals of the posterior. All rates are in $yr^{-1}$. The present-day sampling proportion was fixed to its true value during fitting to account for previously reported identifiability issues in skyline models (Stadler et al. 2013). Model adequacy was confirmed using predictive posterior simulations with multiple test statistics. Observe the poor agreement between the posterior predictions and the true profiles. For additional epidemiological parameters (*λ*, *μ*, *ψ*), see supplementary figure S5, Supplementary Material online. For the molecular evolution parameters, see supplementary figure S10, Supplementary Material online. (*D–F*) Distributions of the dLTT curves (normalized to have unit area under the curve), deterministic branching densities ($\tilde{\beta }$), and deterministic sampling densities ($\tilde{\sigma }$), corresponding to the same posterior models as in *A–C*, compared with their true profiles (blue curves). The relatively good agreement between the inferred and true profiles shows that BEAST2 closely reconstructed the true epidemiological history’s congruence class, but not the true epidemiological history itself. (*G–I*) Posterior distributions of $R_{e}$, *δ*, and *S* inferred from the same data, while fixing the present-day sampling proportion and the removal rate’s profile to their true values. For additional parameters, see supplementary figures S6 and S11, Supplementary Material online.

In contrast, in both scenarios, the estimated *λ*, *μ*, *ψ*, $R_{e}$, *δ*, and *S* deviated substantially from their true profiles, in many cases exhibiting different trends over time or differing by more than an order of magnitude (e.g., when considering the maximum-posterior or median-posterior profiles, fig. 4*A–C* and supplementary figs. S5 and S7, Supplementary Material online), consistent with our expectations. Moreover, the posterior 95% equal-tailed credible intervals barely overlapped with the true profiles of these parameters, suggesting that the inferred posterior distributions severely underestimate the true uncertainty of the parameters. This is not surprising, since these posteriors only reflect the uncertainty stemming from finite data sizes, but do not account for congruencies. Importantly, in all cases, the posterior distributions of the deterministic branching and sampling densities ($\tilde{\beta }$ and $\tilde{\sigma }$) predicted by BEAST2 closely resembled their true profiles (fig. 4*D–F*). This implies that BEAST2 accurately inferred the congruence class of the true epidemiological history, just not the true epidemiological history itself (again, consistent with our predictions). To further test this interpretation, we repeated the Bayesian inference while fixing the removal rate to its true profile (approximated by a piecewise constant curve for compatibility with the skyline model). In both scenarios, the inferred BDS model parameters much more closely resembled their true profiles (supplementary figs. S6 and S8, Supplementary Material online), consistent with the fact that fixing the removal rate profile and the present-day sampling proportion collapses the congruence class to a single scenario (mathematical proof in supplementary S.1.5.7, Supplementary Material online). Together, these results confirm our expectations that any tool attempting to reconstruct an epidemic’s dynamics based on phylogenetic data alone and without strong additional constraints, no matter how large the data set, can only reconstruct the congruence class of those dynamics rather than the true dynamics.

To further confirm that the above issues are likely to occur in practice, we performed multiple simulations of random epidemiological scenarios (i.e., with *λ*, *μ*, and *ψ* having randomly constructed but plausible profiles over time) and examined the accuracy of maximum-likelihood fitted BDS models with generic piecewise-linear profiles (the grid size was chosen according to AIC [Akaike 1981]). Trees comprised between 500 and 50,000 tips, and we only considered cases where the fitted model adequately described the data, based on the same Kolmogorov–Smirnov tests as above (details in supplementary S.3, Supplementary Material online). In the vast majority of cases, and even for the largest trees, the estimated parameters *λ*, *μ*, *ψ*, $R_{e}$, *S*, and *δ* poorly reflected the true profiles used in the simulations, often exhibiting opposite long-term trends and spurious major features, and sometimes even deviated by orders of magnitude from the truth (overview in supplementary figs. S17 and S19, Supplementary Material online, examples in supplementary figs. S18 and S20, Supplementary Material online). For example, the coefficient of determination (*R*^2^) between the true $R_{e}$ and the fitted $R_{e}$ was almost always below 0 (supplementary figs. S17*A* and S19*A*, Supplementary Material online). In contrast, the deterministic branching and sampling densities ($\tilde{\beta }$ and $\tilde{\sigma }$) were generally accurately estimated (typical $R^{2}>0.9$, supplementary figs. S17*D*, 17*E* and S19*D*, 19*E*, Supplementary Material online). Similarly, the mean error relative to the average was about an order of magnitude lower for $\tilde{\beta }$ and $\tilde{\sigma }$ (average ≈7%) than for $R_{e}$ (average ≈80%). These findings show that most fitted models did converge toward the true congruence classes, but not to the actual true scenarios.

## Illustration for an HIV Epidemic

**Fig. 5.**
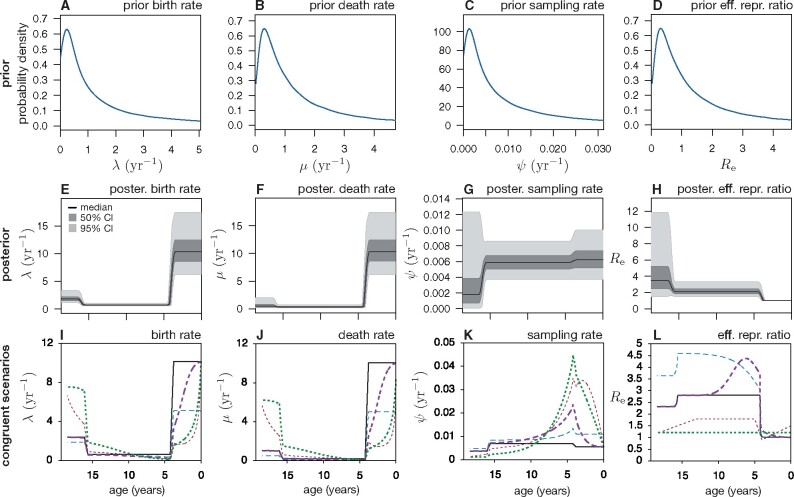
Bayesian reconstruction of HIV spread is compromised by model congruencies. (*A–D*) Specified priors for BDS (skyline) model parameters of HIV-1 subtype B in Northern Alberta, reflecting our a priori knowledge of the plausible range of these parameters. (*E–H*) Distribution of BDS parameters over time, based on models sampled from the posterior distribution by BEAST2. At each time point, the black curve shows the median value of a parameter across all posterior-sampled models, whereas the dark and light shadings show 50% and 95% equal-tailed highest posterior density intervals, respectively. (*I–L*) Maximum posterior probability BDS “reference” scenario (continuous black curves) compared with multiple alternative “congruent” scenarios (dashed curves). Each congruent scenario would generate timetrees with the same probability distribution as the reference scenario and is thus statistically indistinguishable from the latter. For the posterior distributions of molecular evolution parameters, see supplementary figure S23, Supplementary Material online.

To further illustrate the practical implications of model congruencies, we considered the dynamics of HIV-1 subtype B in Northern Alberta, Canada, over the course of roughly 20 years, reconstructed from 563 molecular sequences using Bayesian BDS skyline models in BEAST2 (Stadler et al. 2013; Bouckaert et al. 2019). We assumed that $R_{e}$, *δ*, and *S* varied over time and shifted in 1998 (when triple antiretroviral therapy became available) and in 2010 (to achieve a roughly balanced partitioning of sampling dates). Rate priors were chosen conservatively to reflect the general uncertainty across HIV outbreaks (see Materials and Methods) and an uncorrelated log normal relaxed clock was supported over a strict clock based on nested sampling (Russel et al. 2019). We found that the posterior distribution of BDS models (fig. 5*E–H*) strongly suggests a decline of $R_{e}$ over time, a stabilization of the sampling rate in the last two intervals, and a dramatic increase in transmission and recovery rates when comparing the first to the last time interval. The narrow posterior 95% equal-tailed credible intervals for $R_{e}$ in the second and third time intervals (fig. 5*H*) suggest that $R_{e}$ is well-constrained.

However, this posterior is misleading because the inferred credible intervals do not properly capture the ambiguities stemming from model congruencies. Indeed, recall that what we are really estimating is the congruence class of the true epidemiological history, and not the true epidemiological history itself. For illustration, consider the sampled model with maximum posterior probability shown in figure 5*I–L*. This representative scenario is congruent to a myriad of alternative and markedly different epidemiological scenarios, many of which are similarly complex and a priori similarly plausible (examples in fig. 5*I–L*). All of these alternative scenarios are equally likely to have generated the data at hand, and this would be true for any other phylogenetic data set as well. Ruling out some of these congruent scenarios in favor of others requires additional knowledge, such as strong priors on the parameters. Hence, the true uncertainty in the inferred epidemiological parameters is largely determined by the imposed priors, rather than by the computed posterior densities. However, many of the congruent scenarios are not in strong contrast to our priors (examples in fig. 5*A–D*), which are typical in the epidemiological literature. In other words, much stronger priors would be needed to collapse the congruence class down to a practical size (e.g., suitably precise for policy decisions), even with massive phylogenetic data sets.

## Ways Forward

In our recent analysis of macroevolutionary BD models (Louca and Pennell 2020), we proposed that researchers could develop methods to draw insight from asymptotically identifiable variables (i.e., those which are identical between congruent scenarios); in the epidemiological case such quantities include $\tilde{\lambda },\;\tilde{\psi },\;\tilde{r},\;\tilde{\beta }$, and $\tilde{\sigma }$. Indeed, for the macroevolutionary case, such identifiable variables do contain useful information about historical diversification dynamics (Louca et al. 2018). A similar strategy could potentially be fruitful for epidemiological data, although we do not further explore that possibility here. Instead, in the following, we discuss a number of ways, some of which build upon current practices in the field, to robustly reconstruct typical epidemiological variables of interest such as $R_{e}$.

First, following on what is sometimes done in practice, one can use additional clinical or surveillance data to constrain specific epidemiological parameters. Although it is generally recognized that parameter estimation benefits from the use of available constraints, the precise effects of constraints in phylodynamics remained poorly understood and their importance severely underestimated. Our results precisely clarify the amount of information necessary to make an epidemiological scenario asymptotically identifiable. For example, if one of *λ* or *ψ* is known beforehand, then the remaining variables become asymptotically identifiable (details in supplementary S.1.5, Supplementary Material online). This is because in any congruence class there exists at most one scenario with a specific *λ* or *ψ*. Indeed, when we fixed the sampling rate *ψ* to its true profile in our earlier maximum-likelihood fitting tests, the fitted BDS models closely reproduced the true scenarios (supplementary figs. S1*H–N* and S2*H–N*, Supplementary Material online). Similarly, if *μ* is somehow independently known and either *λ* or *ψ* is known on at least one time point, then the full scenario again becomes asymptotically identifiable. Similar arguments can be made for $R_{e}$, *δ*, or *S* (supplementary S.1.5.5, S.1.5.6, and S.1.5.8, Supplementary Material online). Such constraints might be obtained in a variety of ways. For example, in some situations, it might be assumed that nearly all people are diagnosed and sampled, in which case the sampling proportion might be fixed to 1. Alternatively, one may estimate the true prevalence of a disease through occasional serological surveys of randomly chosen individuals (Farrington and Whitaker 2003; Lai, Wang, et al. 2020) and then divide the “background rate” of disease detection by that estimate to obtain *ψ*. Further, clinical data may be used to estimate the rate of host death or recovery (i.e., *μ*). During maximum-likelihood fitting, one can fix the independently known parameters. In a Bayesian framework, one can impose appropriate data-driven priors, that is, based on independent information, to constrain the known parameters. However, we stress that in order to eliminate most congruencies and accurately reconstruct the remaining parameters, these priors will need to be much more restrictive than in typical studies (Nadeau et al. 2021). For example, in the second simulation of our Bayesian inference tests described earlier the true removal rate was nearly constant; when we used this information to constrain the removal rate in BEAST2 (i.e., demanding that it is constant across all time intervals), the parameters *λ*, *μ*, *ψ*, $R_{e}$, *δ*, and *S* were estimated much more accurately than in the absence of this constraint (supplementary fig. S9, Supplementary Material online). Their accuracy improved further when we fixed the removal rate to its true profile (supplementary fig. S8, Supplementary Material online). Although constraints such as the above are sometimes included in molecular epidemiological studies, many studies still attempt to estimate the full epidemiological dynamics (*λ*, *μ*, and *ψ*) solely from phylogenies (Stadler et al. 2013; Paraskevis et al. 2015; Lai, Bergna, et al. 2020). In contrast, multiple sources of information are commonly utilized when fitting mechanistic epidemiological models, such as differential equation models, or in nonparametric analyses of surveillance data over time. For example, it is common for surveillance data to be combined with independently determined infectivity profiles (Cori et al. 2013), which are essentially a generalization of *λ*, or with estimates of the serial interval (Najafi et al. 2020), to estimate $R_{e}$ over time. Our mathematical results clarify that using additional information beyond just molecular data, for example, from clinical trials or serological surveys, is not just a means to increase the number of data points—it is essential for ensuring the identifiability of temporally variable epidemiological histories. Developing new phylodynamic models that readily integrate appropriate additional data sources would greatly facilitate this practice (Gupta et al. 2020; Manceau et al. 2021) (but see supplementary S.5, Supplementary Material online for limitations on the use of occurrence data).

Second, although molecular epidemiological studies are typically purely observational—that is, the sampling and data analysis are done independently—this does not need to be the case. Indeed, a properly designed sampling scheme can help reduce identifiability issues. Concretely, short, high-intensity “concentrated” or “contemporaneous” sampling attempts (CSAs), where many individuals are randomly sampled (in addition to sampling symptomatic individuals), can yield valuable information for reconstructing the dynamics of an epidemic and for partly resolving congruencies (details in supplementary S.1.6 and S.1.7, Supplementary Material online). The main requirements are that, first, these CSAs are much shorter than the current expected birth and death times (i.e., much shorter than 1/λ and 1/μ), second, *λ* does not differ substantially between the beginning and end of the CSA, and third, the number of lineages sampled during the CSA is much greater than the number of birth or death events occurring in that time period. Such a sampling strategy is not just a hypothetical possibility: during 2020, multiple governments reportedly conducted CSAs to estimate the seroprevalence of SARS-CoV-2 (Menachemi et al. 2020; Pollán et al. 2020). If such sampling attempts are performed repeatedly over time and at sufficient temporal resolution, and/or they are combined with other local sequencing data, they can enable an accurate reconstruction of *λ*, *ψ*, and consequently *μ* over time. For example, when we resimulated the two hypothetical BDS scenarios used earlier for maximum-likelihood estimation while including 3 CSAs, the subsequently refitted models reproduced the true BDS scenarios much more accurately (supplementary figs. S1*O–U* and S2*O–U*, Supplementary Material online). Note that CSAs differ from the common approach of testing individuals only upon the appearance of symptoms, as in these cases the number of infections detected during any given period tends to be smaller than the number of new infections occurring during that period. The importance of optimal sampling design to improve identifiability has previously been recognized for coalescent models in epidemiology (Stack et al. 2010; Parag and Pybus 2019). Notably, Stack et al. (2010) concluded that sampling sequences at specific time points tend to improve inferential power compared with less focused sampling protocols, resembling our conclusions above for BDS models.

A third potential approach could be to only fit profiles for *λ*, *μ*, and *ψ* with a strong mechanistic justification, that is derived from models for infectious disease dynamics (Kühnert et al. 2014; Rasmussen et al. 2014; Vaughan et al. 2019; MacPherson et al. 2021), rather than generic profiles (e.g., skyline models). Notably, BD SIR and BD SIS models (Kühnert et al. 2014; Leventhal et al., 2014; Vaughan et al. 2019) are increasingly used and a good start in this direction, although for longer time periods or spatially structured epidemics more complex models will generally be needed. Whether this approach is effective in avoiding the issues stemming from congruencies in practice is unknown and warrants future investigation. If the true epidemiological history was indeed perfectly described by a given mechanistic model, then fitting that model to a timetree will probably yield accurate parameter estimates, provided of course a sufficiently large data set. However, nature rarely exactly follows our mechanistic models, and this is certainly true for epidemics. In this situation, as when fitting generic profiles (e.g., skyline models), fitting a mechanistic model will generally merely yield a scenario close to the congruence class of the true epidemiological history; in the case of a complex mechanistic model exhibiting a variety of qualitatively different behaviors (depending on parameter choice), this might yield a scenario far from the true history itself, even for large data sets (see conceptual fig. 2 and earlier discussion).

We stress that coalescent analysis, an alternative popular framework for reconstructing an epidemic’s effective size ($N_{e}$) over time based on pathogen sequences (Lambert and Stadler 2013; Hill and Baele 2019), cannot resolve the issues discussed here. Although $N_{e}$ is in theory asymptotically identifiable provided sufficiently large trees, provided that all assumptions of coalescent theory are satisfied and provided that the generation time *T* (or serial interval, which is analogous to 1/λ) are independently known, the issue of identifiability is essentially being replaced by strong and questionable assumptions about the sampling and transmission process (Boskova et al. 2014). For example, using solely phylogenetic data coalescent theory can a priori only yield information on the product $N_{e}T$, and additional assumptions or independent information are needed about *T* in order to actually obtain $N_{e}$ and $R_{e}$ (Hall et al. 2016). In the general situation where *λ* (and hence *T*) can vary arbitrarily through time and is unknown, one is thus faced with similar identifiability issues as with BDS models.

## Conclusions

Our results highlight the limitations of epidemiological inference using phylogenetic data alone. The reported identifiability issues are particularly serious for cases where a reconstruction of historical dynamics is attempted based solely on phylogenetic data and without any additional strong constraints. In such situations, it is generally impossible to reliably reconstruct key epidemiological variables, such as $R_{e}$, over time, no matter how large the data. We stress that these issues are separate from the well-recognized errors due to small data sets (Rasmussen et al. 2014), since any two congruent BDS scenarios remain statistically indistinguishable even for infinitely large phylogenies and hence large phylogenetic data sets alone cannot possibly resolve these issues. Instead, additional data sources beyond just phylogenies, such as from clinical experiments or seroprevalence surveys, are necessary for accurate reconstruction. On a more positive note, we have fully resolved the informational connections between major epidemiological parameters and provide tools for determining their identifiability based on phylogenetic data. In particular, we provide code for exploring the full extent of congruent BDS scenarios and for assessing which epidemiological scenarios can in principle (i.e., with a sufficiently large data set) be distinguished from one another (supplementary S.4, Supplementary Material online). Our results can help guide proper experimental and sampling designs optimized for epidemiological reconstruction.

## Materials and Methods

### Simulations for Maximum-Likelihood Inferences

To demonstrate the implications of model congruencies using simulated trees, we proceeded as follows. Timetrees were generated under two alternative BDS scenarios using the function generate_tree_hbds in the R package castor v1.6.7 (Louca and Doebeli 2018) (with options “include_extant=FALSE, include_extinct=FALSE, no_full_extinction=TRUE”). In the first scenario, *μ* and *ψ* were assumed to be constant over time whereas $R_{e}$ was exponentially decreasing over time (profiles in supplementary fig. S1*A–F*, Supplementary Material online) and the simulation was halted after 200 days, resulting in a tree with 175,440 tips. In the second scenario, *λ* and *μ* were constant over time whereas *ψ* increased continuously toward the present (profiles in supplementary fig. S2*A–F*, Supplementary Material online) and the simulation was halted after 200 days, resulting in a tree with 55,934 tips. To each tree, we fitted BDS models with a priori unknown *λ*, *μ*, and *ψ*, each defined as a piecewise-linear function of time with inflections at fixed time points (chosen such that their density is approximately proportional to the square root of the tree’s LTT). The optimal number of time-grid points (i.e., the number of fitted parameters for each of *λ*, *μ*, and *ψ*) was chosen by minimizing the AIC of the fitted model (Akaike 1981). For any given grid size, fitting was done via maximum-likelihood using the castor function fit_hbds_model_on_grid, with options “condition = ‘auto,’ max_start_attempts = 100, Ntrials = 100.” Additional epidemiological variables of the fitted models (such as $R_{e}$ and the LTT) were computed using the castor function simulate_deterministic_hbds. To demonstrate the effects of fixing *ψ* to its true value during fitting, we repeated the above fitting process while fixing *ψ* to its true time profile. To demonstrate the effects of CSAs, discussed in the main article, we also simulated trees under BDS scenarios similar to the above but modified to include CSAs at three discrete time points. We then used these new trees to fit BDS models with unknown *λ*, *μ*, and *ψ*, defined as piecewise-linear functions, while also accounting for the added CSAs in the computation of the likelihood (MacPherson et al. 2021). As before, fitting was performed via maximum-likelihood and by choosing the time-grid size according to the AIC. During fitting, the times and intensities (i.e., sampling probabilities) of the CSAs were fixed to their true values (through options “CSA_ages” and “fixed_CSA_probs”), reflecting general-population randomized seroprevalence surveys in which these properties can be independently determined. The fitted models are shown in supplementary figures S1 and S2, Supplementary Material online. We mention that throughout this article “age” refers to time before present, and “present” refers to the time at which the sampling process was halted.

### Simulations for Bayesian Inference

To explore the ability of Bayesian MCMC sampling to recover the true epidemiological history with varying levels of constraints, we conducted an internally blinded BEAST2 BD skyline inference using sampled sequences generated from realistic HIV epidemic simulations. Specifically, group member A simulated timetrees and nucleotide sequences under two different BDS scenarios lasting approximately 15 years, resulting in trees with 590 and 540 tips, respectively (supplementary fig. S3, Supplementary Material online). The parameters *λ*, *μ*, *ψ*, $R_{e}$, *δ*, and *S* were all within reasonable ranges, that is, with values within the priors that would typically be specified for an HIV model, with moderate variation through time that can be well-approximated using a skyline model with 3–4 time intervals (supplementary figs. S5*A–F* and S7*A–F*, Supplementary Material online). Nucleotide sequences of length 1,000 bp were simulated along the timetree under an independent-sites HKY substitution model (Hasegawa et al. 1985) with transition/transversion ratio 5 (Duchêne et al. 2015) and stationary base frequencies A:0.4, C:0.17, G:0.21, T:0.22 (Posada and Crandall 2001). The root sequence was chosen randomly according to the stationary base frequencies. Nucleotides were randomly assigned to one of four strict clock substitution rate categories, whose rates were chosen according to a discretized gamma distribution as described by (Yang 1994), with shape parameter α=0.5 (Leitner et al. 1997; Posada and Crandall 2001; Duchêne et al. 2015) and mean rate $2\times 10^{-3}\;{\text{yr}}^{-1}$.

The simulated sequence alignments and their sampling dates were provided to a second team (“B”) as input data for reconstructing the epidemiological dynamics over time using serial skyline (i.e., piecewise constant) BDS models in BEAST2 v2.6.2 with BEAGLE v4.1 (Ayres et al. 2012; Stadler et al. 2013; Bouckaert et al. 2019). As mentioned earlier, team B did not have knowledge of the true epidemiological parameters and was initially only provided with the following information: the nucleotide substitution model used (HKY with independent sites), the fact that there were four rate categories according to a discretized gamma distribution, the fact that four time intervals would be sufficient for reasonably approximating the two epidemic histories, the fact that all parameters were chosen within ranges typical for HIV-1, and the present-day sampling proportion. Team B confirmed that there was adequate temporal signal in the sequence data by evaluating the distribution of pairwise patristic distances and divergence over time on a preliminary approximate maximum-likelihood tree made in FastTree2 (Price et al. 2010) and rooted using residual mean square fitting in Tempest v1.5.3 (Rambaut et al. 2016). Based on the distribution of sampling and branching dates (supplementary figs. S3*C–F* and S4, Supplementary Material online), and to ensure similar amounts of data in each interval, the rate shifts in these models were specified to occur at 2, 4, and 8 years before the present. Skyline models were parameterized in terms of $R_{e}$, removal rate *δ*, and sampling proportion *S*, each of which could vary independently in each time interval. In all cases, the present-day sampling proportion was fixed to its true value, to account for known correlations between skyline model parameters (Stadler et al. 2013). Runs were set up on two independent MCMC chains for 100–200 million states, sampled every 10,000 states (overview of priors in supplementary table S1, Supplementary Material online). For each run, log files from both chains were combined using LogCombiner (Bouckaert et al. 2019) after confirming convergence in Tracer and removing 10% burn-in. We refer to these two runs as U1 and U2 (“unconstrained” 1 and 2). For each model drawn from the posterior distribution, we calculated the deterministic LTT, the deterministic branching density, and deterministic sampling density using the castor function simulate_deterministic_hbds. Equal-tailed credible intervals of various model parameters were calculated for the posterior distribution of scenarios using the quantile function in R. To investigate how the parameter estimates would improve if one were to provide sufficient constraints to collapse the congruence class, we repeated the BEAST2 runs while fixing the removal rate over the entire time period to its true profile (approximated by a piecewise constant curve for compatibility with the skyline model) and while fixing the present-day sampling proportion to its true value (as before); we refer to these new runs as F1 and F2, respectively (“fixed” 1 and 2). Lastly, for the second scenario where the removal rate was nearly constant over time, we investigated how this information might improve parameter estimates, by constraining the removal rate to be constant across all time intervals (with unknown value); we refer to this run as C2 (“constrained” 2). Posterior distributions of epidemiological parameters from runs U1, F1, U2, F2, and C2 are shown in supplementary figures S5–S9, Supplementary Material online, respectively. The corresponding posterior distributions of molecular evolution parameters are shown in supplementary figures S10–S14, Supplementary Material online. MCMC traces of runs U1 and U2 are shown in supplementary figures S15 and S16, Supplementary Material online, respectively. The above analyses were also repeated for sequences simulated under a strict molecular clock model with a single discretized rate category from the gamma distribution, yielding similar results.

### Model Adequacy Tests

To verify that each maximum-likelihood-fitted model was adequate for explaining the timetree, that is, that parameter estimates were not due to bad model fits, we used parametric bootstrapping to compare various properties of the tree to those expected under the fitted model (Brown and Thomson 2018; Schwery 2019), as follows. For any given tree and fitted model, we simulated 1,000 random trees using the model from the root to the present-day using the function generate_tree_hbd_reverse in the R package castor (Louca 2020). We then compared the distribution of tip ages generated by the fitted model to the original tree using a Kolmogorov–Smirnov test. Specifically, for every simulated tree, we calculated the empirical cumulative distribution function (CDF) of the tip ages (denoted *F*, and evaluated at the original tree’s tip ages via linear interpolation), and then calculated the average of those CDFs, hence obtaining an estimate for the CDF of tip ages generated by the model (denoted $\bar{F}$). The Kolmogorov–Smirnov (KS) distance between a tree’s CDF *F* and $\bar{F}$, denoted $D(F,\bar{F})$, is the maximum distance between *F* and $\bar{F}$ at any age. The statistical significance (*P*) of the original tree’s KS distance $D(F_{o},\bar{F})$ was calculated as the fraction of simulated trees for which $D(F,\bar{F})$ was larger than $D(F_{o},\bar{F})$. Hence, a small *P* means that the original tree’s distribution of node ages differs substantially from that expected under the fitted model. A similar approach was followed for comparing the model’s and tree’s distribution of node ages or edge lengths. To perform analogous model adequacy tests for our Bayesian analysis, we compared the true timetree (generated during the simulation of the hypothetical epidemic) to the scenarios drawn from the posterior distribution. The methodology was nearly identical to that described above for the maximum-likelihood fits, with the only substantial difference being that each random tree was generated by a scenario randomly chosen from the posterior distribution. The above statistical tests are conveniently implemented in the castor function model_adequacy_hbds (Louca and Doebeli 2018).

### Empirical HIV Analysis

For the empirical HIV analysis, we used publicly available HIV-1 sequences from Northern Alberta, Canada, collected between 2007 and 2013 and previously described by Vrancken et al. (2017). Of the 1,055 partial pol sequences (consisting of full *protease* and the first 240 or 400 codons of *reverse transcriptase*) available on GenBank, the analysis was restricted to 809 subtype B sequences, as determined by Vrancken et al. based on a maximum-likelihood phylogeny of Alberta sequences alongside Los Alamos HIV database sequences (http://hiv.lanl.gov), confirmed using Comet (Struck et al. 2014). Four sequences were removed because they were duplicates and one sequence was removed because it had >0.05 ambiguous nucleotides, leaving 804 sequences. Sequences were aligned using mafft v7.402 (Katoh et al. 2005) and known drug resistance mutation sites relative to HXB2 reference were removed (Shafer 2006). Similarly to Vrancken et al., we identified a weak temporal signal within an approximate maximum-likelihood tree of all subtype B sequences inferred using FastTree v2.1.11 (Price et al. 2010) and rooted by residual mean squared (rms) regression fit in Tempest (Rambaut et al. 2016). The subtype B phylogeny consisted of two deeply split clades herein denoted B.1 (*n* = 624) and B.2 (*n* = 185). After splitting B.1 and B.2 at their MRCA into two trees, re-rooting using rms, and retaining only sequences with residuals <0.02 substitutions/site (B.1, *n* = 563; B.2, *n* = 164), this yielded an increase in the correlation coefficient of the molecular clock rate fit from 0.21 to 0.33 and 0.34, respectively. Here, we focused our analyses on the larger B.1 clade (*n* = 563).

The B.1 alignment was used to jointly infer a time-calibrated phylogeny and fit a BD skyline serial model in BEAST2 (Stadler et al. 2013; Bouckaert et al. 2019). Model selection consisted of comparing strict and relaxed uncorrelated log normal (UCLN) clocks with free and fixed mean clock rates, as well as multiple rate intervals for $R_{e}$, *S*, and δ using nested sampling (Russel et al. 2019). For all the models compared, site model averaging was conducted using bModelTest (Bouckaert and Drummond 2017); additional priors are summarized in S2. UCLN models with free mean clock rates were more well-supported than their fixed mean clock rate or strict clock model equivalents; and models with rate shifts occurring in 1998 and 2010 had higher likelihoods than their equivalents with equally spaced intervals from origin to the most recent sample. For every model, two parallel MCMC chains of 500 million steps were combined after confirming each run converged, as assessed by effective sample sizes greater than 200 following 10% burn-in. The probability densities of BDS model parameters, based on samples drawn by BEAST from the posterior distribution, are shown in supplementary figure S22, Supplementary Material online. The posterior distributions of molecular evolution parameters are shown in supplementary figure S23, Supplementary Material online. MCMC trace plots are shown in supplementary figure S24, Supplementary Material online.

## Supplementary Material

Supplementary data are available at *Molecular Biology and Evolution* online.

## Supplementary Material

msab149_Supplementary_Data
