# Supplementary material for: Fundamental Identifiability Limits in Molecular Epidemiology
Source: Mol Biol Evol. 2021 May 19;38(9):4010–24. doi: 10.1093/molbev/msab149 (PMC8382926; doi:10.1093/molbev/msab149)
Supplement: msab149_Supplementary_Data [file msab149_supplementary_data.zip › supplement (1).pdf]

Fundamental identifiability limits in molecular epidemiology
# - Supplemental Information -

Stilianos Louca[1,2 *], Angela McLaughlin[3,4], Ailene MacPherson[5,6,7], Jeffrey B. Joy[3,4,8] & Matthew W. Pennell[5,6 *]

[1] *Department of Biology, University of Oregon, USA*
[2] *Institute of Ecology and Evolution, University of Oregon, USA*
[3] *British Columbia Centre for Excellence in HIV/AIDS, Vancouver, Canada*
[4] *Bioinformatics, University of British Columbia, Vancouver, Canada*
[5] *Biodiversity Research Centre, University of British Columbia, Vancouver, Canada*
[6] *Department of Zoology, University of British Columbia, Vancouver, Canada*
[7] *Department of Ecology and Evolutionary Biology, University of Toronto, Toronto, Canada*
[8] *Department of Medicine, University of British Columbia, Vancouver, Canada*
[*] Correspondence should be addressed to SL and MWP

Contents

S.1 Mathematical derivations **3**

S.1.1 General model . . . 3

S.1.2 Pulled parameters . . . 4

S.1.3 Congruent models . . . 6

S.1.4 Congruent models have identical distributions of tree sizes . . . 8

S.1.5 Constructing congruent scenarios . . . 9

S.1.5.1 Congruent scenarios by specifying ψ . . . 9

S.1.5.2 Congruent scenarios by specifying μ and $\lambda(\tau_o)$ . . . 10

S.1.5.3 Congruent scenarios by specifying μ and $\psi(\tau_o)$ . . . 10

S.1.5.4 Congruent scenarios by specifying λ . . . 10

S.1.5.5 Congruent scenarios by specifying R_{e} and $\lambda(\tau_o)$ . . . 11

S.1.5.6 Congruent scenarios by specifying $\mu + \psi$ and $\lambda(\tau_o)$ . . . 11

S.1.5.7 Congruent scenarios by specifying $\mu + \psi$ and $S(\tau_o)$ . . . 12

S.1.5.8 Congruent scenarios by specifying $\psi/(\mu + \psi)$ and $\lambda(\tau_o)$ . . . 13

S.1.6 The information content of concentrated sampling attempts . . . 13

S.1.7 Infinitesimally short concentrated sampling attempts . . . 15

S.2 Why common model selection methods cannot resolve congruencies **16**

S.2.1 General considerations (big picture) . . . 16

S.2.2 Regularization . . . 17

S.2.3 AIC and BIC . . . 17

S.3 Statistical evaluation of multiple simulated scenarios **18**

S.4 Overview of computer code **19**

S.5 On the use of occurrence data with phylogenies **20**

S.1 Mathematical derivations

S.1.1 General model

The general cladogenic model considered here, and recently formally described by MacPherson *et al.* [1], is a stochastic birth-death-sampling model, where lineages split ("speciate") stochastically at some birth rate λ, disappear ("go extinct") at some death rate μ, and are sampled (detected) at rate ψ. In an epidemiological context, λ corresponds to the transmission rate, μ to the rate of recovery or death of infected individuals, and ψ to the rate at which infected individuals are detected and their pathogen population sequenced. Note that, unless stated otherwise, throughout this document the term "birth" refers to the emergence of a new pathogen lineage (via transmission) and "death" refers to the disappearance of a pathogen lineage without sampling (due to host recovery or death), and should not be confused with host birth or death. The parameters λ, μ, and ψ can each depend arbitrarily on time. We consider the distribution of time-calibrated reconstructed phylogenies ("timetrees") obtained through this process, i.e., connecting all sampled variants, after some time $\tau_{\rm or}$. Note that this cladogenic process is a generalization of the birth-death-skyline model by Stadler *et al.* [2] to the case where λ, μ, and ψ depend arbitrarily on time rather than in a piecewise constant manner, in the absence of concentrated sampling attempts.

In the following, we count time backward from tips to root, i.e., all variables are expressed as functions of "age" (time before present), with age zero corresponding to the time at which the sampling process is halted. It was recently shown [1] that the likelihood (density) of a given bifurcating timetree generated by a specific BDS scenario starting at age $\tau_{\rm or}$, conditioned upon the survival and sampling of at least one lineage, is given by:

$$L_{\rm or} = \frac{\Phi(\tau_{\rm or})}{1 - E(\tau_{\rm or})} \prod_{i=1}^{n-1} \lambda(x_i)\Phi(x_i) \prod_{j=1}^{n} \frac{\psi(y_j)}{\Phi(y_j)}, \tag{1}$$

where n is the number of tips in the tree, i iterates over all branching events (i.e., internal nodes), j iterates over all sampling events (i.e., tips), x_i is the age of the i-th branching event, y_j is the age of the j-th sampling event, $E(\tau)$ is the probability that a lineage alive at age τ will not be sampled by the present-day (i.e., is not included in the tree), and Φ is the "flow" of the model [3], defined as:

$$\Phi(\tau) = \exp\left[\int_0^\tau \left[2\lambda E - (\lambda + \mu + \psi)\right] \, ds\right]. \tag{2}$$

The probability E satisfies the differential equation:

$$\frac{dE}{d\tau} = -(\lambda + \mu + \psi)E + \lambda E^2 + \mu, \tag{3}$$

with initial condition $E(0) = 1$. Note that one may alternatively consider the likelihood of the tree conditioned on the age of the tree's root $\tau_{\rm r}$, i.e., a splitting occurring at age $\tau_{\rm r}$ and the eventual sampling of both child lineages:

$$L_{\rm r} = \frac{\Phi(\tau_{\rm r})}{\lambda(\tau_{\rm r}) \cdot (1 - E(\tau_{\rm r}))^2} \prod_{i=1}^{n-1} \lambda(x_i)\Phi(x_i) \prod_{j=1}^{n} \frac{\psi(y_j)}{\Phi(y_j)}. \tag{4}$$

In the limit of infinitely large trees, i.e., when stochastic effects on the LTT curve become negligible, the deterministic LTT curve (dLTT) predicted by the model, denoted M, is equal to $M = N \cdot (1 - E)$, where

$N(\tau)$ is the total number of lineages alive at age τ. Using the fact that $dN/dt = (\lambda - \mu - \psi) \cdot N$ as well as Eq. (3), it is straightforward to derive the following differential equation for the model's dLTT:

$$\frac{dM}{dt} = -M \cdot \left[(1 - E) \cdot \lambda - \frac{\psi}{1 - E} \right]. \tag{5}$$

Note that to calculate the dLTT one also needs to specify an initial condition, i.e., conditioned on the number of lineages at some specific age $\tau > 0$ ($M(0)$ will always be zero).

S.1.2 Pulled parameters

The following composite parameters will be particular useful in our derivations. We define the "pulled birth rate" $\tilde{\lambda}$ as follows:

$$\tilde{\lambda} = (1 - E) \cdot \lambda. \tag{6}$$

Note that $\tilde{\lambda}$ is equal to λ if and only if $E = 0$, i.e., if all lineages ever alive are represented in the tree. In the presence of deaths or incomplete sampling, however, $\tilde{\lambda}$ is "pulled" downwards compared to λ. Observe that $\tilde{\lambda}$ is the expected rate at which internal nodes occur in the tree over time, normalized by the current number of lineages in the tree (M) and in the limit of infinitely large trees (see Eq. 5). For sufficiently large trees $\tilde{\lambda}$ becomes identifiable, since it can just be "read off" the tree's LTT. We also define the "pulled sampling rate" $\tilde{\psi}$ as follows:

$$\tilde{\psi} = \frac{\psi}{1 - E}. \tag{7}$$

Note that $\tilde{\psi}$ is equal to ψ if and only if $E = 0$; otherwise, $\tilde{\psi}$ is pulled upward compared to ψ. Observe that $\tilde{\psi}$ is the expected rate at which tips occur in the tree over time, normalized by the current number of lineages in the tree (M) and in the limit of infinitely large trees (see Eq. 5). For sufficiently large trees $\tilde{\psi}$ becomes identifiable, since it can just be "read off" the tree's LTT. We also define the following composite parameter:

$$\tilde{\zeta} = \lambda\psi. \tag{8}$$

Note that for $\tau > 0$ we have $\tilde{\zeta}(\tau) = \tilde{\lambda}(\tau)\tilde{\psi}(\tau)$. In contrast to $\tilde{\psi}$, which becomes infinite at present day (i.e., at age 0), the parameter $\tilde{\zeta}$ remains finite. Since $\tilde{\lambda}$ and $\tilde{\psi}$ are asymptotically identifiable (i.e., in the limit of infinitely large trees), the same also holds for $\tilde{\zeta}$. We also define the "pulled diversification rate" $\tilde{r}$ as follows:

$$\tilde{r} = \lambda - \mu - \psi + \frac{1}{\lambda} \frac{d\lambda}{d\tau}. \tag{9}$$

Note that $\tilde{r}$ is equal to the net diversification rate $r = \lambda - \mu - \psi$ only if λ is constant over time. If λ varies over time, $\tilde{r}$ is pulled upwards or downwards compared to r, depending on how λ varies with time. Taking the derivative on both sides of Eq. (6) and using Eqs. (3) and (8) it is straightforward to show that $\tilde{\lambda}$ satisfies the differential equation:

$$\frac{d\tilde{\lambda}}{d\tau} = \tilde{\lambda} \cdot \left(\tilde{r} - \tilde{\lambda} + \tilde{\psi} \right). \tag{10}$$

Note that we can also write Eq. (10) as:

$$\frac{d\tilde{\lambda}}{d\tau} = \tilde{\lambda}\tilde{r} - \tilde{\lambda}^2 + \tilde{\zeta}. \tag{11}$$

Hence, $\tilde{\lambda}$ is fully determined by $\tilde{r}$ and $\tilde{\zeta}$ (note that $\tilde{\lambda}$ always satisfies the initial condition $\tilde{\lambda}(0) = 0$). Solving Eq. (10) for $\tilde{r}$ yields:

$$\tilde{r} = \frac{1}{\tilde{\lambda}}\frac{d\tilde{\lambda}}{d\tau} + \tilde{\lambda} - \tilde{\psi}. \tag{12}$$

Since $\tilde{\lambda}$ and $\tilde{\psi}$ are asymptotically identifiable, the same also holds for $\tilde{r}$. Solving Eq. (10) for $\tilde{\psi}$ yields:

$$\tilde{\psi} = \frac{1}{\tilde{\lambda}}\frac{d\tilde{\lambda}}{d\tau} + \tilde{\lambda} - \tilde{r}. \tag{13}$$

Thus, $\tilde{\psi}$ is fully determined by $\tilde{\lambda}$ and $\tilde{r}$. Multiplying Eq. (13) by $\tilde{\lambda}$ yields:

$$\tilde{\zeta} = \tilde{\psi}\tilde{\lambda} = \frac{d\tilde{\lambda}}{d\tau} + \tilde{\lambda}^2 - \tilde{r}\tilde{\lambda}. \tag{14}$$

Hence, $\tilde{\zeta}$ is fully determined by $\tilde{\lambda}$ and $\tilde{r}$. It is also worth introducing three additional parameters, the "normalized dLTT":

$$\tilde{M} := \frac{M}{\int_0^\infty M(s)ds}, \tag{15}$$

the "deterministic branching density":

$$\tilde{\beta} := \tilde{\lambda}\tilde{M} = \frac{\tilde{\lambda}M}{\int_0^\infty M(s)\,ds}, \tag{16}$$

and the "deterministic sampling density":

$$\tilde{\sigma} := \tilde{\psi}\tilde{M} = \frac{\tilde{\psi}M}{\int_0^\infty M(s)\,ds}. \tag{17}$$

Note that neither $\tilde{M}$, $\tilde{\beta}$ nor $\tilde{\sigma}$ depend on the specific dLTT, as long as the latter solves the differential equation (5), in other words $\tilde{M}$, $\tilde{\beta}$ and $\tilde{\sigma}$ are independent of the specific initial condition chosen for M. Hence, $\tilde{M}$, $\tilde{\beta}$ and $\tilde{\sigma}$ are fully determined by $\tilde{\lambda}$ and $\tilde{\psi}$, and are thus asymptotically identifiable. Reciprocally, $\tilde{M}$, $\tilde{\lambda}$ and $\tilde{\psi}$ are fully determined by $\tilde{\beta}$ and $\tilde{\sigma}$. Indeed, from Eq. (5) we have:

$$\frac{d\tilde{M}}{d\tau} = -\tilde{M}\cdot\left(\tilde{\lambda} - \tilde{\psi}\right) = \tilde{\sigma} - \tilde{\beta}, \tag{18}$$

with initial condition $X(0) = 0$. Hence:

$$\tilde{M} = \int_0^\tau (\tilde{\sigma}(s) - \tilde{\beta}(s))\,ds, \tag{19}$$

and:

$$\tilde{\lambda} = \frac{\tilde{\beta}}{\tilde{M}}, \tag{20}$$

and:

$$\tilde{\psi} = \frac{\tilde{\sigma}}{\tilde{M}}. \tag{21}$$

The normalized dLTT is simply the dLTT rescaled so that its area-under-the-curve is equal to 1; hence, the normalized dLTT is a property of the particular BDS scenario that does not depend on the specific initial condition for M, and which can be interpreted as a probability density over time. The numerator $\tilde{\lambda}(\tau)M(\tau)$ on the right-hand-side of Eq. (16) is the expected rate of branching events observed in the tree at age τ, while the denominator $\int_0^\infty M(s)ds$ merely acts as a rescaling. Hence the normalized version of $\tilde{\beta}$ (i.e., if divided by its integral $\int_0^\infty \tilde{\beta}(s)ds$) can be interpreted as the probability density on $[0,\infty)$ for any randomly chosen observed branching event. The normalization of $\tilde{\beta}$, however, is such that the integral $\int_0^\infty \tilde{\beta}(s)ds$ is equal to the average pulled birth rate, where the average is calculated based on the normalized dLTT:

$$\int_0^\infty \tilde{\beta}(\tau)d\tau = \int_0^\infty \tilde{\lambda}(s)\tilde{M}(s)\, ds. \tag{22}$$

In particular, $\tilde{\beta}$ contains information not just about the shape of the branching rate over time, but also about the absolute magnitude of the pulled birth rate. A similar interpretation exists for the deterministic sampling density $\tilde{\sigma}$, i.e. $\tilde{\psi}$ is the (non-normalized) probability density on $[0,\infty)$ for any randomly chosen observed tip. In contrast to $\tilde{\psi}$, the sampling density $\tilde{\sigma}$ stays finite at age 0:

$$\tilde{\sigma} = \frac{\psi N}{\displaystyle\int_0^\infty M(s)\, ds}. \tag{23}$$

Note that the interpretations of some of the above parameters are strictly speaking only valid in the limit of infinitely large trees, however the parameters themselves are simply properties of the BDS scenario and thus well-defined regardless of the size of the tree.

S.1.3 Congruent models

From Eq. (1) and Eq. (4) it becomes clear that the likelihood of a timetree only depends on the sampling and branching ages in the tree, but not the precise tree topology itself. As we show below, a similar statement can also be made about the model's role, as follows: Any two birth-death-sampling scenarios with the same pulled birth rate $\tilde{\lambda}$ and the same pulled sampling rate $\tilde{\psi}$ will always yield the same likelihoods. We thus henceforth call two birth-death-sampling scenarios "congruent" if they have the same $\tilde{\lambda}$ and the same $\tilde{\psi}$. Equivalently, following Supplement S.1.2, two scenarios are congruent if and only if they have the same $\tilde{\lambda}$ and the same $\tilde{r}$, or equivalently, if and only if they have the same $\tilde{\lambda}$ and the same $\tilde{\zeta}$, or equivalently, if and only if they have the same $\tilde{r}$ and the same $\tilde{\zeta}$, or equivalently, if and only if they have the same deterministic branching density $\tilde{\beta}$ and the same deterministic sampling density $\tilde{\sigma}$.

To prove that congruent BDS scenarios have the same likelihoods regardless of the data, we proceed as

follows. From Eq. (2) we have:

$$
\begin{aligned}
\Phi(\tau) =& \exp\left[\int_0^\tau [2\lambda E - (\lambda+\mu+\psi)]\ ds\right] \\
=& \exp\left[\int_0^\tau \left[2\lambda - 2\tilde{\lambda} - \lambda - \mu - \psi\right]\ ds\right] \\
=& e^{-2\tilde{\Lambda}(\tau)} \exp\left[\int_0^\tau [\lambda - \mu - \psi]\ ds\right],
\end{aligned}
\tag{24}
$$

where we defined:

$$
\tilde{\Lambda}(\tau) := \int_0^\tau \tilde{\lambda}(s)\ ds. \tag{25}
$$

Using Eq. (9) we can further write:

$$
\begin{aligned}
\Phi(\tau) =& e^{-2\tilde{\Lambda}(\tau)} \exp\left[\int_0^\tau \left[\tilde{r} - \frac{1}{\lambda}\frac{d\lambda}{ds}\right]\ ds\right] \\
=& e^{-2\tilde{\Lambda}(t)+\tilde{R}(t)} \cdot \exp\left[-\int_0^\tau \frac{d\ln\lambda}{ds}\ ds\right] \\
=& e^{-2\tilde{\Lambda}(t)+\tilde{R}(t)} \cdot \frac{\lambda(0)}{\lambda(\tau)},
\end{aligned}
\tag{26}
$$

where we defined:

$$
\tilde{R}(\tau) := \int_0^\tau \tilde{r}(s)\ ds. \tag{27}
$$

Using Eq. (26) in the formula for the likelihood, Eq. (1), yields:

$$
\begin{aligned}
L_{\text{or}} =& \frac{\Phi(\tau_{\text{or}})}{1-E(\tau_{\text{or}})} \prod_{i=1}^{n-1} \lambda(x_i)\Phi(x_i) \prod_{j=1}^{n} \frac{\psi(y_j)}{\Phi(y_j)} \\
=& \frac{e^{-2\tilde{\Lambda}(\tau_{\text{or}})+\tilde{R}(\tau_{\text{or}})}\lambda(0)}{\lambda(\tau_{\text{or}})(1-E(\tau_{\text{or}}))} \prod_{i=1}^{n-1} \lambda(x_i) e^{-2\tilde{\Lambda}(x_i)+\tilde{R}(x_i)} \frac{\lambda(0)}{\lambda(x_i)} \prod_{j=1}^{n} \psi(y_j) e^{2\tilde{\Lambda}(y_j)-\tilde{R}(y_j)} \frac{\lambda(y_j)}{\lambda(0)} \\
=& \frac{e^{-2\tilde{\Lambda}(\tau_{\text{or}})+\tilde{R}(\tau_{\text{or}})}}{\tilde{\lambda}(\tau_{\text{or}})} \prod_{i=1}^{n-1} e^{-2\tilde{\Lambda}(x_i)+\tilde{R}(x_i)} \prod_{j=1}^{n} e^{2\tilde{\Lambda}(y_j)-\tilde{R}(y_j)} \psi(y_j)\lambda(y_j).
\end{aligned}
\tag{28}
$$

Since $\lambda\psi = \tilde{\zeta}$, we can further write:

$$
\boxed{L_{\text{or}} = \frac{e^{-2\tilde{\Lambda}(\tau_{\text{or}})+\tilde{R}(\tau_{\text{or}})}}{\tilde{\lambda}(\tau_{\text{or}})} \prod_{i=1}^{n-1} e^{-2\tilde{\Lambda}(x_i)+\tilde{R}(x_i)} \prod_{j=1}^{n} e^{2\tilde{\Lambda}(y_j)-\tilde{R}(y_j)} \tilde{\zeta}(y_j).} \tag{29}
$$

Note that the likelihood in Eq. (29) depends solely on the variables $\tilde{\lambda}$, $\tilde{r}$, $\tilde{\zeta}$, $\tilde{\Lambda}$ and $\tilde{R}$, all of which are fully determined by the pulled birth rate $\tilde{\lambda}$ and pulled sampling rate $\tilde{\psi}$.

Using a similar approach one can also write the likelihood L_{r}, i.e., conditioned on the root age, in a format

that only depends on pulled variables:

$$\boxed{L_{\mathrm{r}} = \frac{e^{-2\tilde{\Lambda}(\tau_{\mathrm{r}})+\tilde{R}(\tau_{\mathrm{r}})}}{\tilde{\lambda}^2(\tau_{\mathrm{r}})} \prod_{i=1}^{n-1} e^{-2\tilde{\Lambda}(x_i)+\tilde{R}(x_i)} \prod_{j=1}^{n} e^{2\tilde{\Lambda}(y_j)-\tilde{R}(y_j)}\tilde{\zeta}(y_j).} \tag{30}$$

□

S.1.4 Congruent models have identical distributions of tree sizes

In the following we show that any two congruent BDS scenarios will generate trees whose size (i.e., number of tips) has the same probability distributions, when conditioned on the age of the stem and the sampling of at least one tip (or the age of the root, and the sampling of both daughter lineages). Let $P_n(\tau)$ be the probability that a lineage alive at age τ will have exactly n sampled descendants in the final tree (i.e., at present-day, where the process ends). It is straightforward to show that P_n (for any $n \in \{0, 1, 2, ..\}$) satisfies the differential equation:

$$\frac{dP_n}{d\tau} = \lambda \sum_{k=0}^{n} P_k P_{n-k} + \mu\delta_{n,0} + \psi\delta_{n,1} - (\lambda + \mu + \psi)P_n, \tag{31}$$

with initial condition:

$$P_n(0) = \delta_{n,0}, \tag{32}$$

where τ denotes age and δ denotes the Kronecker-delta symbol, i.e. $\delta_{n,k}$ is 1 if $k = n$ and 0 otherwise. Denote $\tilde{P}_n := P_n/(1 - E)$. We wish to show that $\tilde{P}_n$ is the same for any two congruent BDS scenarios, for any $n > 0$. We have:

$$\begin{aligned}
\frac{d\tilde{P}_n}{d\tau} =& \frac{1}{1-E}\frac{dP_n}{d\tau} + \frac{P_n}{(1-E)^2}\frac{dE}{d\tau} \\
\overset{(31)}{=}& \frac{1}{(1-E)^2}\Bigg[(1-E)\left(\lambda \sum_{k=0}^{n} P_k P_{n-k} + \psi\delta_{n,1} - (\lambda+\mu+\psi)P_n\right) \\
&+ \Big(\lambda E^2 + \mu - (\lambda+\mu+\psi)E\Big) P_n\Bigg] \\
=& \frac{1}{(1-E)^2}\Bigg[\underbrace{\lambda(1-E)}_{\tilde{\lambda}} \sum_{k=1}^{n-1} P_k P_{n-k} + 2\lambda E P_n + (1-E)\psi\delta_{n,1} - (\lambda+\psi)P_n - \lambda E^2 P_n\Bigg] \qquad (33) \\
=& \tilde{\lambda} \sum_{k=1}^{n-1} \tilde{P}_k \tilde{P}_{n-k} + \underbrace{\frac{\psi}{1-E}}_{\tilde{\psi}}\delta_{n,1} - \underbrace{\frac{\psi}{1-E}}_{\tilde{\psi}} \cdot \underbrace{\frac{P_n}{1-E}}_{\tilde{P}_n} - \underbrace{\frac{P_n}{1-E}}_{\tilde{P}_n} \cdot \underbrace{(1-E)\lambda}_{\tilde{\lambda}} \\
=& \tilde{\lambda} \sum_{k=1}^{n-1} \tilde{P}_k \tilde{P}_{n-k} + \tilde{\psi}\delta_{n,1} - \tilde{\psi}\tilde{P}_n - \tilde{\lambda}\tilde{P}_n.
\end{aligned}$$

Further, according to L'Hopital's rule one has:

$$\lim_{\tau\to 0^+}\tilde{P}_n(\tau)=\lim_{\tau\to 0^+}\frac{P_n'(\tau)}{1-E'(\tau)}=\lim_{\tau\to 0^+}\frac{\psi(0)\delta_{n,1}}{\psi(0)}=\delta_{n,1}. \tag{34}$$

Since any two congruent scenarios have the same $\tilde{\lambda}$ and the same $\tilde{\psi}$, each of their $\tilde{P}_n$ (with $n \geq 1$) will satisfy the same differential equation (33) with the same initial condition (34). We thus conclude that any two congruent scenarios will have the same conditional probabilities $\tilde{P}_n$.

A similar conclusion can be drawn for the case where we condition on the age of the root and the survival of its two child lineages, as follows. Denote by $\hat{P}_n(\tau)$ the conditional probability that the tree has size n, given that the root split at age τ and that both of its child lineages survived. Then for any $n \geq 2$ we have:

$$\hat{P}_n=\frac{1}{(1-E)^2}\sum_{k=1}^{n-1}P_kP_{n-k}=\sum_{k=1}^{n-1}\tilde{P}_k\tilde{P}_{n-k}. \tag{35}$$

Since the $\tilde{P}_n$ are identical between congruent scenarios (for any $n \geq 1$), we conclude that $\hat{P}_n$ are also identical between congruent scenarios (for any $n \geq 2$).

□

S.1.5 Constructing congruent scenarios

For any given BDS scenario (λ,μ,ψ) there are various approaches towards constructing alternative congruent scenarios, described below. All of the presented methods have in common that one first calculates the "pulled variables" of the original scenario (which fully define the scenario's congruence class), and then provides sufficient additional constraints to obtain a new single scenario within the congruence class. The discussion below also reveals how scenarios can become fully identifiable when additional constraints are provided.

S.1.5.1 Congruent scenarios by specifying ψ

In the first approach, we specify a new sampling rate ψ^* and then adjust the remaining model variables to obtain a congruent scenario. Specifically, let $\tilde{\lambda}$, $\tilde{r}$, and $\tilde{\zeta}$ be the pulled variables of the scenario as defined in Supplement S.1.2. Let $\psi^* > 0$ be some arbitrary alternative sampling rate. Define $\lambda^* := \tilde{\zeta}/\psi^*$ and:

$$\mu^* := \lambda^* - \psi^* + \frac{1}{\lambda^*}\frac{d\lambda^*}{d\tau} - \tilde{r} \tag{36}$$

Then the pulled diversification rate of the new scenario (λ^*,μ^*,ψ^*) is:

$$\tilde{r}^* = \lambda^* - \mu^* - \psi^* + \frac{1}{\lambda^*}\frac{d\lambda^*}{d\tau} \overset{(36)}{=} \lambda^* - \lambda^* + \psi^* - \frac{1}{\lambda^*}\frac{d\lambda^*}{d\tau} + \tilde{r} - \psi^* + \frac{1}{\lambda^*}\frac{d\lambda^*}{d\tau} = \tilde{r}. \tag{37}$$

Further, $\tilde{\zeta}^* = \lambda^*\psi^* = \tilde{\zeta}$. Hence, the new scenario has the same $\tilde{r}$ and the same $\tilde{\zeta}$ as the original scenario. By Supplement S.1.3, the two scenarios are thus congruent. Hence, simply by choosing an alternative sampling rate ψ^*, one can find a corresponding congruent scenario (λ^*,μ^*,ψ^*), as long as the resulting μ^* is physically meaningful (i.e., non-negative). Reciprocally, if the congruence class of a diversification/epidemiological scenario is known (e.g., estimated via maximum-likelihood), and in addition ψ is somehow independently

estimated, then the full scenario can be reconstructed.

S.1.5.2 Congruent scenarios by specifying μ and $\lambda(\tau_o)$

Instead of first specifying an alternative ψ^*, one could construct congruent scenarios by first specifying μ^* and the birth rate $\lambda^*(\tau_o)$ at some age τ_o, as follows. Let $\tilde{r}$ and $\tilde{\zeta}$ be the pulled variables of the scenario (λ, μ, ψ) as defined in Supplement S.1.2. For any given death rate profile μ^*, age τ_o and birth rate $\lambda_o^* \geq 0$ at age τ_o, choose λ^* as the solution of the following differential equation:

$$\frac{d\lambda^*}{d\tau} = \lambda^* \cdot [\tilde{r} + \mu^* - \lambda^*] + \tilde{\zeta}, \tag{38}$$

with condition $\lambda^*(\tau_o) = \lambda_o^*$, and set $\psi^* := \tilde{\zeta}/\lambda^*$. The pulled diversification rate of the new scenario $(\lambda^*, \mu^*, \psi^*)$ is given by:

$$\tilde{r}^* = \lambda^* - \mu^* - \psi^* + \frac{1}{\lambda^*}\frac{d\lambda^*}{d\tau} \overset{(38)}{=} \lambda^* - \mu^* - \psi^* - [\lambda^* - \tilde{r} - \mu^*] + \frac{\tilde{\zeta}}{\lambda^*} = \tilde{r}. \tag{39}$$

Further, $\tilde{\zeta}^* = \lambda^*\psi^* = \tilde{\zeta}$. Hence, the new scenario has the same $\tilde{r}$ and the same $\tilde{\zeta}$ as the original scenario. By Supplement S.1.3, the two scenarios are thus congruent. Hence, simply by choosing an alternative sampling rate μ^* and an arbitrary initial condition $\lambda^*(\tau_o)$ at some age τ_o, one can find a corresponding congruent scenario $(\lambda^*, \mu^*, \psi^*)$, provided that the resulting λ^* is physically meaningful (i.e., non-negative). Reciprocally, if the congruence class of a diversification/epidemiological scenario is known (e.g., estimated via maximum-likelihood), and in addition μ and $\lambda(\tau_o)$ are somehow independently obtained (e.g., from clinical experiments), then the full scenario can be reconstructed.

Note that, as long as $\lambda\psi > 0$ and $\lambda^*(0) > 0$, and as long as $\tilde{r}$, $\tilde{\zeta}$ and μ^* are continuous, the corresponding λ^* will always be strictly positive. To see why this is the case, observe that if λ^* was zero or negative at some point, there would exist an age τ_z where $\lambda^*(\tau_z) = 0$ and $\lambda^*(\tau) > 0$ for all $\tau < \tau_z$. Since $\tilde{\zeta}(\tau_z) = \lambda(\tau_z)\psi(\tau_z) > 0$ and $\lambda^*(\tau_z) \cdot [\tilde{r}(\tau_z) + \mu^*(\tau_z) - \lambda^*(\tau_z)] = 0$, by Eq. (38) the derivative $d\lambda^*/d\tau$ must be strictly positive at τ_z, and in fact strictly positive in a small neighborhood of τ_z, which would mean λ^* could not reach zero at τ_z — a contradiction.

S.1.5.3 Congruent scenarios by specifying μ and $\psi(\tau_o)$

Alternatively to specifying the profile μ^* and the value $\lambda^*(\tau_o)$ at some age τ_o, one could also specify the profile μ^* and the value $\psi^*(\tau_o)$. In that case, simply choose $\lambda^*(\tau_o) = \tilde{\zeta}(\tau_o)/\psi^*(\tau_o)$, and apply the procedure in Supplement S.1.5.2 to obtain a congruent scenario $(\lambda^*, \mu^*, \psi^*)$.

S.1.5.4 Congruent scenarios by specifying λ

Another way of constructing congruent scenarios is to first specify λ^* and then choose μ^* and ψ^* accordingly, as follows. Let $\tilde{r}$ and $\tilde{\zeta}$ be the pulled variables of the scenario (λ, μ, ψ) as defined in Supplement S.1.2. For any given λ^*, choose $\psi^* := \tilde{\zeta}/\lambda^*$ and:

$$\mu^* := \lambda^* - \psi^* + \frac{1}{\lambda^*}\frac{d\lambda^*}{d\tau} - \tilde{r}. \tag{40}$$

Then the pulled diversification rate of the new scenario $(\lambda^*, \mu^*, \psi^*)$ is:

$$\tilde{r}^* = \lambda^* - \mu^* - \psi^* + \frac{1}{\lambda^*}\frac{d\lambda^*}{d\tau} \overset{(40)}{=} \lambda^* - \lambda^* + \psi^* - \frac{1}{\lambda^*}\frac{d\lambda^*}{d\tau} + \tilde{r} - \psi^* + \frac{1}{\lambda^*}\frac{d\lambda^*}{d\tau} = \tilde{r}. \tag{41}$$

Hence, the new scenario has the same $\tilde{r}$ and the same $\tilde{\zeta}$ as the original scenario. The two scenarios are thus congruent. Hence, simply by choosing an alternative sampling rate λ^*, one can find a corresponding congruent scenario $(\lambda^*, \mu^*, \psi^*)$, as long as the resulting μ^* is physically meaningful (i.e., non-negative).

S.1.5.5 Congruent scenarios by specifying $R_{\rm e}$ and $\lambda(\tau_o)$

Congruent scenarios can also be constructed by first choosing the effective reproduction ratio profile and the birth rate $\lambda^*(\tau_o)$ at some age τ_o, as follows. Let $\tilde{r}$ and $\tilde{\zeta}$ be the pulled variables of the scenario (λ, μ, ψ) as defined in Supplement S.1.2. For any given basic reproduction ratio profile $R_{\rm e}^*$, age τ_o and birth rate $\lambda_o^* \geq 0$ at age τ_o, choose λ^* as the solution of the following differential equation:

$$\frac{d\lambda^*}{d\tau} = \lambda^* \cdot \left[\lambda^* \cdot \left(\frac{1}{R_{\rm e}} - 1\right) + \tilde{r}\right], \tag{42}$$

with condition $\lambda^*(\tau_o) = \lambda_o^*$, and set $\psi^* := \tilde{\zeta}/\lambda^*$ and $\mu^* := \lambda^*/R_{\rm e} - \psi^*$. The pulled diversification rate of the new scenario $(\lambda^*, \mu^*, \psi^*)$ is given by:

$$\tilde{r}^* = \lambda^* - \mu^* - \psi^* + \frac{1}{\lambda^*}\frac{d\lambda^*}{d\tau} = \lambda^* - \frac{\lambda^*}{R_{\rm e}} + \psi^* - \psi^* + \lambda^* \cdot \left(\frac{1}{R_{\rm e}} - 1\right) + \tilde{r} = \tilde{r}. \tag{43}$$

Further, $\tilde{\zeta}^* = \lambda^*\psi^* = \tilde{\zeta}$. Hence, the new scenario has the same $\tilde{r}$ and the same $\tilde{\zeta}$ as the original scenario. By Supplement S.1.3, the two scenarios are thus congruent. Hence, simply by choosing an alternative $R_{\rm e}^*$ and an arbitrary condition $\lambda^*(\tau_o) = \lambda_o^*$ at some age τ_o, one can find a corresponding congruent scenario $(\lambda^*, \mu^*, \psi^*)$, provided that the resulting λ^* and μ^* are physically meaningful (i.e., non-negative). Reciprocally, if the congruence class of a diversification/epidemiological scenario is known (e.g., estimated via maximum-likelihood), and in addition $R_{\rm e}$ and $\lambda(\tau_o)$ are somehow independently obtained, then the full scenario can be reconstructed.

Note that the differential equation (42) is of Bernoulli type, and can thus be solved analytically:

$$\lambda^*(\tau) = \frac{\lambda_o^* e^{\tilde{R}(\tau)}}{e^{\tilde{R}(\tau_o)} + \left(1 - \frac{1}{R_{\rm e}^*}\right)\lambda_o^* \int_{\tau_o}^{\tau} e^{\tilde{R}(s)}\, ds}, \tag{44}$$

where $\tilde{R}$ is defined as in Eq. (27).

S.1.5.6 Congruent scenarios by specifying $\mu + \psi$ and $\lambda(\tau_o)$

Congruent scenarios can also be constructed by first choosing the total removal rate $\delta := \mu + \psi$ and the birth rate $\lambda^*(\tau_o)$ at some age τ_o, as follows. Let $\tilde{r}$ and $\tilde{\zeta}$ be the pulled variables of the scenario (λ, μ, ψ) as defined in Supplement S.1.2. For any given profile $\delta^* \geq 0$, age τ_o and birth rate $\lambda_o^* \geq 0$ at age τ_o, choose λ^* as the

solution of the following differential equation:

$$\frac{d\lambda^*}{d\tau} = \lambda^* \cdot (\delta^* + \tilde{r} - \lambda^*), \tag{45}$$

with condition $\lambda^*(\tau_o) = \lambda_o^*$, and set $\psi^* := \tilde{\zeta}/\lambda^*$ and $\mu^* := \delta^* - \psi^*$. The pulled diversification rate of the new scenario $(\lambda^*, \mu^*, \psi^*)$ is given by:

$$\tilde{r}^* = \lambda^* - \mu^* - \psi^* + \frac{1}{\lambda^*}\frac{d\lambda^*}{d\tau} = \lambda^* - \delta^* + \psi^* - \psi^* + (\delta^* + \tilde{r} - \lambda^*) = \tilde{r}. \tag{46}$$

Further, $\tilde{\zeta}^* = \lambda^*\psi^* = \tilde{\zeta}$. Hence, the new scenario has the same $\tilde{r}$ and the same $\tilde{\zeta}$ as the original scenario. By Supplement S.1.3, the two scenarios are thus congruent. Hence, simply by choosing an alternative δ^* and an arbitrary condition $\lambda^*(\tau_o) = \lambda_o^*$ at some age τ_o, one can find a corresponding congruent scenario $(\lambda^*, \mu^*, \psi^*)$, provided that the resulting λ^* and μ^* are physically meaningful (i.e., non-negative). Reciprocally, if the congruence class of a diversification/epidemiological scenario is known (e.g., estimated via maximum-likelihood), and in addition δ and $\lambda(\tau_o)$ are somehow independently obtained, then the full scenario can be reconstructed.

Note that the differential equation (45) is of Bernoulli type, and can thus be solved analytically:

$$\lambda^*(\tau) = \frac{\lambda_o^* e^{\tilde{R}(\tau)+D^*(\tau)}}{e^{\tilde{R}(\tau_o)+D^*(\tau_o)} + \lambda_o^* \int_{\tau_o}^{\tau} e^{\tilde{R}(s)+D^*(s)}\, ds}, \tag{47}$$

where $\tilde{R}$ is defined as in Eq. (27) and D^* is defined as:

$$D^*(\tau) := \int_0^{\tau} \delta^*(s)\, ds. \tag{48}$$

From Eq. (47) it becomes clear that if $\tau_o = 0$ and $\lambda_o^* \geq 0$, then $\lambda^*(\tau)$ will be non-negative for all $\tau \geq 0$ (i.e., physically meaningful).

S.1.5.7 Congruent scenarios by specifying $\mu + \psi$ and $S(\tau_o)$

Congruent scenarios can also be constructed by first choosing the total removal rate $\delta := \mu + \psi$ and the sampling proportion $S^*(\tau_o)$ at some age τ_o, as follows. Let $\tilde{r}$ and $\tilde{\zeta}$ be the pulled variables of the scenario (λ, μ, ψ) as defined in Supplement S.1.2. For any given profile $\delta^* > 0$, age τ_o and sampling proportion $S_o^* \in (0, 1]$ at age τ_o, choose λ^* as the solution of the following differential equation:

$$\frac{d\lambda^*}{d\tau} = \lambda^* \cdot (\delta^* + \tilde{r} - \lambda^*), \tag{49}$$

with initial condition $\lambda^*(\tau_o) = \tilde{\zeta}(\tau_o)/(S^*(\tau_o) \cdot \delta^*(\tau_o))$, and set $\psi^* := \tilde{\zeta}/\lambda^*$ and $\mu^* := \delta^* - \psi^*$. The pulled diversification rate of the new scenario $(\lambda^*, \mu^*, \psi^*)$ is given by:

$$\tilde{r}^* = \lambda^* - \mu^* - \psi^* + \frac{1}{\lambda^*}\frac{d\lambda^*}{d\tau} = \lambda^* - \delta^* + \psi^* - \psi^* + (\delta^* + \tilde{r} - \lambda^*) = \tilde{r}. \tag{50}$$

Further, $\tilde{\zeta}^* = \lambda^*\psi^* = \tilde{\zeta}$. Hence, the new scenario has the same $\tilde{r}$ and the same $\tilde{\zeta}$ as the original scenario. By Supplement S.1.3, the two scenarios are thus congruent. Hence, simply by choosing an alternative δ^*

and an arbitrary condition $S^*(\tau_o) = S_o^*$ at some age τ_o, one can find a corresponding congruent scenario $(\lambda^*, \mu^*, \psi^*)$, provided that the resulting λ^* and μ^* are physically meaningful (i.e., non-negative). Reciprocally, if the congruence class of a diversification/epidemiological scenario is known (e.g., estimated via maximum-likelihood), and in addition δ and $S(\tau_o)$ are somehow independently obtained, then the full scenario can be reconstructed.

S.1.5.8 Congruent scenarios by specifying $\psi/(\mu+\psi)$ and $\lambda(\tau_o)$

Congruent scenarios can also be constructed by first choosing the "sampling proportion" $S := \psi/(\mu+\psi)$ and the birth rate $\lambda^*(\tau_o)$ at some age τ_o, as follows. Let $\tilde{r}$ and $\tilde{\zeta}$ be the pulled variables of the scenario (λ, μ, ψ) as defined in Supplement S.1.2. For any given profile $S^* > 0$, age τ_o and birth rate $\lambda_o^* \geq 0$ at age τ_o, choose λ^* as the solution of the following differential equation:

$$\frac{d\lambda^*}{d\tau} = \frac{\tilde{\zeta}}{S^*} + \lambda^* \cdot (\tilde{r} - \lambda^*) \tag{51}$$

with condition $\lambda^*(\tau_o) = \lambda_o^*$, and set $\psi^* := \tilde{\zeta}/\lambda^*$ and $\mu^* := \psi^*/S^* - \psi^*$. The pulled diversification rate of the new scenario $(\lambda^*, \mu^*, \psi^*)$ is given by:

$$\tilde{r}^* = \lambda^* - \mu^* - \psi^* + \frac{1}{\lambda^*}\frac{d\lambda^*}{d\tau} = \lambda^* - \frac{\psi^*}{S^*} + \psi^* - \psi^* + \frac{\tilde{\zeta}}{S^*\lambda^*} + (\tilde{r} - \lambda^*) = \tilde{r}. \tag{52}$$

Further, $\tilde{\zeta}^* = \lambda^*\psi^* = \tilde{\zeta}$. Hence, the new scenario has the same $\tilde{r}$ and the same $\tilde{\zeta}$ as the original scenario. By Supplement S.1.3, the two scenarios are thus congruent. Hence, simply by choosing an alternative S^* and an arbitrary condition $\lambda^*(\tau_o) = \lambda_o^*$ at some age τ_o, one can find a corresponding congruent scenario $(\lambda^*, \mu^*, \psi^*)$, provided that the resulting λ^* and μ^* are physically meaningful (i.e., non-negative). Reciprocally, if the congruence class of a diversification/epidemiological scenario is known (e.g., estimated via maximum-likelihood), and in addition S and $\lambda(\tau_o)$ are somehow independently obtained, then the full scenario can be reconstructed.

S.1.6 The information content of concentrated sampling attempts

In the following we describe how samples obtained during concentrated sampling attempts can yield valuable insight into an epidemic. We define a concentrated sampling attempt (CSA) as a short period of sampling, taking place between two ages $\tau_1 > \tau_2 > 0$, and satisfying the following assumptions:

A. $|\tau_1 - \tau_2|$ is much smaller than $1/\lambda$ and $1/\mu$ throughout the CSA.

B. λ does not vary substantially between the times τ_1 and τ_2, i.e., we assume that $\lambda(\tau_1) \approx \lambda(\tau_2)$.

C. The probability that a lineage alive at τ_1 will be sampled before age τ_2, denoted ρ, is sufficiently high so that during the CSA the number of lineages sampled is much larger than the number of birth and death events, i.e. $\rho \gg \lambda \cdot |\tau_1 - \tau_2|$ and $\rho \gg \mu \cdot |\tau_1 - \tau_2|$.

D. In the exterior vicinity of the open interval (τ_1, τ_2), $\tilde{\lambda}$ and $\tilde{\psi}$ are continuous, so that $\tilde{\lambda}(\tau_1)$, $\tilde{\lambda}(\tau_2)$, $\tilde{\psi}(\tau_1)$ and $\tilde{\psi}(\tau_2)$ can be asymptotically identified.

We will show that in the limit of infinitely large trees, $\lambda(\tau_1)$, $\psi(\tau_1)$ and ρ can be accurately identified. Assuming that the CSA is so short that birth and death are unlikely to occur in any given lineage (assumption A), we have:

$$1 - E(\tau_1) \approx \rho + (1-\rho) \cdot [1 - E(\tau_2)], \tag{53}$$

which is equivalent to:

$$E(\tau_1) \approx E(\tau_2) \cdot (1-\rho). \tag{54}$$

In other words, the probability that a lineage alive at age τ_1 will eventually be sampled is approximately equal to the probability of being sampled during the CSA plus the probability of not being sampled during CSA multiplied by the probability that a lineage alive at age τ_2 will eventually be sampled. In the absence of births and deaths, the above approximation formula would be exact. Assuming that $\lambda(\tau_1) \approx \lambda(\tau_2)$ (assumption B) and using Eq. (54), we have:

$$\begin{aligned}\tilde{\lambda}(\tau_1) =& \lambda(\tau_1) \cdot [1 - E(\tau_1)] \overset{(54)}{\approx} \lambda(\tau_1) \cdot [1 - E(\tau_2)(1-\rho)] \\ =& \lambda(\tau_1) \cdot [1 - E(\tau_2)] + \lambda(\tau_1)\rho E(\tau_2) \overset{B}{\approx} \lambda(\tau_2) \cdot [1 - E(\tau_2)] + \lambda(\tau_1)\rho E(\tau_2) \\ =& \tilde{\lambda}(\tau_2) + \lambda(\tau_1)\rho E(\tau_2).\end{aligned} \tag{55}$$

We mention that, strictly speaking, we only need to assume that $\lambda(\tau_1) \approx \lambda(\tau_2)$, i.e. in principle λ could vary during the CSA as long as it returns to its immediate pre-CSA value. The number of lineages sampled during the age interval $[\tau_1, \tau_2]$, denoted S, can be accurately read off the tree, if the tree is sufficiently large. Assuming that births and deaths within any given lineage are negligible compared to the sampling effort (i.e., changes in the total number of extant lineages are mostly due to sampling, assumption C), and for sufficiently large trees, we have:

$$S \approx N(\tau_1)\rho \approx \frac{M(\tau_1)\rho}{1 - E(\tau_1)}, \tag{56}$$

where N is the total number of extant lineages and M is the tree's LTT. Denoting:

$$\tilde{\rho} := \frac{\rho}{1 - E(\tau_1)}, \tag{57}$$

we see that $\tilde{\rho}$ is asymptotically identifiable, since $\tilde{\rho} \approx S/M(\tau_1)$. Note that:

$$\lambda(\tau_1)\rho = \tilde{\lambda}(\tau_1)\tilde{\rho}. \tag{58}$$

Using Eq. (55) and Eq. (58) we obtain:

$$\tilde{\lambda}(\tau_1) = \tilde{\lambda}(\tau_2) + \tilde{\lambda}(\tau_1)\tilde{\rho}E(\tau_2), \tag{59}$$

and hence:

$$E(\tau_2) = \frac{\tilde{\lambda}(\tau_1) - \tilde{\lambda}(\tau_2)}{\tilde{\rho}\tilde{\lambda}(\tau_1)}. \tag{60}$$

Since $\tilde{\lambda}(\tau_1)$ and $\tilde{\lambda}(\tau_2)$ are asymptotically identifiable (assumption D), and $\tilde{\rho}$ is also asymptotically identifiable, it follows that $E(\tau_2)$ is also asymptotically identifiable. Note that the condition $\tau_2 > 0$ is necessary for ensuring that $\tilde{\lambda}(\tau_2)$ is asymptotically identifiable, since we need samples younger than τ_2 for estimating $\tilde{\lambda}(\tau_2)$. Solving Eq. (54) and Eq. (57) for ρ and $E(\tau_1)$ leads to:

$$\rho \approx \frac{\tilde{\rho} \cdot [1 - E(\tau_2)]}{1 - \tilde{\rho} E(\tau_2)}. \tag{61}$$

Hence, ρ is also asymptotically identifiable. Combined with Eq. (54), this implies that $E(\tau_1)$ is also asymptotically identifiable. Solving Eq. (58) for $\lambda(\tau_1)$ leads to:

$$\lambda(\tau_1) \approx \frac{\tilde{\lambda}(\tau_1)\tilde{\rho}}{\rho}. \tag{62}$$

Hence, $\lambda(\tau_1)$ is also asymptotically identifiable. Lastly, since $\tilde{\psi}(\tau_1)$ and $\tilde{\psi}(\tau_2)$ are asymptotically identifiable (assumption D), and since $\psi = \tilde{\psi} \cdot (1 - E)$, we conclude that $\psi(\tau_1)$ and $\psi(\tau_2)$ are also asymptotically identifiable.

□

S.1.7 Infinitesimally short concentrated sampling attempts

From a numerical perspective, it might be more practical to describe very short CSAs as separate instantaneous sampling processes with designated parameters (e.g., the time of the CSA and the probability of sampling a lineage during the CSA), rather than trying to approximate CSAs as very sharp peaks in ψ. The resulting model structure would then resemble the one described previously by Stadler *et al.* [2]. This is also the approach taken in the R package castor when fitting BDS models to timetrees: CSAs are parameterized separately from Poissonian sampling [4]. Formally, one can derive the corresponding likelihood starting from the BDS likelihood introduced earlier (Eqs. (1) or (4)) by considering a modified sampling rate of the form:

$$\overline{\psi} = \psi + \sum_{k=1}^{m} w_k \delta(\tau - s_k), \tag{63}$$

where δ is the Dirac distribution, m is the number of CSAs, s_k is the age of the k-th CSA ($0 \leq s_1 < .. < s_m \leq \tau_{\text{or}}$), $w_k := -\ln(1-\rho_k)$ is a weight, $\rho_k \in (0, 1]$ is the sampling probability during the k-th CSA and ψ is the "background" (Poissonian) sampling rate outside of the CSAs. For a derivation of the corresponding likelihood of a bifurcating timetree in terms of λ, μ, ψ, the s_k and the ρ_k see [1]. For convenience, we provide the likelihood below using the notation of the present manuscript. We denote by N_k the number of tips sampled during and due to the k-th CSA, and by n_k the number of lineages "crossing" over s_k in the timetree. The likelihood density of the tree, when conditioned on the age of the origin τ_{or} and the survival of at least one lineage, is then given by:

$$L_{\text{or}} = \frac{\Phi_m(\tau_{\text{or}})}{1 - E(\tau_{\text{or}})} \prod_{i=1}^{n-1} \lambda(x_i)\Phi_{m_{x_i}}(x_i) \prod_{k=1}^{m} [(1 - \rho_k)\Phi_{k-1}(s_k)]^{n_k} \rho_k^{N_k} \prod_{j=1}^{n} \frac{\psi(y_j)}{\Phi_{m_{y_j}}(y_j)}, \tag{64}$$

where as before $E(\tau)$ is the probability that a lineage alive at age τ is not included in the timetree, m_τ is the largest possible integer for which $s_{m_\tau} \leq \tau$, and $\Phi_0, .., \Phi_m$ are the sub-flows [3] between CSAs, defined as:

$$\Phi_k(\tau) := \exp\left[\int_{s_k}^{\tau} [2\lambda(s)E(s) - \lambda(s) - \mu(s) - \psi(s)]\ ds\right], \quad k = 0, .., m, \tag{65}$$

where for notational simplicity we defined $s_0 := 0$. Note that for each $k = 0, .., m$ the probability E satisfies within the age interval $[s_k, s_{k+1})$ the differential equation:

$$\frac{dE}{d\tau} = -(\lambda + \mu + \psi)E + \lambda E^2 + \mu, \tag{66}$$

with initial condition $E(s_k) = E(s_k^-) \cdot (1 - \rho_k)$ and $E(0^-) = 1$. Similarly, one can derive the likelihood of a bifurcating timetree conditioned on the age of the tree's root τ_r, i.e., a splitting occurring at age τ_r and the eventual sampling of both child lineages:

$$L_\mathrm{r} = \frac{\Phi_m(\tau_\mathrm{r})}{\lambda(\tau_\mathrm{r}) \cdot (1 - E(\tau_\mathrm{r}))^2} \prod_{i=1}^{n-1} \lambda(x_i)\Phi_{m_{x_i}}(x_i) \prod_{k=1}^{m} [(1 - \rho_k)\Phi_{k-1}(s_k)]^{n_k}\, \rho_k^{N_k} \prod_{j=1}^{n} \frac{\psi(y_j)}{\Phi_{m_{y_j}}(y_j)}. \tag{67}$$

S.2 Why common model selection methods cannot resolve congruencies

In this section we clarify why model selection methods that do not integrate additional independent information and are entirely based on parsimony principles, such as AIC [5], BIC [6] or regularization [7], are not suitable for resolving model congruencies.

S.2.1 General considerations (big picture)

Parsimony-based model selection methods such as AIC, BIC and regularization essentially penalize excessive model complexity and are designed to prevent overfitting to finite datasets, i.e., to avoid inferring spurious complexities when the data at hand can also be adequately explained by simpler models. Indeed, it is common for multiple models to explain a dataset similarly well and in this case it is prudent to select the model with fewer free parameters since this tends to decrease the prediction error. These approaches do not, by any means, guarantee or even suggest that for any given dataset the selected (simpler) model is the one closest to the truth, as is often erroneously assumed. These methods merely constitute a methodological principle for gradually and conservatively choosing more complex models as new data appear, under the presumption that as new data accumulate the selected models will eventually converge towards the truth.

When modelling pathogen epidemics, however, one must decide between congruent scenarios with differing levels of complexity, and no phylogenetic data, no matter how large, can distinguish between their proximity to the truth. In fact, all scenarios within a congruence class generate phylogenies with the same probability distribution, and hence they also have identical expected "prediction errors" (which, for example, AIC was designed to minimize). Hence, merely selecting the simpler model will almost always lead to an erroneous scenario, no matter how much sequence data we add. The very promise of conventional parsimony-based model selection — that selected models eventually converge to the truth as one adds more data — simply does not hold in this case.

One may of course compare scenarios belonging to different congruence classes since these will generally

have different likelihoods for a particular dataset, and will certainly be distinguishable as the size of the dataset becomes very large. However, this does not solve the problem at hand, since one is not really selecting the best model for the data but in fact merely the best congruence class. Even if one of the candidate scenarios in the candidate set is entirely adequate for explaining the data at hand, or in the extreme case, is in the exact same congruence class as the true historical epidemiological dynamics, it does not follow that this scenario will resemble, even qualitatively, the true historical dynamics.

In conclusion, model selection methods that do not integrate additional independent information can at most be used to find a congruence class that "most efficiently" reproduces the data, or that has the lowest prediction error. In that sense, fitting congruence classes (e.g., by directly fitting pulled variables) is a parsimonious approach towards describing a phylogenetic dataset, while fitting full birth-death-sampling models adds redundant complexity that cannot possibly be identified using phylogenetic inference alone.

S.2.2 Regularization

Let us consider specifically the example of fitting piecewise linear (or piecewise constant) profiles for λ, μ and ψ on a grid over time, combined with a regularization approach that penalizes excessive oscillations or excessively large rate estimates (an example being Tikhonov regularization [8, 9]). To see why such an approach cannot possibly resolve model congruencies, suppose that a specific epidemiological history occurred, and consider the hypothetical limit where a phylogeny generated by that epidemic becomes infinitely large while the considered resolution of the fitted profiles (i.e., the number of grid points) remains fixed. In the absence of model congruencies, i.e., in typical inverse problems where regularization is commonly applied, one would expect that the fitted profiles would eventually approach the true historical profiles, provided of course that the grid resolution is high enough to approximately capture the true historical profiles. But this is clearly not the case here. Indeed, in the limit of an infinitely large phylogeny regularization becomes irrelevant, since for any given regularization parameter (also known as “smoothing parameter”) minimizing deviations from the data becomes infinitely more important than reducing the regularization penalties; in other words, for very large trees regularized profile fitting becomes equivalent to maximum-likelihood fitting. As we have demonstrated using simulations (Supplemental Figs. S1 and S2), maximum-likelihood-fitted profiles can be completely wrong even when using massive phylogenies with tens of thousands of tips and for relatively simple epidemiological histories. The situation can only be worse for smaller datasets.

S.2.3 AIC and BIC

Alternatively, let us consider the example of fitting various functional forms for λ, μ and ψ via maximum-likelihood, with the “best” functional form being selected using AIC [5] or BIC [6]. For any given choice of functional forms, the maximum-likelihood-fitted model will a priori tend to be the one closest to the congruence class of the true epidemiological history, rather than the true epidemiological history itself. Choosing the functional forms that minimize AIC or BIC would only yield a model that balances the number of parameters against the goodness of fit to the congruence class, but not against the goodness of fit to the true epidemiological history. There is little reason to expect that the fitted model selected via AIC or BIC will happen to actually be close to the true history, even for massive datasets (examples in Supplemental Figs. S1 and S2, as well as in Supplement S.3). The situation can only be worse for small datasets.

S.3 Statistical evaluation of multiple simulated scenarios

In the following we describe our statistical analysis of models fitted to multiple trees from simulated epidemiological BDS scenarios (overview of results in Supplemental Figs. S17, S19, S18, S20). All rates are given in units yr^{-1}. All simulations were run for 10 years, and only those that generated trees with 500–50 000 tips were kept. We considered two different classes of BDS scenarios. In the first class (henceforth "exponential"), λ (and similarly μ and ψ) was of the general form:

$$\lambda(t) = A + B \cdot e^{\alpha t}, \tag{68}$$

where t is time and A, B and α are parameters chosen randomly as follows. Denote $t_{\mathrm{s}} = 0$ the start and $t_{\mathrm{e}} = 10$ yr the end of a simulation. We randomly and uniformly chose $\lambda(t_{\mathrm{s}})$ and $\lambda(t_{\mathrm{e}})$ between 1 and 10, and randomly and uniformly chose α in $[-0.5, -0.1] \cup [0.1, 0.5]$. Based on these three constraints, we algebraically determined the appropriate A and B. A similar approach was taken for constructing μ and ψ, with the difference that we randomly and uniformly chose each of the ratios $\mu(t_{\mathrm{s}})/\lambda(t_{\mathrm{s}})$ and $\mu(t_{\mathrm{e}})\lambda(t_{\mathrm{e}})$ between 0.1 and 1, and randomly chose each of $\psi(t_{\mathrm{s}})$ and $\psi(t_{\mathrm{e}})$ between 0.01 and 1 (uniformly on a logarithmic scale). For any particular choice of λ, μ and ψ, we simulated a tree for 10 years using the function `generate_tree_hbds` in the R package `castor` v1.6.7 [4]. If the resulting tree had too few tips (<500) or too many tips (>50 000), we chose an entirely new set of λ, μ and ψ and repeated the simulation until obtaining an acceptable tree. In the 2nd class (henceforth "OU") the time-profile for λ (and similarly μ and ψ) was generated according to an Ornstein-Uhlenbeck stochastic process [10]. This approach was chosen in order to cover a wide range of epidemiological scenarios with realistic temporal complexity. An Ornstein-Uhlenbeck process is characterized by its stationary or asymptotic expectation (λ_{ex}), its stationary or asymptotic standard deviation (λ_{dev}) and its decay rate α. We chose the decay rate randomly and uniformly between 0.05 and 0.2, the stationary expectation λ_{ex} randomly and uniformly between 1 and 10, and fixed the stationary standard deviation to $\lambda_{\mathrm{dev}} = 0.5 \cdot \lambda_{\mathrm{ex}}$. The actual profile λ was then generated randomly according to the Ornstein-Uhlenbeck process evaluated at 6-month intervals, restricted (capped) to above $0.1 \cdot \lambda_{\mathrm{ex}}$, and linearly interpolated between points. A similar approach was taken for constructing μ and ψ, with the difference that for μ we chose the stationary expectation μ_{ex} randomly and uniformly between $0.1 \cdot \lambda_{\mathrm{ex}}$ and λ_{ex}, and for ψ we chose the stationary expectation ψ_{ex} randomly between 0.01 and 1 (uniformly on a logarithmic scale). Similarly to the exponential scenarios, we simulated a tree for 10 years under a OU scenario using the function `generate_tree_hbds`, and repeated the process until a tree comprising between 500 and 50000 tips was obtained. In total we generated 100 trees from exponential scenarios and 100 trees from OU scenarios.

To each generated tree we fitted a BDS skyline model (i.e., with piecewise constant λ, μ and ψ) and a BDS model with piecewise-linear λ, μ and ψ, via maximum-likelihood using the `castor` function `fit_hbds_model_on_grid`. Fitting was done agnostically of the true scenario used in the simulations, except for the constraint that the present-day ψ was fixed to its true value to account for previously known identifiability issues in BDS skyline models [2]. To avoid local non-global likelihood maxima, we repeated the fitting using 100 random starting points (option `Ntrials=100`). The grid points were placed non-uniformly so that their density was roughly proportional to the square root of the tree's LTT, using the `castor` function `get_inhomogeneous_grid_1D`; this was done so that points tend to be concentrated in regions with higher information content. The best grid size was chosen according to the AIC [5]. Since in almost all cases the best fitted piecewise linear model had a better (lower) AIC value than the best fitted skyline model, we henceforth focused on piecewise linear models for simplicity. To avoid erroneous parameter estimates stemming from bad model fits, we focused on those trees where the best fitted piecewise linear model (henceforth simply "fitted model") adequately explained the input tree, based on the same Kolmogorov-Smirnov tests for the node ages, tip ages and edge lengths described in the main article (implemented in the `castor` function

model_adequacy_hbds). In total we thus considered 92 exponential and 66 OU simulations with adequate model fits.

For every considered tree and every parameter (such as λ), we calculated the coefficient of determination between the true profile and the fitted profile, R^2, as follows:

$$R^2 = 1 - \frac{\sum_{i=1}^{N}(\lambda(t_i) - \hat{\lambda}(t_i))^2}{N \cdot \mathbb{V}(\lambda)}, \tag{69}$$

where $t_1, .., t_N$ is a fine time grid, λ is the true birth rate, $\hat{\lambda}$ is the estimated birth rate, and $\mathbb{V}(\lambda)$ is the variance of the true birth rate (evaluated on the same time grid t_1,..,t_N). Hence, a negative R^2 corresponds to a worse model fit than if one just used the mean as a prediction. We calculated the mean modulus of the normalized error (MMNE) as follows:

$$\text{MMNE} = \frac{1}{N}\sum_{i=1}^{N}\frac{\left|\lambda(t_i) - \hat{\lambda}(t_i)\right|}{\mathbb{E}(\lambda)}, \tag{70}$$

where $\mathbb{E}(\lambda)$ is the mean value of the true birth rate. To calculate the linear trend of a model parameter (such as λ) over time, we used least-squares linear regression with an intercept, applied to the true or estimated profile of the parameter. The R^2, MMNE and true and estimated linear trends of selected model parameters for all considered simulations are shown in Supplemental Figs. S17 (exponential) and S19 (OU). Specific examples of fitted R_e are shown in Supplemental Figs. S18 and S20. As can be seen, the vast majority of fitted models poorly reconstructed the true epidemiological scenario, including in particular the R_e. However, the fitted models did generally come close to the true scenario's congruence class, as seen from the accurately reconstructed deterministic branching and sampling densities ($\tilde{\beta}$ and $\tilde{\sigma}$).

S.4 Overview of computer code

This section provides an overview of computational tools developed as part of this manuscript, and publicly available in the R package castor v1.6.5 [4], for working with BDS models and congruencies. The functions described below have been designed with efficiency in mind, and can typically scale well to phylogenies with hundreds of thousands of tips. For detailed instructions consult the castor user manual.

generate_tree_hbds: Generate a random timetree according to a time-dependent BDS model with arbitrary rates λ, μ, ψ through time. The rates are specified as piecewise linear curves (or quadratic splines, or cubic splines) on a discrete time grid. By choosing a sufficiently fine time grid, any arbitrary functional forms can in principle be accommodated to arbitrary accuracy.

simulate_deterministic_hbds: Simulate a time-dependent BDS model with arbitrary rates λ, μ, ψ through time, in the deterministic limit (i.e., use differential equations rather than a stochastic process). This function can be used to calculate various alternative parameters of a BDS model, such as the R_e, the removal rate δ, the sampling proportion S, the LTT, the pulled birth rate $\tilde{\lambda}$ and so on. In particular, this function can be used to check if two BDS models are congruent, by comparing their pulled birth rate ($\tilde{\lambda}$) and event density ($\lambda\psi$).

fit_hbds_model_on_grid: Fit a time-dependent BDS model to a timetree via maximum-likelihood. The λ, μ and ψ can be assumed to be either piecewise constant, piecewise linear, quadratic splines or cubic splines

defined on a discrete time grid; the values of these parameters are thus fitted at each grid point. This function uses parametric bootstrapping to calculate confidence intervals in the estimated parameters.

fit_hbds_model_parametric: Fit a BDS model to a timetree via maximum-likelihood. The profiles of λ, μ and ψ are described by user-specified functions that depend on a finite number of scalar parameters to be fitted. This function can thus be used to fit arbitrary functional forms. The function uses parametric bootstrapping to calculate confidence intervals in the estimated parameters.

congruent_hbds_model: Construct new BDS models by providing information on the congruence class (such as the pulled birth rate) and any additional constraints needed for identifying a specific member of the congruence class. This function can be used to explore the set of models congruent to some reference model. It was used, for example, to generate the scenarios in Fig. 1 in the main article.

model_adequacy_hbds: Test if a given BDS model (or a distribution of BDS models) adequately describes a given timetree in terms of various statistics. For example, this function can perform a Kolmogorov-Smirnov test to examine if the distribution of node ages (or tip ages, or edge lengths) in the timetree deviate significantly from those expected under the model(s). Note that, due to the existence of model congruencies, even if a BDS model adequately describes a given timetree this does not mean that the model is even close to the original BDS scenario that generated the tree. If a BDS model adequately describes a timetree, it merely means that any of the myriad of members of the model's congruence class would generate trees similar to the tree at hand.

S.5 On the use of occurrence data with phylogenies

While additional data is generally useful for increasing estimation accuracy, we would like to clarify our thoughts on the potential use of "occurrence" data, commonly encountered in epidemiology, in combination with molecular/phylogenetic data, for resolving the identifiability issues uncovered in this paper. Specifically, we caution that the addition of occurrence data, i.e., reports of positive cases detected at specific times without sequencing [11–13], need not necessarily resolve congruencies between epidemiological scenarios, if the rate at which such detections occur (denoted ϑ) is a priori unknown and time-dependent. The reason is that such data contain a strict subset of the information that would be contained in detected cases with sequences (and thus included in the phylogeny), and hence phylogenetic data combined with occurrence data can at most yield as much information as a larger phylogenetic dataset in which each detected case is also sequenced. As we have shown in this paper, in the absence of further information or constraints, the rates λ, μ and ψ cannot be reconstructed from phylogenetic data alone. Thus, in the absence of further information or constraints, λ, μ, ψ, ϑ or $\psi + \vartheta$ cannot possibly be reconstructed using phylogenetic and occurrence data alone.

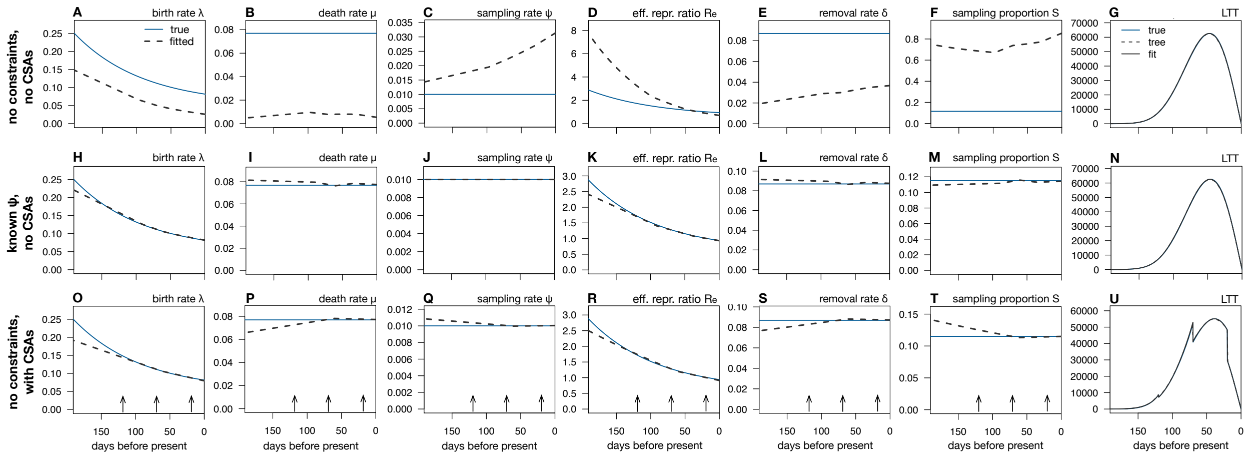

Figure S1: **Limits to reconstructing an epidemic's dynamics via maximum-likelihood (scenario 1).** (A–F) Maximum-likelihood estimates (grey dashed curves) of the birth rate (λ), death rate (μ), sampling rate (ψ), effective reproduction ratio ($R_{\rm e}$), removal rate ($\delta = \mu + \psi$) and sampling proportion ($S = \psi/(\mu+\psi)$) over time, based on a timetree with 175,440 tips simulated under a hypothetical birth-death-sampling scenario (blue continuous curves) and without any additional constraints. All rates are in day^{-1}. Model adequacy was confirmed using predictive posterior simulations with multiple tests. Observe the poor agreement between the estimated and true profiles. (G) Deterministic LTT (dLTT) of the fitted model, compared to the true scenario's dLTT and the LTT of the timetree (note that all curves are nearly identical). (H–N) Similar to A–G, but for a model fitted to the same data as in A–G while fixing the sampling rate to its true profile. Observe the improved agreement between estimated and true profiles. (O–U) BDS parameters and dLTT for a model fitted to a timetree with 172,888 tips, generated under nearly the same BDS scenario as in A–G but with 3 added concentrated sampling attempts (CSAs, times indicated by vertical arrows). No additional constraints were used during fitting.

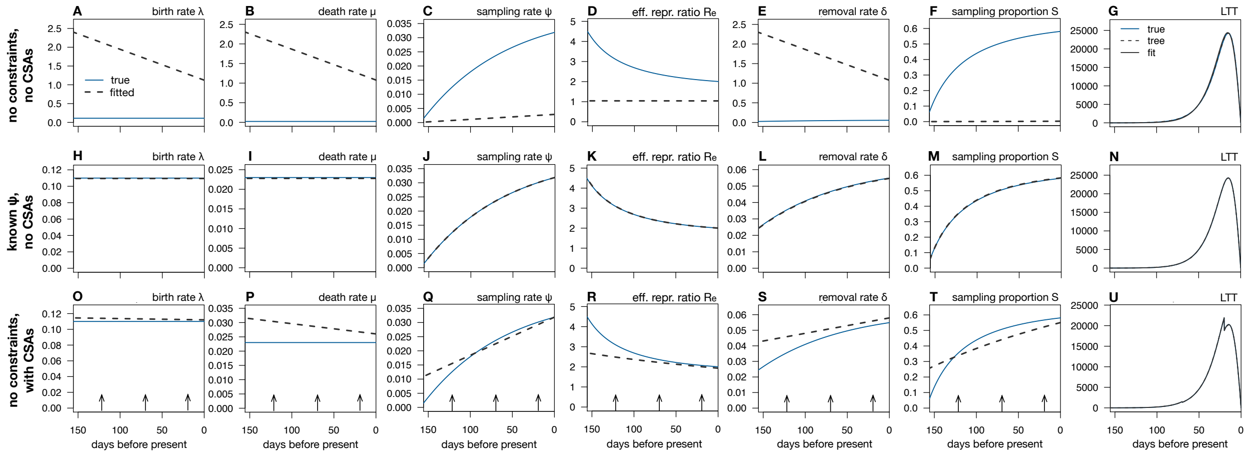

Figure S2: **Limits to reconstructing an epidemic's dynamics via maximum-likelihood (scenario 2).** (A–F) Maximum-likelihood estimates (grey dashed curves) of the birth rate (λ), death rate (μ), sampling rate (ψ), effective reproduction ratio (R_{e}), removal rate ($\delta = \mu + \psi$) and sampling proportion ($S = \psi/(\mu + \psi)$) over time, based on a timetree with 55,934 tips simulated under a hypothetical birth-death-sampling scenario (blue continuous curves) and without any additional constraints. All rates are in day^{-1}. Model adequacy was confirmed using predictive posterior simulations with multiple tests. Observe the poor agreement between the estimated and true profiles. (G) Deterministic LTT (dLTT) of the fitted model, compared to the true scenario's dLTT and the LTT of the timetree (note that all curves are nearly identical). (H–N) Similar to A–G, but for a model fitted to the same data as in A–G while fixing the sampling rate to its true profile. Observe the improved agreement between estimated and true profiles. (O–U) BDS parameters and dLTT for a model fitted to a timetree with 51,619 tips, generated under nearly the same BDS scenario as in A–G but with 3 added concentrated sampling attempts (CSAs, times indicated by vertical arrows). No additional constraints were used during fitting.

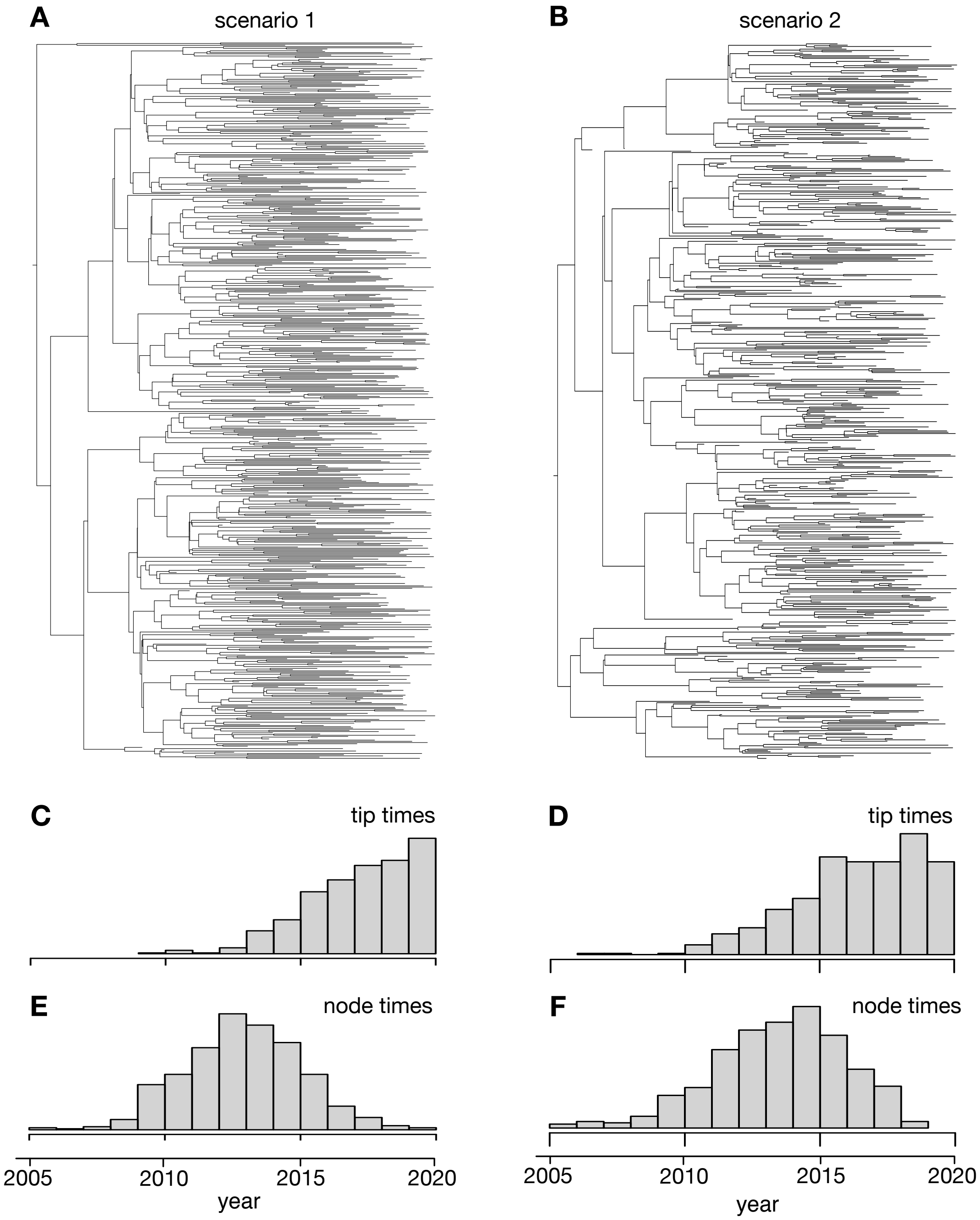

Figure S3: **Phylogenetic trees simulated for the BEAST analysis.** (A–B) Phylogenetic trees simulated under two hypothetical epidemiological scenarios, for Bayesian inference. (C, E) Histograms of tip ages and node ages in tree A (bar heights are in arbitrary units). (D, F) Histograms of tip ages and node ages in tree B (bar heights are in arbitrary units).

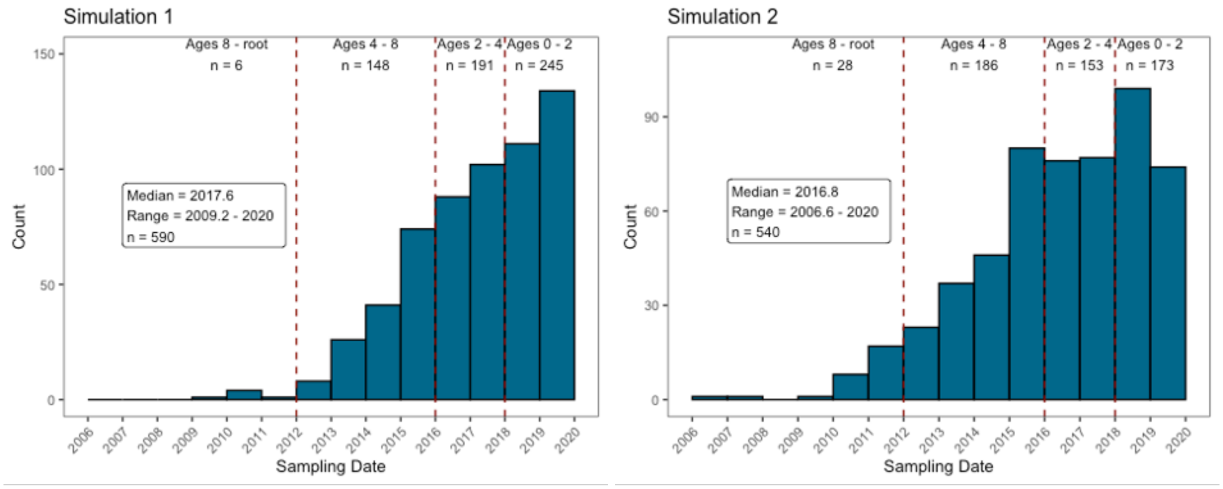

Figure S4: **Choosing rate shift times for the BEAST simulation analysis.** The distribution of sampling dates in the simulated trees informed the timing of specified rate shifts in fitted skyline models, to partially equalize the number of samples informing each interval and to ensure that the earliest interval had greater than zero samples.

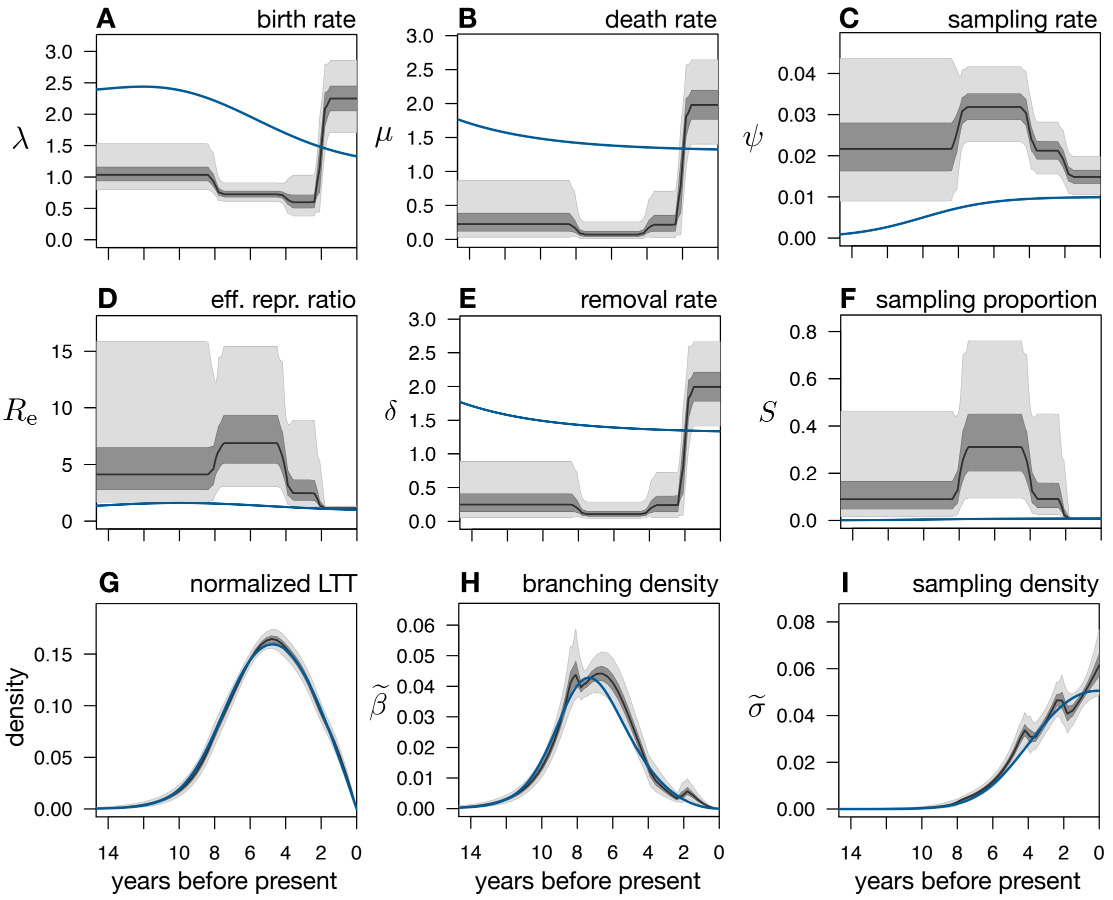

Figure S5: **Reconstructing an epidemic's dynamics in a Bayesian framework (BEAST2 run U1).** (A–F) Posterior distributions of the birth rate (λ), death rate (μ), sampling rate (ψ), effective reproduction ratio (R_e), removal rate ($\delta = \mu + \psi$) and sampling proportion ($S = \psi/(\mu + \psi)$), as inferred from 590 sequences simulated under a hypothetical birth-death-sampling scenario (blue curves) using BEAST2 (tree in Supplemental Fig. S3A). Black curves show posterior median, dark and light shades represent equal-tailed 50%- and 95%-credible intervals of the posterior. All rates are in yr^{-1}. The present-day sampling proportion was fixed to its true value during fitting, to account for previously reported identifiability issues [2]. Model adequacy was confirmed using predictive posterior simulations with multiple test statistics. Note the poor agreement between the predicted and true profiles. (G–I) Distributions of the deterministic lineages-through-time curves (normalized to unit area under the curve), branching densities ($\tilde{\lambda}$) and sampling densities ($\tilde{\psi}$), corresponding to the same posterior scenarios as in A–F, compared to their true profiled (blue curves). The relatively good agreement between the inferred and true profiles shows that BEAST2 closely reconstructed the epidemiological history's congruence class but not the epidemiological history itself. See Supplemental Fig. S10 for the corresponding posterior distributions of the molecular evolution parameters.

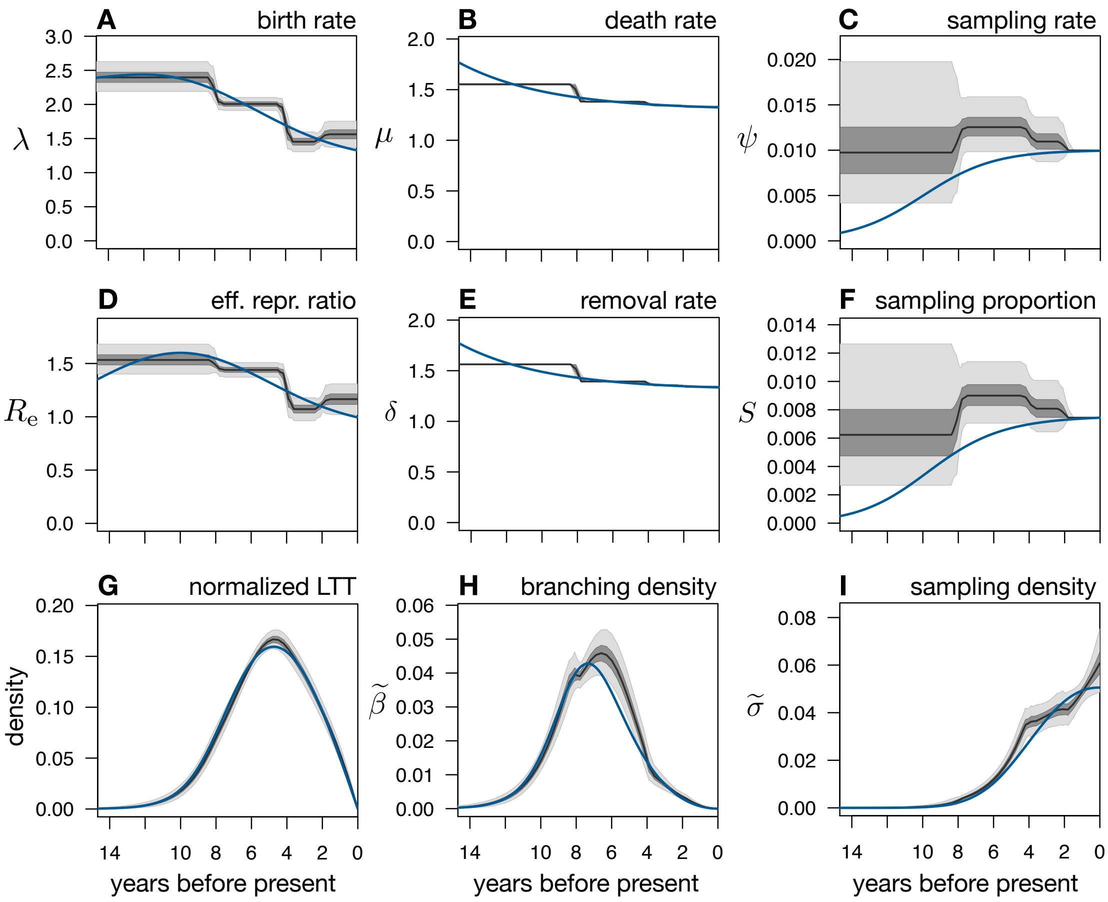

Figure S6: **Reconstructing an epidemic's dynamics in a Bayesian framework (BEAST2 run F1).** (A–F) Posterior distributions of the birth rate (λ), death rate (μ), sampling rate (ψ), effective reproduction ratio (R_e), removal rate ($\delta = \mu+\psi$) and sampling proportion ($S = \psi/(\mu+\psi)$), as inferred from 590 sequences simulated under a hypothetical birth-death-sampling scenario (blue curves) using BEAST2 (tree in Supplemental Fig. S3A). Black curves show posterior median, dark and light shades represent equal-tailed 50%- and 95%-credible intervals of the posterior. All rates are in yr^{-1}. The present-day sampling proportion as well as the removal rate (all intervals) were fixed to their true values during fitting. Model adequacy was confirmed using predictive posterior simulations with multiple test statistics. Note the much better agreement with the true profiles, compared to the situation where the removal rate was not fixed (Supplemental Fig. S5). (G–I) Distributions of the deterministic lineages-through-time curves (normalized to unit area under the curve), branching densities ($\tilde{\lambda}$) and sampling densities ($\tilde{\psi}$), corresponding to the same posterior scenarios as in A–F, compared to their true profiled (blue curves). See Supplemental Fig. S11 for the corresponding posterior distributions of the molecular evolution parameters.

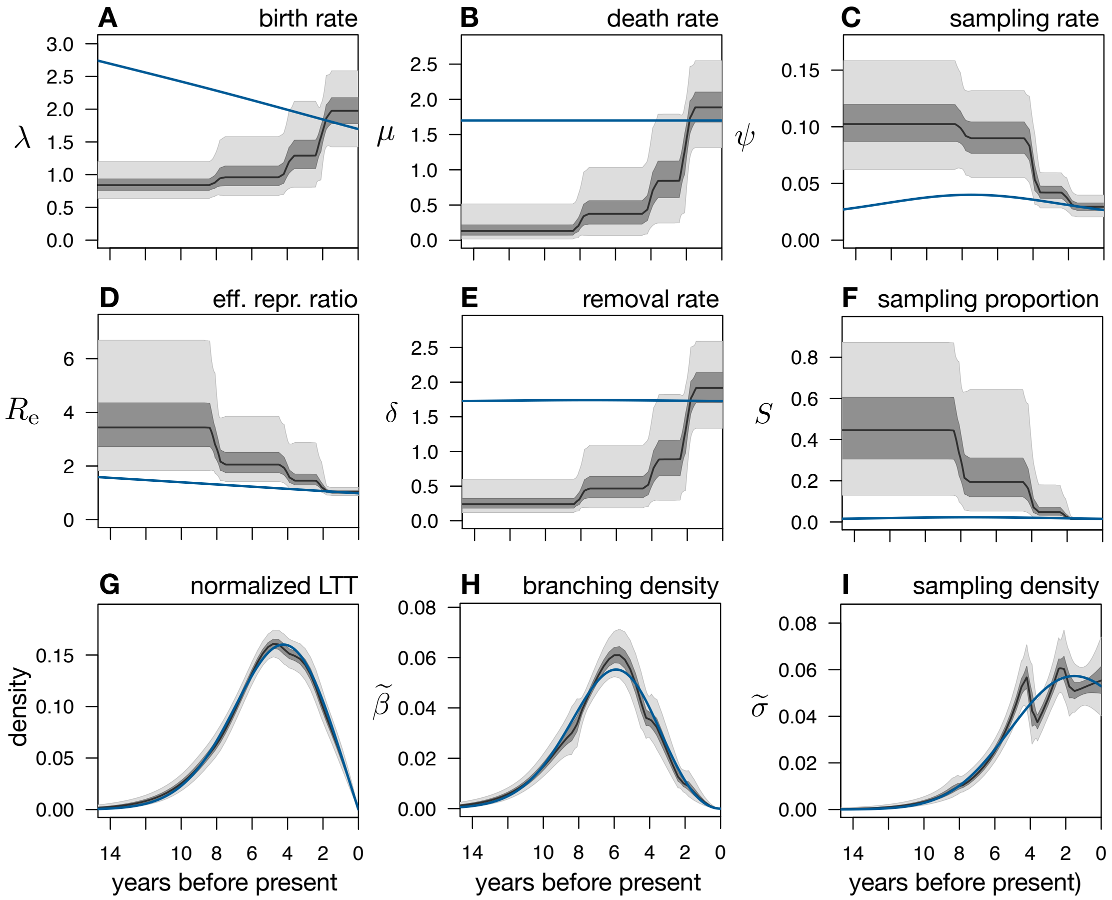

Figure S7: **Reconstructing an epidemic's dynamics in a Bayesian framework (BEAST2 run U2).** (A–F) Posterior distributions of the birth rate (λ), death rate (μ), sampling rate (ψ), effective reproduction ratio (R_e), removal rate ($\delta = \mu+\psi$) and sampling proportion ($S = \psi/(\mu+\psi)$), as inferred from 540 sequences simulated under a hypothetical birth-death-sampling scenario (blue curves) using BEAST2 (tree in Supplemental Fig. S3B). Black curves show posterior median, dark and light shades represent equal-tailed 50%- and 95%-credible intervals of the posterior. All rates are in yr^{-1}. The present-day sampling proportion was fixed to its true value during fitting, to account for previously reported identifiability issues [2]. Model adequacy was confirmed using predictive posterior simulations with multiple test statistics. Note the poor agreement between the predicted and true profiles. (G–I) Distributions of the deterministic lineages-through-time curves, branching densities ($\tilde{\lambda}$) and sampling densities ($\tilde{\psi}$), corresponding to the same posterior scenarios as in A–F, compared to their true profiled (blue curves). The relatively good agreement between the inferred and true profiles shows that BEAST2 closely reconstructed the epidemiological history's congruence class but not the epidemiological history itself. See Supplemental Fig. S12 for the corresponding posterior distributions of the molecular evolution parameters.

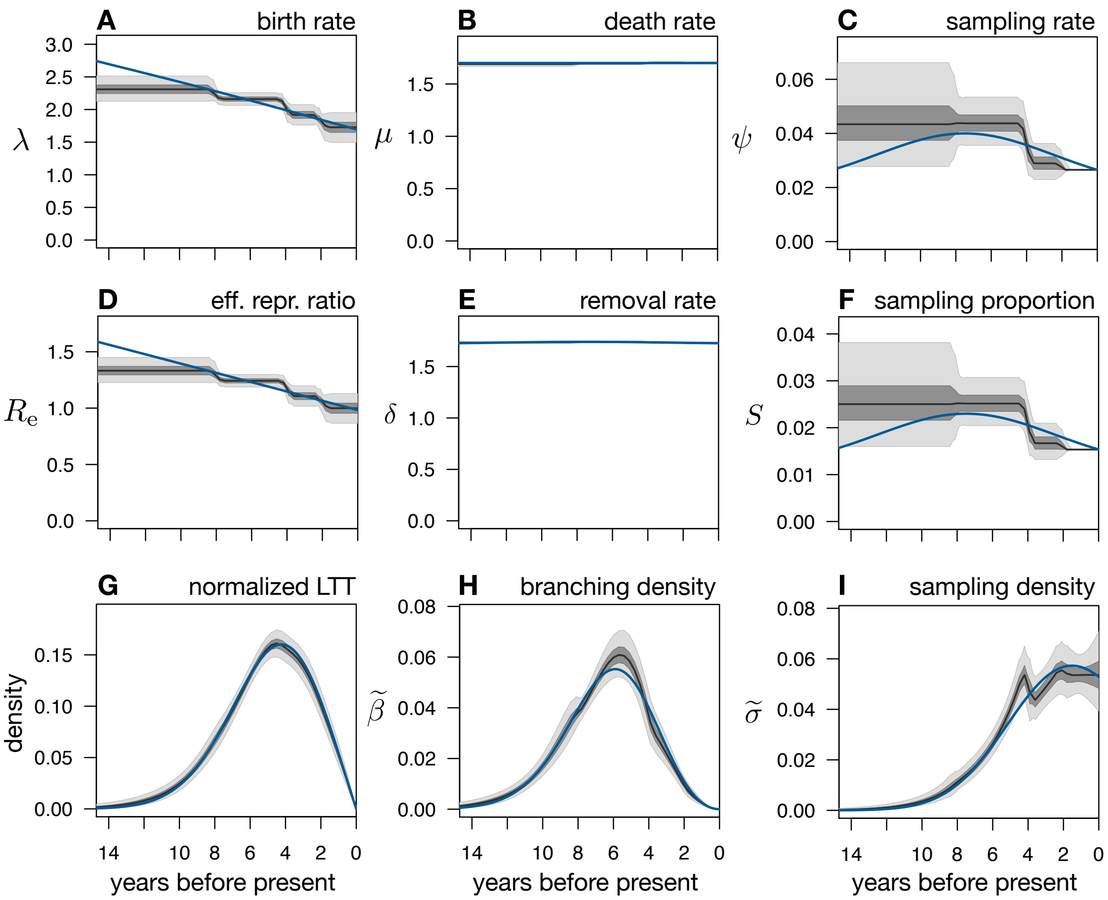

Figure S8: **Reconstructing an epidemic's dynamics in a Bayesian framework (BEAST2 run F2).** (A–F) Posterior distributions of the birth rate (λ), death rate (μ), sampling rate (ψ), effective reproduction ratio ($R_{\rm e}$), removal rate ($\delta = \mu+\psi$) and sampling proportion ($S = \psi/(\mu+\psi)$), as inferred from 540 sequences simulated under a hypothetical birth-death-sampling scenario (blue curves) using BEAST2 (tree in Supplemental Fig. S3B). Black curves show posterior median, dark and light shades represent equal-tailed 50%- and 95%-credible intervals of the posterior. All rates are in yr^{-1}. The present-day sampling proportion and the removal rate (all time intervals) were fixed to their true value during fitting. Model adequacy was confirmed using predictive posterior simulations with multiple test statistics. Note the good agreement between the predicted and true profiles, made possible by fixing one of the model's parameters. (G–I) Distributions of the deterministic lineages-through-time curves (normalized to unit area under the curve), branching densities ($\tilde{\lambda}$) and sampling densities ($\tilde{\psi}$), corresponding to the same posterior scenarios as in A–F, compared to their true profiled (blue curves). See Supplemental Fig. S14 for the corresponding posterior distributions of the molecular evolution parameters.

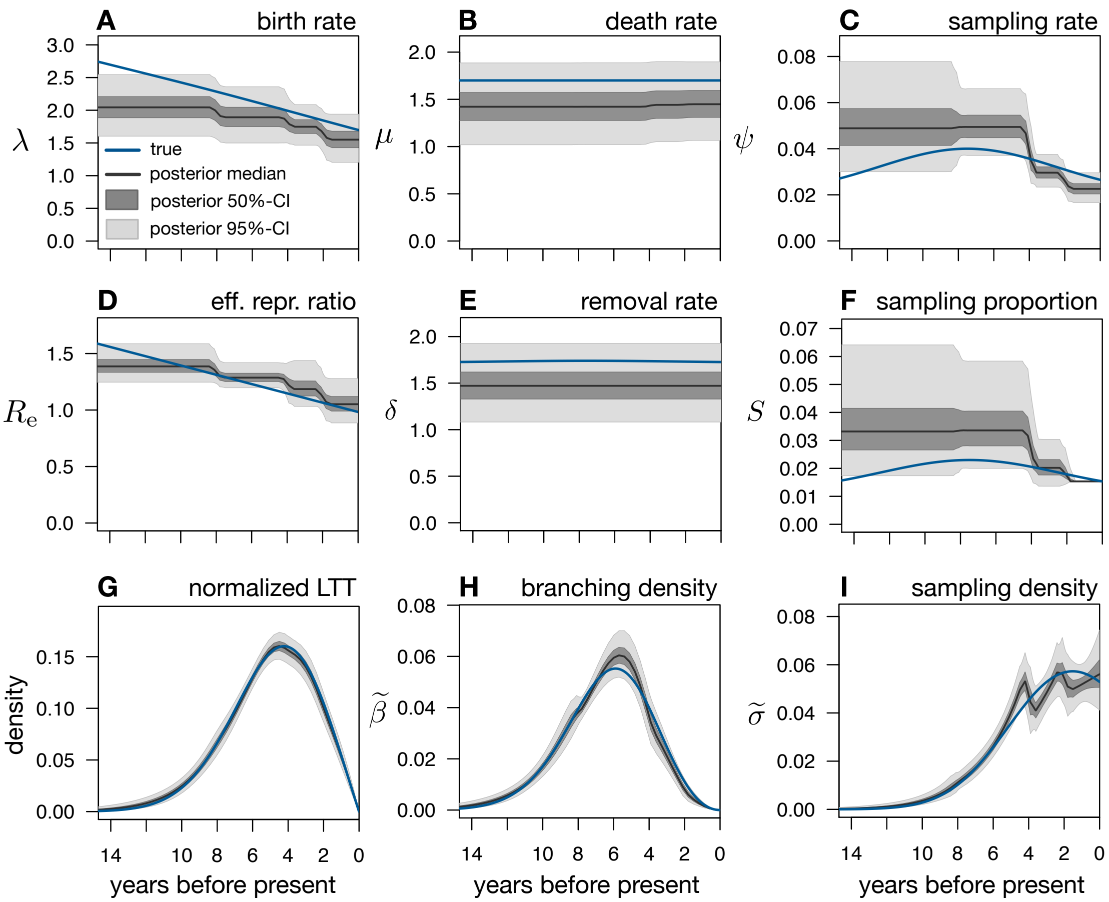

Figure S9: **Reconstructing an epidemic's dynamics in a Bayesian framework (BEAST2 run C2).** (A–F) Posterior distributions of the birth rate (λ), death rate (μ), sampling rate (ψ), effective reproduction ratio (R_e), removal rate ($\delta = \mu + \psi$) and sampling proportion ($S = \psi/(\mu + \psi)$), as inferred from 540 sequences simulated under a hypothetical birth-death-sampling scenario (blue curves) using BEAST2 (tree in Supplemental Fig. S3B). Black curves show posterior median, dark and light shades represent equal-tailed 50%- and 95%-credible intervals of the posterior. All rates are in yr^{-1}. The removal rate was constrained to be constant over time. The present-day sampling proportion was fixed to its true value during fitting, to account for previously reported identifiability issues [2]. Model adequacy was confirmed using predictive posterior simulations with multiple test statistics. (G–I) Distributions of the deterministic lineages-through-time curves (normalized to unit area under the curve), branching densities ($\tilde{\lambda}$) and sampling densities ($\tilde{\psi}$), corresponding to the same posterior scenarios as in A–F, compared to their true profiled (blue curves). See Supplemental Fig. S13 for the corresponding posterior distributions of the molecular evolution parameters.

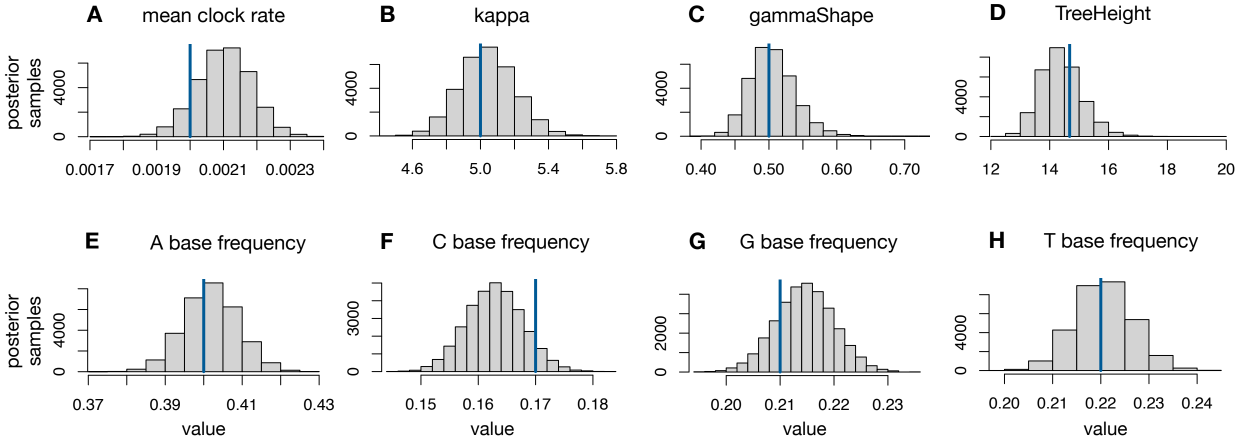

Figure S10: **Posterior distributions of molecular evolution parameters (BEAST2 run U1).** Posterior distributions of (A) the mean molecular clock rate, (B) the transition/transversion ratio κ, (C) the shape parameter of the discretized gamma distribution of substitution rates, (D) the height of the tree or root age, and (E–H) the stationary frequencies of nucleotide bases A,C,G,T, as inferred from 590 sequences simulated under a hypothetical birth-death-sampling scenario using BEAST2 (epidemiological parameters in Supplemental Fig. S5, tree in Supplemental Fig. S3A). Histogram bars show frequencies. Blue vertical lines show the true values used in the simulation.

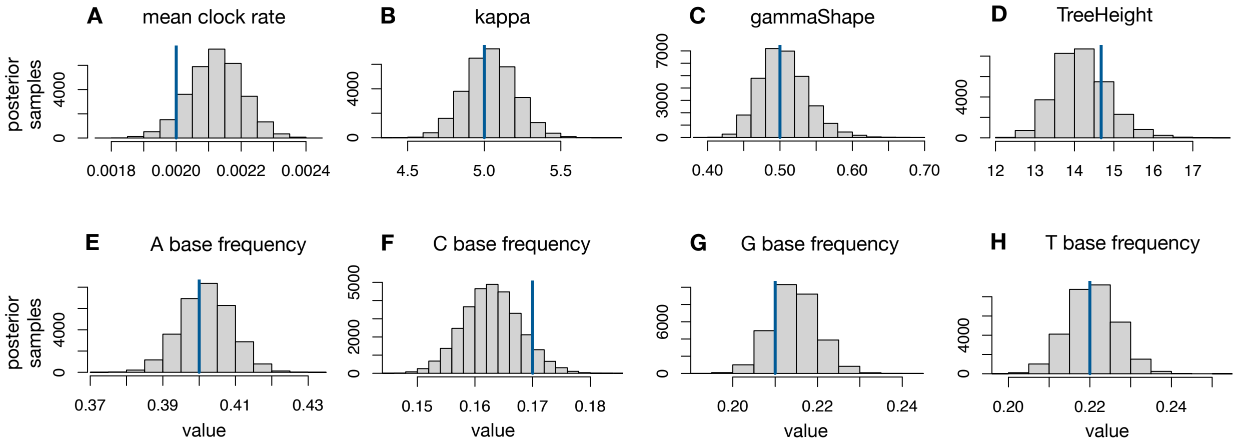

Figure S11: **Posterior distributions of molecular evolution parameters (BEAST2 run F1).** Posterior distributions of (A) the mean molecular clock rate, (B) the transition/transversion ratio κ, (C) the shape parameter of the discretized gamma distribution of substitution rates, (D) the height of the tree or root age, and (E–H) the stationary frequencies of nucleotide bases A,C,G,T, as inferred from 590 sequences simulated under a hypothetical birth-death-sampling scenario using BEAST2 (epidemiological parameters in Supplemental Fig. S6, tree in Supplemental Fig. S3A). Histogram bars show frequencies. Blue vertical lines show the true values used in the simulation.

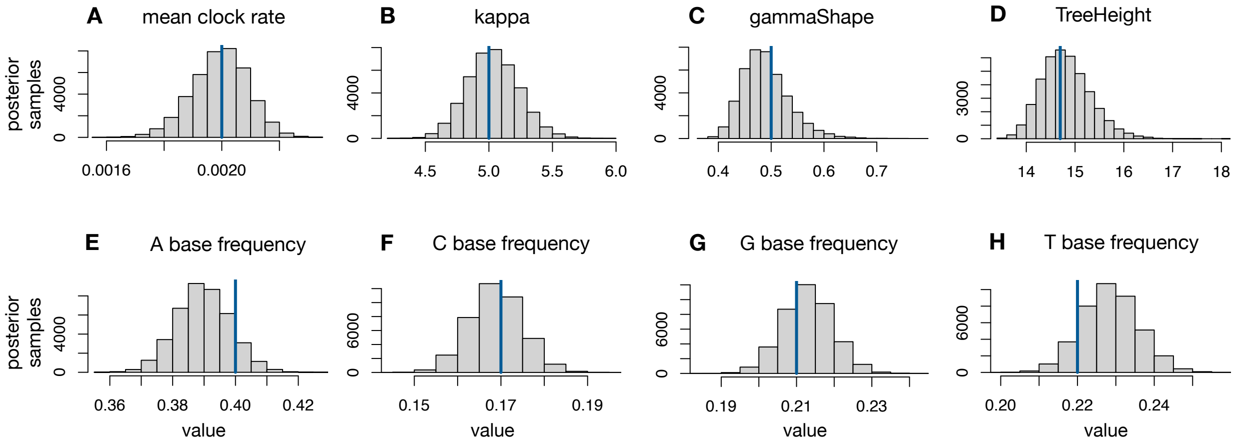

Figure S12: **Posterior distributions of molecular evolution parameters (BEAST2 run U2).** Posterior distributions of (A) the mean molecular clock rate, (B) the transition/transversion ratio κ, (C) the shape parameter of the discretized gamma distribution of substitution rates, (D) the height of the tree or root age, and (E–H) the stationary frequencies of nucleotide bases A,C,G,T, as inferred from 540 sequences simulated under a hypothetical birth-death-sampling scenario using BEAST2 (epidemiological parameters in Supplemental Fig. S7, tree in Supplemental Fig. S3B). Histogram bars show frequencies. Blue vertical lines show the true values used in the simulation.

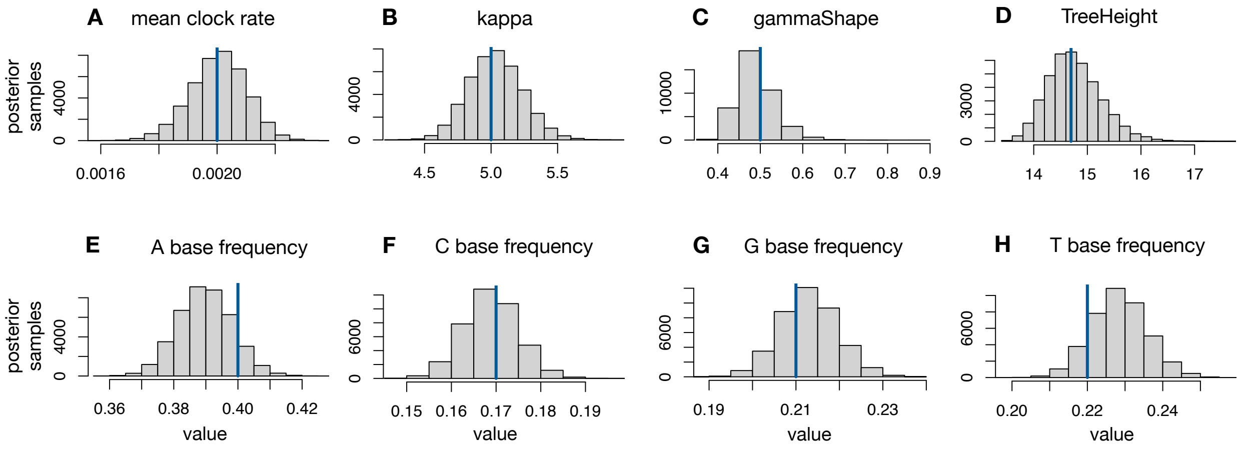

Figure S13: **Posterior distributions of molecular evolution parameters (BEAST2 run C2).** Posterior distributions of (A) the mean molecular clock rate, (B) the transition/transversion ratio κ, (C) the shape parameter of the discretized gamma distribution of substitution rates, (D) the height of the tree or root age, and (E–H) the stationary frequencies of nucleotide bases A,C,G,T, as inferred from 540 sequences simulated under a hypothetical birth-death-sampling scenario using BEAST2 (epidemiological parameters in Supplemental Fig. S9, tree in Supplemental Fig. S3B). Histogram bars show frequencies. Blue vertical lines show the true values used in the simulation.

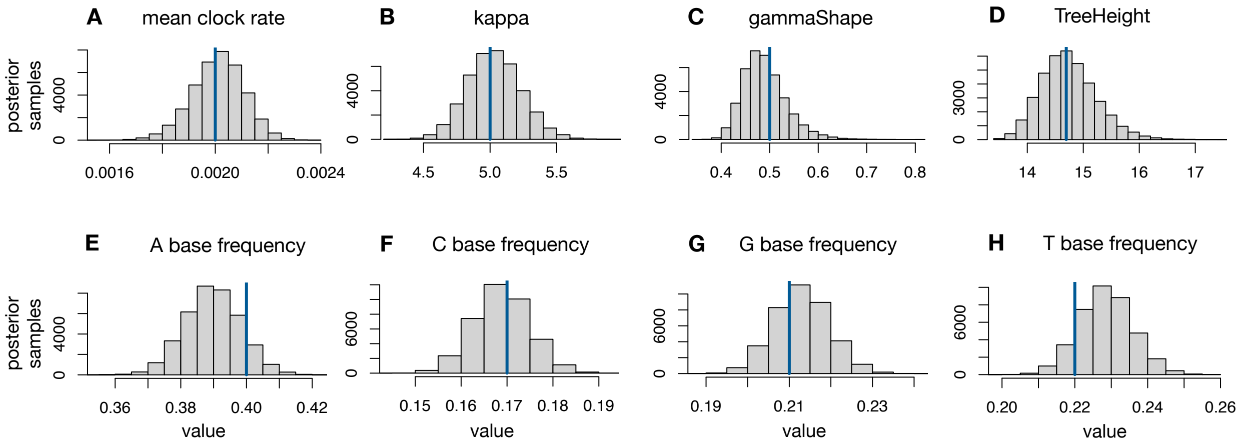

Figure S14: **Posterior distributions of molecular evolution parameters (BEAST2 run F2).** Posterior distributions of (A) the mean molecular clock rate, (B) the transition/transversion ratio κ, (C) the shape parameter of the discretized gamma distribution of substitution rates, (D) the height of the tree or root age, and (E–H) the stationary frequencies of nucleotide bases A,C,G,T, as inferred from 540 sequences simulated under a hypothetical birth-death-sampling scenario using BEAST2 (epidemiological parameters in Supplemental Fig. S8, tree in Supplemental Fig. S3B). Histogram bars show frequencies. Blue vertical lines show the true values used in the simulation.

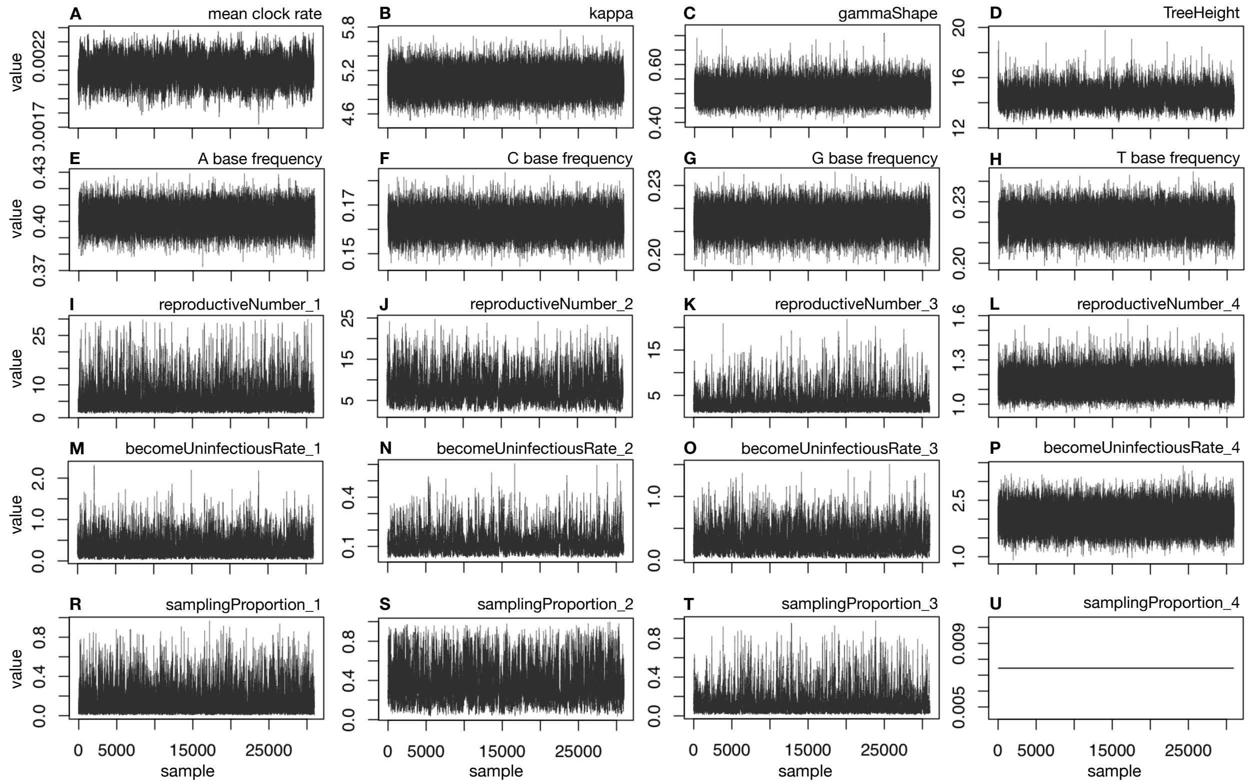

Figure S15: **MCMC traces of molecular evolution and epidemiological parameters (BEAST2 run U1).** MCMC trace plots of molecular evolution and epidemiological (birth-death-sampling skyline model) parameters generated by BEAST2 (2 independent MCMC chains), based on the sequences simulated under the 1st hypothetical birth-death-sampling scenario (epidemiological parameters in Supplemental Fig. S5, tree in Supplemental Fig. S3A). Samples are shown after burn-in removal and after thinning of each MCMC chain, and after concatenating the two MCMC chains.

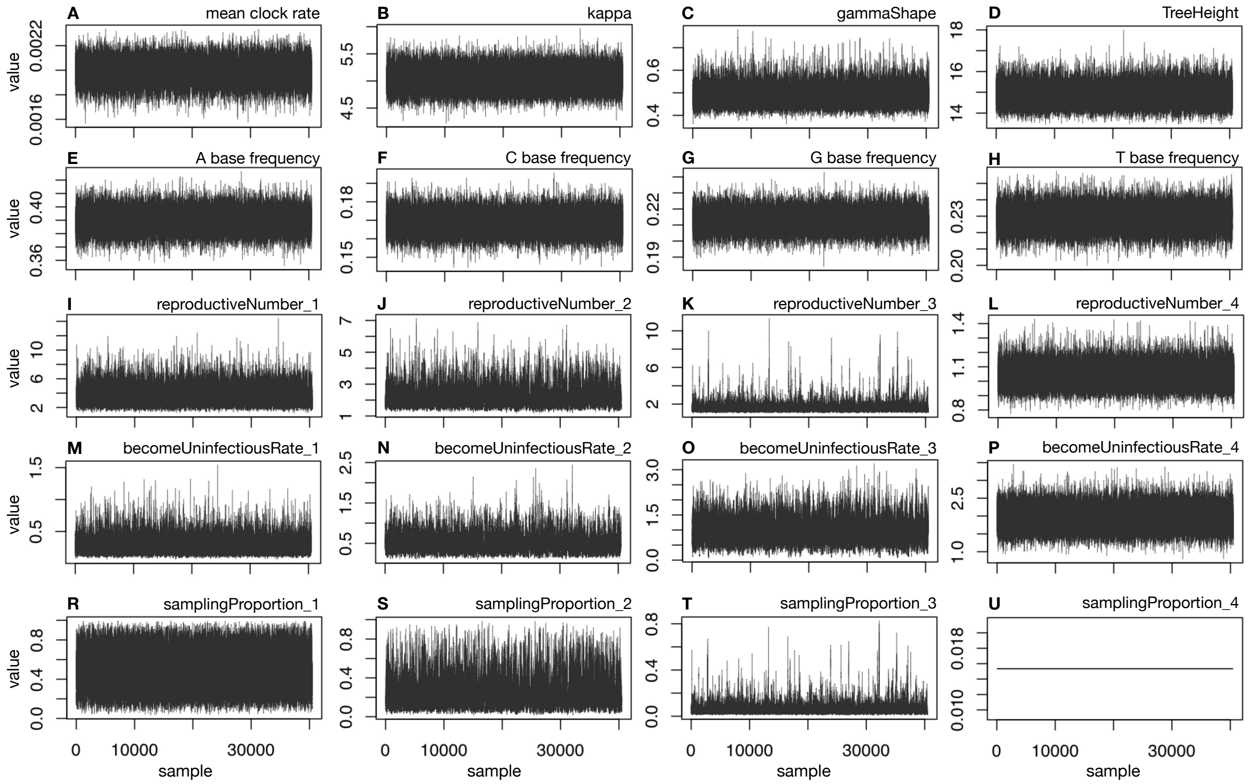

Figure S16: **MCMC traces of molecular evolution and epidemiological parameters (BEAST2 run U2).** MCMC trace plots of molecular evolution and epidemiological (birth-death-sampling skyline model) parameters generated by BEAST2 (2 independent MCMC chains), based on the sequences simulated under the 2nd hypothetical birth-death-sampling scenario (epidemiological parameters in Supplemental Fig. S7, tree in Supplemental Fig. S3B). Samples are shown after burn-in removal and after thinning of each MCMC chain, and after concatenating the two MCMC chains.

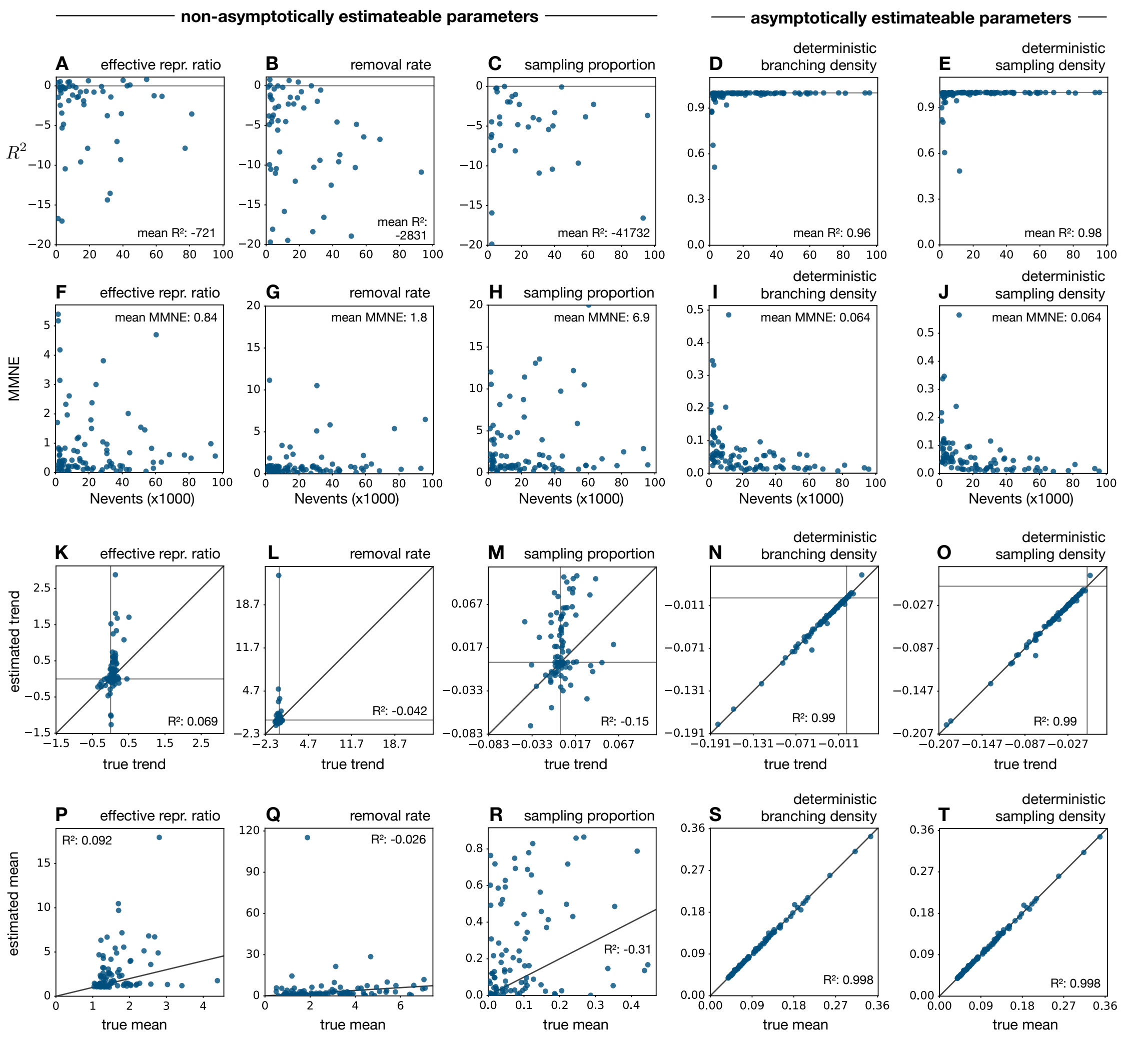

Figure S17: **Estimation accuracies using 92 simulated trees (exponential scenarios).** Figures summarize the results of fitting BDS models with piecewise-linear profiles for λ, μ and ψ to simulated trees, via maximum likelihood and choosing the grid size based on AIC. In each figure, each point corresponds to a different tree and its best fitted model. Trees were simulated under random epidemiological scenarios in which each λ, μ and ψ had simple exponential forms over time. Each tree comprised between 500 and 50,000 tips and was adequately described by the fitted model (according to Kolmogorov-Smirnov tests for the distribution of node ages, tip ages and edge lengths, $P > 0.05$ in all cases). Note that BDS skyline models were also fitted to the same trees, but almost always achieved a worse AIC than the piecewise linear models. (A) Coefficient of determination (R^2, vertical axis) for the estimated effective reproduction ratio R_e compared to the true R_e. Cases with R^2 below -20 are not shown. The average R^2 across all 92 trees is written in the figure. The horizontal axis denotes the total number of events in the tree (thousands of tips + branching points). (B-E) Similar to (A) and for the same trees and models, but showing the R^2 of the fitted removal rate (δ), sampling proportion (S), deterministic branching density ($\tilde{\beta}$) and deterministic sampling density ($\tilde{\sigma}$). Note the much higher R^2 for $\tilde{\beta}$ and $\tilde{\sigma}$, consistent with the fact that these are asymptotically estimateable from phylogenetic data. (F–J) Similar to A–E, but showing the mean modulus of the normalized error (MMNE) of the fitted parameters on the vertical axis instead of their R^2. MMNEs above 20 are not shown. The average MMNE across all 92 trees is written in the figure. Again, note the much lower MMNE for $\tilde{\beta}$ (I) and $\tilde{\sigma}$ (J). (K) Estimated linear trend (vertical axis) of R_e, compared to its true linear trend (horizontal axis), for the same fitted models as in A. Units are 1/year. The diagonal is shown for reference; the R^2 of the diagonal is written in the figure (a value of 1 would mean a perfect agreement between estimated and true trends). Linear trends were calculated from the fitted and true R_e profiles using linear regression with intercept. (L–O) Similar to K, but for other model parameters. (P–T) Similar to K–O, but comparing the true versus estimated mean parameter values. The diagonal is shown for reference; the R^2 of the diagonal is written in the figure. For concrete simulation examples see Supplemental Fig. S18. Methods details in Supplement S.3.

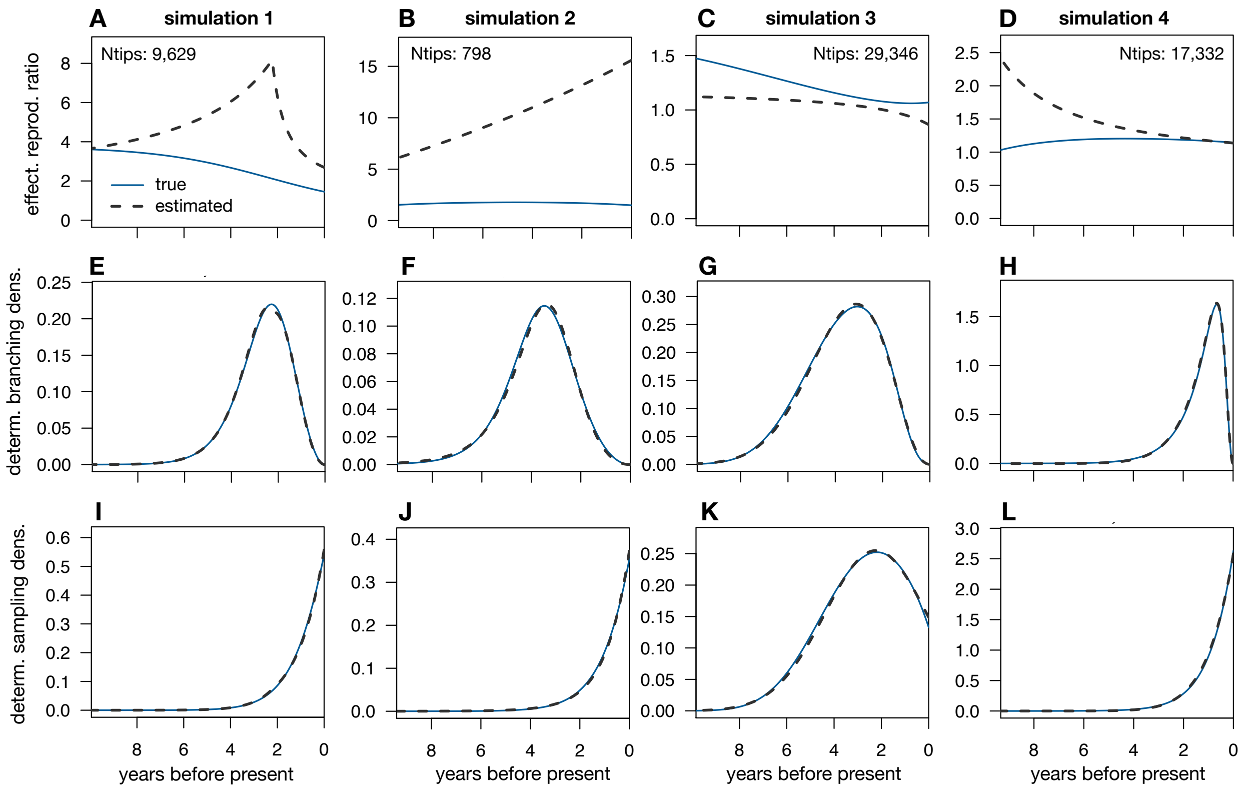

Figure S18: **Examples of R_e fitted to simulated trees (4 exponential scenarios).** (A) Estimated effective reproduction ratio R_e over time (dashed curve), estimated by fitting a BDS model with piecewise-linear profiles for λ, μ and ψ to a tree generated under a hypothetical epidemiological scenario as in Supplemental Fig. S17. The true R_e is shown for comparison (continuous curve). The number of tips in the tree is written in the figure. (B–D) As in A, but for different simulations. (E–H) Estimated and true deterministic branching densities ($\tilde{\beta}$), corresponding to the model fits in A–D. (I–L) Estimated and true deterministic sampling densities ($\tilde{\sigma}$), corresponding to the model fits in A–D. Note the good agreement between the estimated and true $\tilde{\beta}$ and $\tilde{\sigma}$, and the general bad agreement between the estimated and true R_e, showing that the fitted models accurately inferred the true scenario's congruence class but not the true scenario itself. The fitted models adequately described the trees (i.e., could not be rejected), based on Kolmogorov-Smirnov tests for the distribution of node ages, tip ages and edge lengths, ($P > 0.05$ in all cases). Methods details in Supplement S.3.

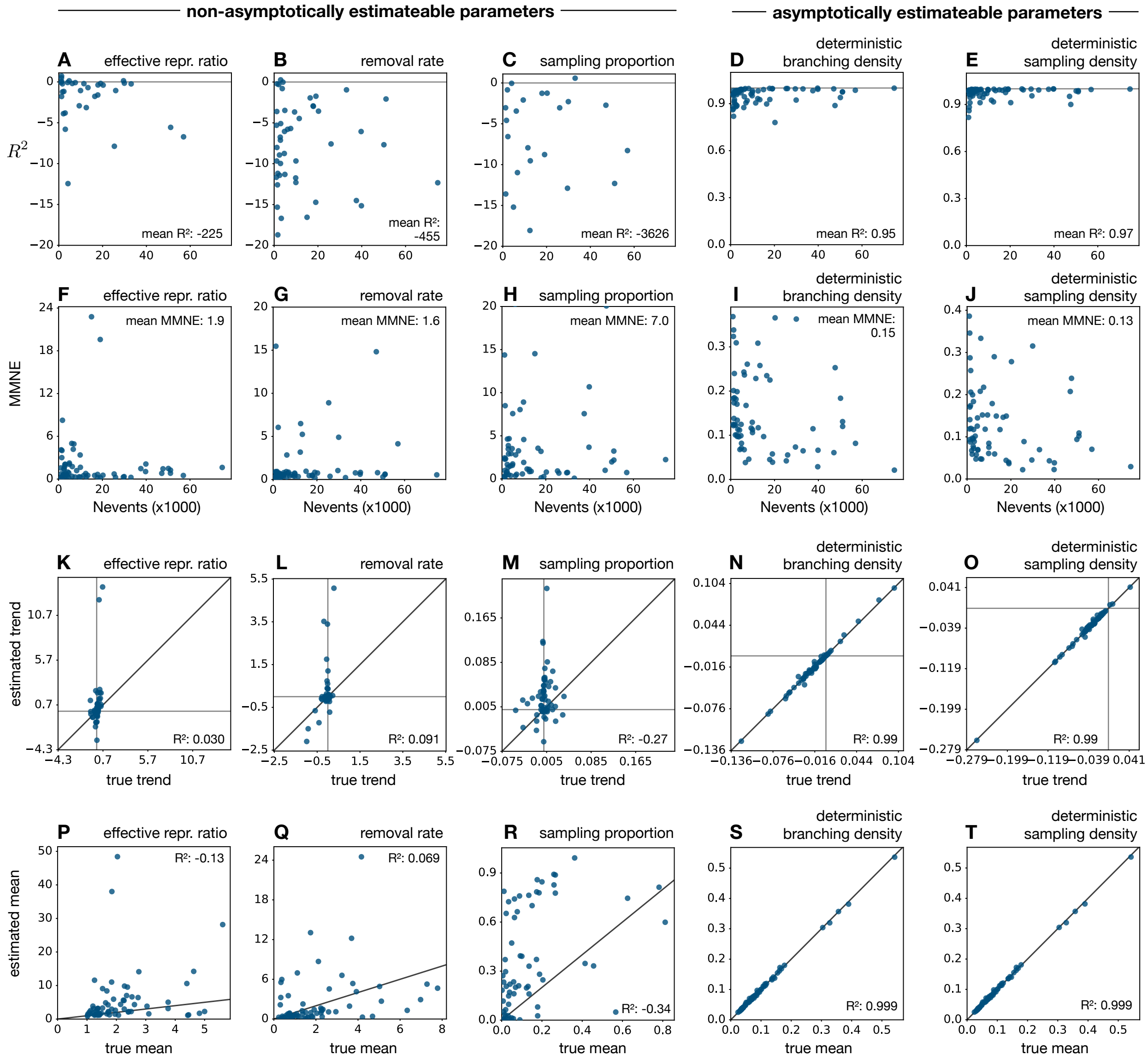

Figure S19: **Estimation accuracies using 66 simulated trees (Ornstein-Uhlenbeck scenarios).** Panels are as in Supplemental Fig. S17, but for trees simulated under epidemiological scenarios with Ornstein-Uhlenbeck-type profiles of λ, μ and ψ. For concrete simulation examples see Supplemental Fig. S20. Methods details in Supplement S.3.

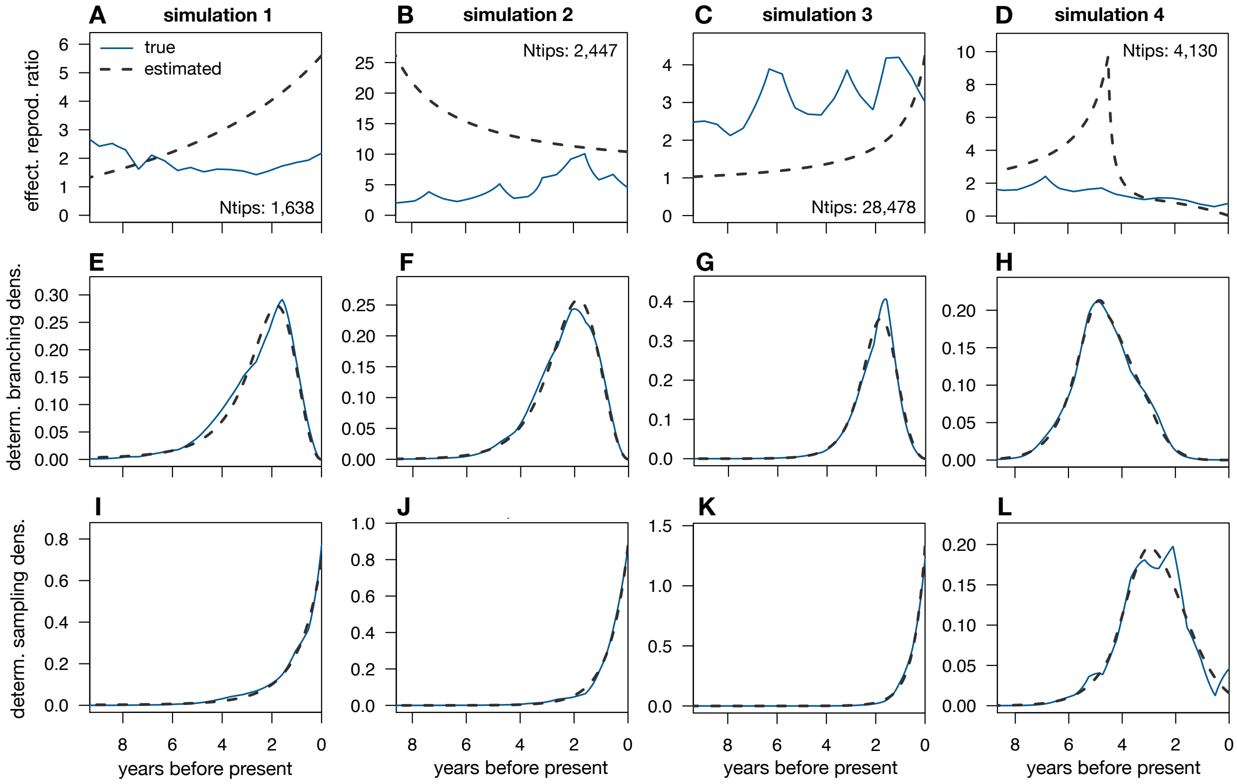

Figure S20: **Examples of R_e fitted to simulated trees (4 Ornstein-Uhlenbeck scenarios).** (A) Estimated effective reproduction ratio R_e over time (dashed curve), estimated by fitting a BDS model with piecewise-linear profiles for λ, μ and ψ to a tree generated under a hypothetical epidemiological scenario as in Supplemental Fig. S19. The true R_e is shown for comparison (continuous curve). The number of tips in the tree is written in the figure. (B–D) As in A, but for different simulations. (E–H) Estimated and true deterministic branching densities ($\tilde{\beta}$), corresponding to the model fits in A–D. (I–L) Estimated and true deterministic sampling densities ($\tilde{\sigma}$), corresponding to the model fits in A–D. Note the good agreement between the estimated and true $\tilde{\beta}$ and $\tilde{\sigma}$, and the general bad agreement between the estimated and true R_e (including wrong trends and major spurious features), showing that the fitted models accurately inferred the true scenario's congruence class but not the true scenario itself. The fitted models adequately described the trees (i.e., could not be rejected), based on Kolmogorov-Smirnov tests for the distribution of node ages, tip ages and edge lengths, ($P > 0.05$ in all cases). Methods details in Supplement S.3.

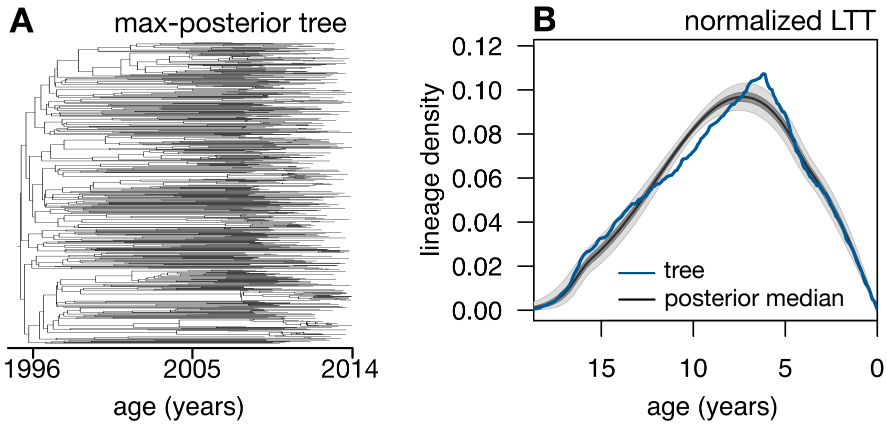

Figure S21: **HIV tree and LTT.** (A) Maximum-posterior-probability HIV timetree sampled with BEAST2. (B) Lineages-through-time (LTT) curve of the tree normalized to have unit area under-the-curve (blue curve), compared to the normalized deterministic LTTs of the posterior skyline models (black curve shows median, dark and light shades show 50% and 95% equal-tailed credible intervals).

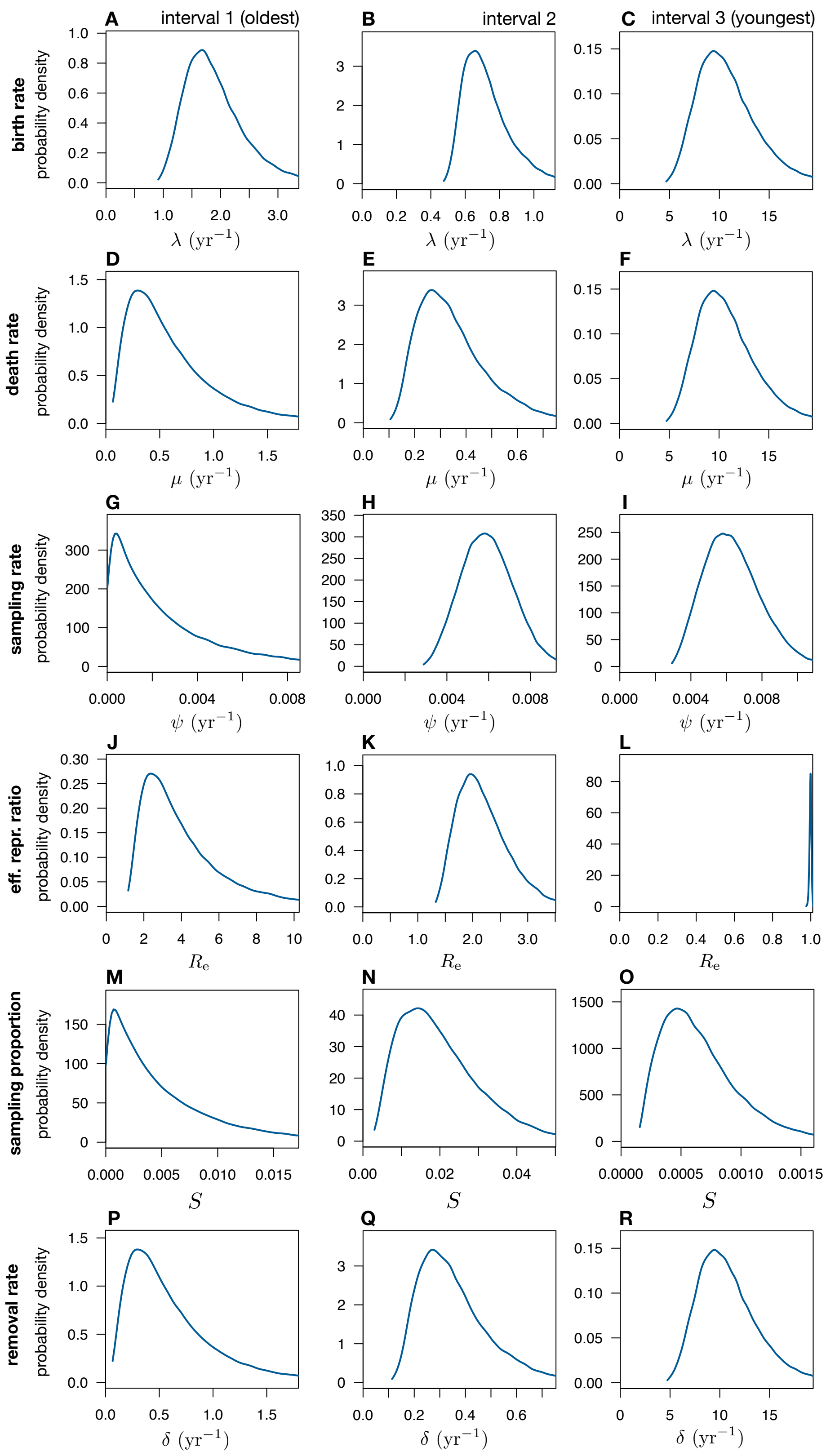

Figure S22: **HIV sampled posteriors - probability densities.** Probability densities of various epidemiological parameters according to the posterior model distribution sampled by BEAST2, in each of the 3 time intervals. Time intervals cover the periods 0–4, 4–16 and ≥16 years before present.

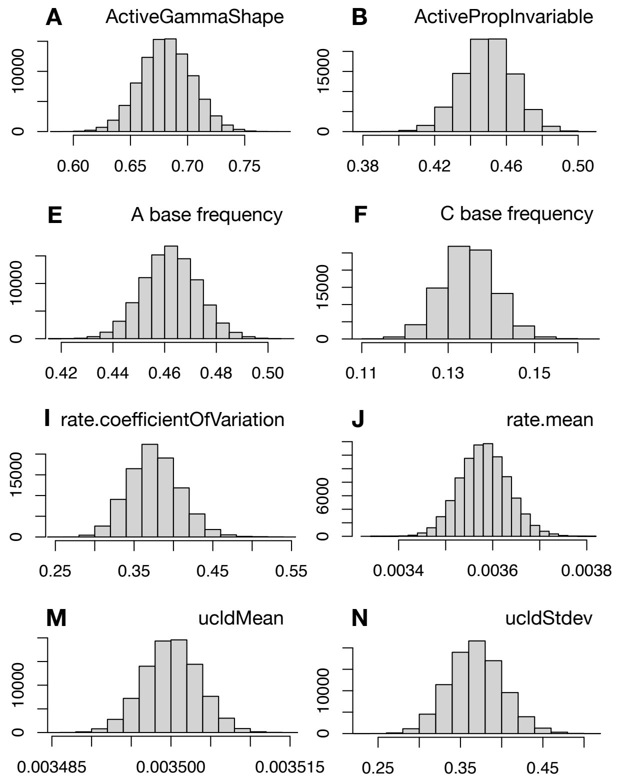

Figure S23: **Posterior distributions of molecular evolution parameters (HIV).** Histograms of various molecular evolution parameters drawn from the posterior distribution using BEAST2, for the HIV dataset. Vertical bars depict frequencies.

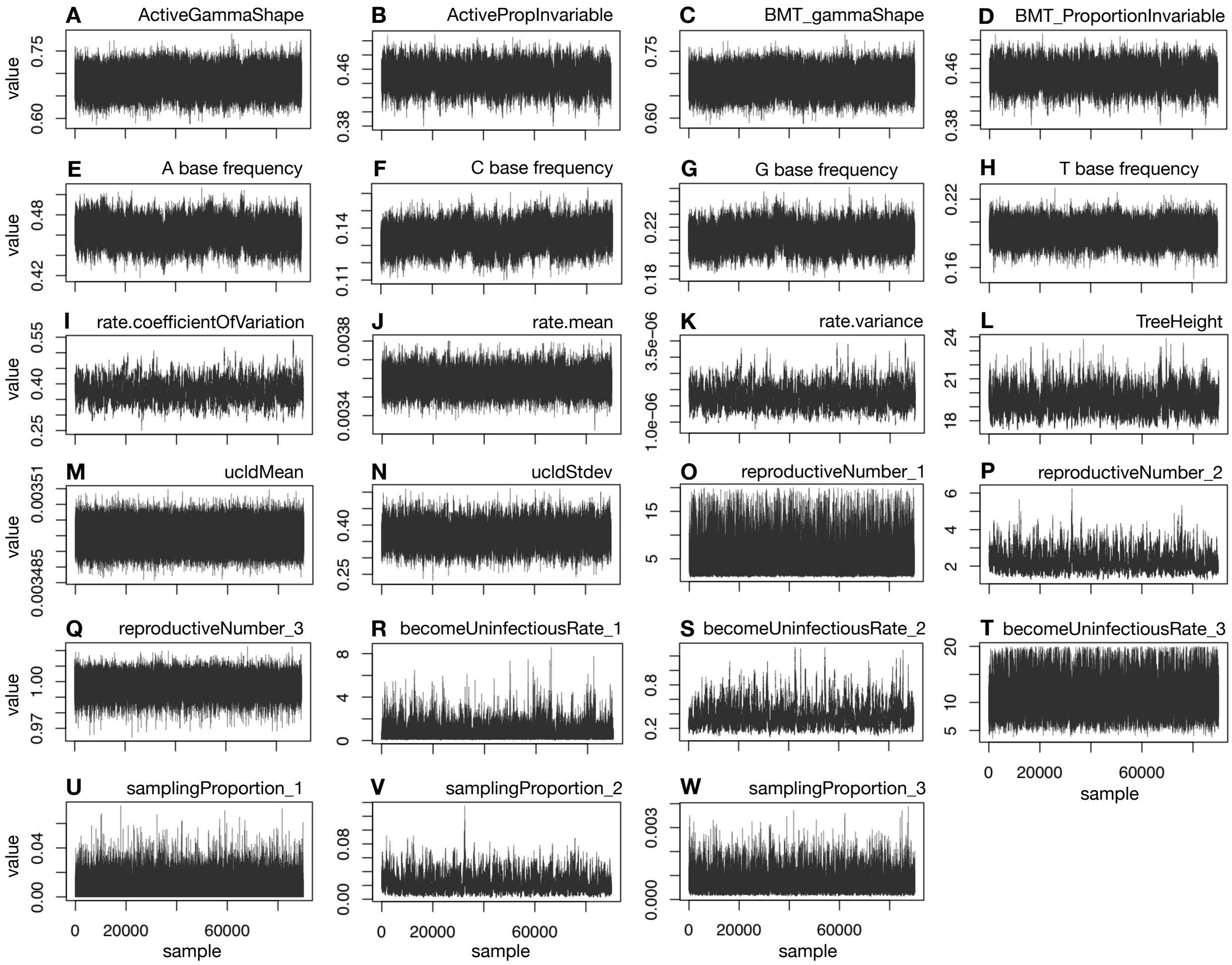

Figure S24: **MCMC traces of molecular evolution and epidemiological parameters (HIV).** MCMC trace plots of molecular evolution and epidemiological (birth-death-sampling skyline model) parameters generated by BEAST2, for the HIV dataset (2 independent MCMC chains). Samples are shown after burn-in removal and after thinning of each MCMC chain, and after concatenating the two MCMC chains.

Table S1: Priors and other settings specified for the BEAST2 analysis of the simulated HIV timetrees. * denotes that the prior parameters are in linear space (default is log).

Tree model	BD skyline serial
Substitution model	HKY+ I + G
Gamma categories	4
Clock model	Strict molecular clock
Clock rate	LN(mu=0.003, s=1)*
Reproductive number	LN(mu=0, s=1.25); 4 intervals
Become uninfectious rate	LN(mu=2, s=1.25)*; 1 or 4 intervals
Sampling proportion	BETA(alpha=2, beta=2); 4 intervals
Rate change times	2, 4, 8 years before present
Proportion invariant	BETA(alpha=2, beta=2)
Origin	LN(mu=20, s=1.25)*
Frequency parameter	UNIF(0-1), estimated
Gamma shape	EXP(1, offset=0)
Kappa	LN(mu=1, s=1.25)
Number of chains	2
Chain length	100–200 million
Sampling frequency	10 000

Table S2: Priors and other settings specified for the BEAST2 analysis of the empirical HIV-1 data from Northern Alberta. * denotes that the prior parameters are in linear space (default is log).

Tree model	BD skyline serial
Substitution model	bModelTest, Dirichlet (1, 1, 1, 1)
Clock model	Uncorrelated log normal relaxed
Clock mean	LN(mu=0.0035, s=0.001)*
Clock std.dev.	Gamma(alpha=0.5396, beta=0.3819)
Reproductive number	LN(mu=0, s=1.25); 3 intervals
Become uninfectious rate	LN(mu=0, s=1.25); 3 intervals
Sampling proportion	BETA(alpha=1, beta=100); 3 intervals
Rate change times	4, 16 years before most recent sample (2014)
Proportion invariant	BETA(alpha=1, beta=4)
Origin	LN(mu=25, s=1)*
Frequency parameter	UNIF(0-1), estimated
Gamma shape	EXP(1, offset=0)
Kappa	LN(mu=1, s=1.25)
Number of chains	2
Chain length	500 million
Sampling frequency	10 000

References

[1] MacPherson, A., Louca, S., McLaughlin, A., Joy, J. B. & Pennell, M. W. A general birth-death-sampling model for epidemiology and macroevolution. *bioRxiv* 2020.10.10.334383 (2020).

[2] Stadler, T., Kühnert, D., Bonhoeffer, S. & Drummond, A. J. Birth–death skyline plot reveals temporal changes of epidemic spread in HIV and hepatitis C virus (HCV). *Proceedings of the National Academy of Sciences* **110**, 228–233 (2013).

[3] Louca, S. & Pennell, M. W. A general and efficient algorithm for the likelihood of diversification and discrete-trait evolutionary models. *Systematic Biology* **69**, 545–556 (2020).

[4] Louca, S. & Doebeli, M. Efficient comparative phylogenetics on large trees. *Bioinformatics* **34**, 1053–1055 (2018).

[5] Akaike, H. Likelihood of a model and information criteria. *Journal of Econometrics* **16**, 3–14 (1981).

[6] Schwarz, G. Estimating the dimension of a model. *Annals of Statistics* **6**, 461–464 (1978).

[7] Engl, H. W., Hanke, M. & Neubauer, A. *Regularization of inverse problems*, vol. 375 (Springer Science & Business Media, 1996).

[8] Oraintara, S., Karl, W. C., Castanon, D. A. & Nguyen, T. Q. A method for choosing the regularization parameter in generalized tikhonov regularized linear inverse problems. In *Image Processing, 2000. Proceedings. 2000 International Conference on*, vol. 1, 93–96 (IEEE, 2000).

[9] Calvetti, D., Morigi, S., Reichel, L. & Sgallari, F. Tikhonov regularization and the L-curve for large discrete ill-posed problems. *Journal of Computational and Applied Mathematics* **123**, 423–446 (2000).

[10] Uhlenbeck, G. E. & Ornstein, L. S. On the theory of the Brownian motion. *Physical Review* **36**, 823–841 (1930).

[11] Gupta, A., Manceau, M., Vaughan, T., Khammash, M. & Stadler, T. The probability distribution of the reconstructed phylogenetic tree with occurrence data. *Journal of Theoretical Biology* **488**, 110115 (2020).

[12] Featherstone, L. A., Di Giallonardo, F., Holmes, E. C., Vaughan, T. G. & Duchêne, S. Infectious disease phylodynamics with occurrence data. *bioRxiv* (2020).

[13] Manceau, M., Gupta, A., Vaughan, T. & Stadler, T. The probability distribution of the ancestral population size conditioned on the reconstructed phylogenetic tree with occurrence data. *Journal of Theoretical Biology* **509**, 110400 (2021).
